# In Silico ApoE Isoform Interactions with Methylmercury (MeHg) and In Vivo MeHg Intoxication Effects on Epididymal White Fat Tissue and Liver Function in Young ApoE Knockout Mice

**DOI:** 10.1021/acs.chemrestox.5c00450

**Published:** 2026-05-05

**Authors:** Synara C. Lopes, Vitória K. Felix Monteiro, Paola Caroline L. Leocádio, Marcus V. F. Rodrigues, Mirna Maciel d’Auriol Souza, Maria José N. de Paiva, Flávia Zandonadi, Alessandra Sussulini, Ramon Raposo, Francisco Leomar da Silva, Dávila Zampieri, Antonio Augusto Coppi, Aline M. A. Martins, Jacqueline Alvarez-Leite, Ámison R. Lopes da Silva, Norberto de K. V. Monteiro, Reinaldo B. Oriá

**Affiliations:** 1 Laboratory of the Biology of Tissue Healing and Nutrition, Department of Morphology and Institute of Biomedicine, School of Medicine, 28121 Federal University of Ceara, Fortaleza, CE 60430-270, Brazil; 2 Department of Biochemistry and Immunology, 28114 Federal University of Minas Gerais, Belo Horizonte, MG 31270-901, Brazil; 3 Department of Analytical Chemistry and Physical Chemistry, 28121 Federal University of Ceara, Fortaleza, CE 60020-181, Brazil; 4 Department of Analytical Chemistry, Institute of Chemistry, 28132 State University of Campinas, Campinas, SP 13086-002, Brazil; 5 Experimental Biology Core, Health Sciences, 28128 University of Fortaleza, Fortaleza, CE 60811-905, Brazil; 6 12202 University of Bristol Faculty of Health Sciences, Bristol BS8 1UD, England, U.K.; 7 Integrated Space Stem Cell Orbital Research Center/Stanford Consortium for Regenerative Medicine, University of California, San Diego, La Jolla, California 92093, United States; 9 Federal University of the São Francisco Valley, Fortaleza, BA CE 60400-990, Brazil; 10 Department of Organic and Inorganic Chemistry, Federal University of Ceará, Fortaleza, CE 60400-990, Brazil

## Abstract

MeHg neurotoxicity is well recognized; however, less is known about its effects on metabolism. Here, we integrated in silico and in vivo approaches to investigate MeHg interactions with apolipoprotein E (ApoE) and their metabolic consequences. Computational analyses revealed that human ApoE2 and ApoE3 isoforms establish stable Hg–S bonds with MeHg, while ApoE4 showed no stable binding, suggesting reduced mercury retention capacity. In addition, we evaluated the impact of MeHg intoxication (20 mg/L in drinking water for 20 days) on the liver and epididymal white fat (EWF) in young ApoE knockout (ko) and wild-type mice. Hg levels in hair, liver, and EWF were measured. We also evaluated body weight gain, plasma triglycerides, total cholesterol, and liver injury by assessing steatosis score, SOD, TBAR, AST, and ALT activities. To evaluate EWF, we analyzed adipocyte diameter, plasma leptin levels, and metabolomics. Hg levels were markedly higher among intoxicated mice. ApoE ko mice had higher Hg concentrations in hair but lower levels in liver and EWF than wild-type controls. Among wild-type mice, MeHg compromised weight gain and increased liver TBAR compared to nonchallenged controls. Among nonintoxicated mice, ApoE deficiency significantly increased triglycerides, cholesterol, and liver transaminases, accompanied by reduced EWF wet weight, SOD activity, and leptin levels. MeHg, together with ApoE deficiency, elevated cholesterol, triglycerides, hepatic transaminases, and TBARS. We found distinct EWF metabolite activity in different scenarios of ApoE deficiency and MeHg intoxication, highlighting increased cardiovascular risk when both challenges occur. Further studies are needed to clarify these mechanisms and identify key nutritional interventions.

## Graphical abstract

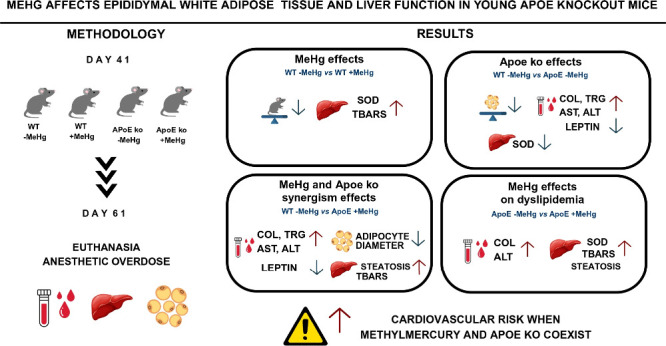

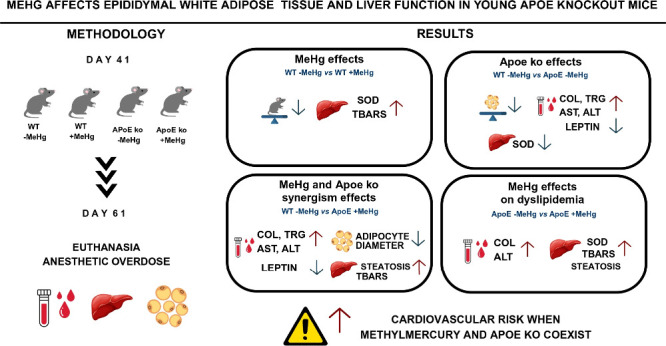

## 1 Introduction

Mercury (Hg) can be found in three most common forms in the environment: elemental (metallic Hg^0^), inorganic (Hg^+^ and Hg^2+^ as salts), and in organic compounds, such as methylmercury (MeHg) which are present in sediment, water, and the atmosphere.^1^ Artisanal mining activity is a primary source of Hg contamination, with Hg utilized to separate gold from other materials during mining.^2^ This activity leads to the accumulation of Hg in riverbeds, ultimately forming the highly toxic MeHg compound, furthermore natural and anthropogenic environmental disasters can drastically increase the exposure of human populations to MeHg poisoning,^3^ representing a worrisome health issue and a global concern.^4,5^

Consumed MeHg is rapidly absorbed by the body, widely distributed in tissues, and slowly excreted.^6^ Once consumed, MeHg easily crosses cellular barriers, including the placental and blood-brain barriers, with rapid access to the fetus and the central nervous system (CNS), respectively.^7^ Heavy metal intoxication affects the CNS, possibly contributing to obesity and its consequences.^5^

Some metabolic consequences of pollution in humans may be attributed to chronic heavy metal exposure, which has direct effects on adipose tissue, interfering with adipocyte signaling and releasing adipokines—a class of molecules associated with a wide range of biological functions.^8^ Chronic MeHg toxicity can also disrupt the adipose tissue, but its effects on fat metabolism are not well understood.^9,10^

Apolipoprotein E (ApoE) is a very important apolipoprotein involved in lipid metabolism. ApoE participates in the homeostatic control of plasma and tissue lipid contents, playing a critical role in cholesterol liver clearance.^11^ ApoE is mainly synthesized in hepatocytes and plays several immune system-modulating roles, affecting the development of inflammation-related chronic diseases.^12,13^

In this study, we evaluated in silico ApoE isoforms interaction with MeHg. Additionally, we investigated the effects of MeHg intoxication (20 mg/L in drinking water for 20 days) on the structural and functional alterations of the epididymal white adipose tissue (EWF) in ApoE knockout (ko) mice that show systemic ApoE deficiency and spontaneous dyslipidemia. ApoE ko mice have been used as an experimental model of cardiovascular disease and atherosclerosis,^14^ even at a young age.^15^ These findings of this study may shed light on novel mechanistic interactions of ApoE and MeHg and may guide potential interventions to reduce the impact of MeHg intoxication on the adipose tissue metabolism in dyslipidemic individuals.

## 2 Materials and Methods

### 2.1 Computational Analysis of MeHg-ApoE Interactions

To complement the experimental findings, a computational approach was conducted to investigate the molecular interactions between MeHg and the human ApoE isoforms (ApoE2, ApoE3, and ApoE4).

#### 2.1.1 Structural Modeling and System Preparation

The three-dimensional structures of the ApoE isoforms were obtained from the Protein Data Bank (PDB ID: 2L7B for ApoE3). The models of ApoE2 and ApoE4 were generated by replacing residues at positions 112 and 158, according to known polymorphisms, using Chimera v.1.18 software. The modified structures were inspected and subjected to initial energy minimization. An 8Å cutoff radius was applied in the region of interest for each protein using Visual Molecular Dynamics (VMD) software.^16^

The MeHg molecule was constructed and optimized using Avogadro software and, to determine the lowest energy structure, Density Functional Theory was employed, using Gaussian 09 software^17^ In this process, the B3LYP functional^18^ with the basis sets 6–31+g­(d,p)^19^ was applied to describe the carbon and hydrogen atoms and LanL2DZ^20^ to describe the mercury atom.

#### 2.1.2 Semiempirical Calculations

Interaction energies were calculated using MOPAC 22.1.1 software with the PM6-D3H4 semiempirical method,^21^ which includes dispersion and hydrogen bonding corrections appropriate for metal–ligand systems. CH_3_Hg was positioned near the residue of interest in each isoform, and the systems were optimized.

The interaction enthalpy (ΔH*int*) between MeHg and each ApoE isoform was calculated according to the following equation:

$$\Delta H\mathrm{Int}=\Delta \mathrm{HAPOE-}{\mathrm{CH}}_{3}\mathrm{Hg}-\left[\Delta \mathrm{HAPOE}+{\Delta \mathrm{HCH}}_{3}\mathrm{Hg}\right]$$

where ΔH*Int* is the interaction energy between the protein and the CH_3_Hg molecule, ΔHAPOE–CH_3_Hg represents the formation enthalpy of the protein–ligand complex, ΔHAPOE is the formation enthalpy of the isolated protein, and ΔHCH_3_Hg is the formation enthalpy of the isolated methylmercury molecule. Negative values of ΔH*Int* indicate favorable exothermic interactions, while positive values indicate unfavorable endothermic binding.

#### 2.1.3 Quantum Topological and Noncovalent Interaction Analyses

Single-point energy calculations were performed using Density Functional Theory (DFT)^22,23^ with the B3LYP functional.^23^ For the C, N, S, O, and H atoms, the 6–31+G­(d,p) basis set was used, while for the Hg atom, the LANL2DZ pseudopotential was used. These calculations were performed with Gaussian 09 software^17^

To characterize the electronic structure of the MeHg-ApoE interactions, topological analyses were performed based on the Quantum Theory of Atoms in Molecules (QTAIM)^24–27^ using Multiwfn v3.8. From these analyses, the Bond Critical Points (BCPs) and their respective bond paths were determined. For interactions involving the Hg atom and the groups sharing electron density with it, the values of the electron density (ρ), the Laplacian of the electron density (∇^2^ρ), and the Electron Localization Function (ELF) were extracted from the identified BCPs.

Additionally, calculations involving noncovalent interactions (NCIs) were performed, obtaining isosurfaces corresponding to the nature of the interactions to which the Hg atom is subjected in each of the analyzed systems. The Reduced Density Gradient (RDG) maps indicate, through indices and peaks in the RDG, the details of the isosurfaces on the geometries and interactions that Hg has in this 6 Å cutoff applied to ApoE.

This combined approach provided both quantitative and qualitative insights into the bonding environment of the Hg atom in each ApoE isoform, enabling the correlation of electronic topology with the interaction energies obtained in the semiempirical calculations.

### 2.2 In Vivo Experimental Design

Ninety-seven C57BL6J young males aged 41 days and weighing 25–30 g were obtained from the production vivarium of the Experimental Biology Core/University of Fortaleza (Unifor/NuBEx). Four groups were assigned: (1) wild-type controls, (2) MeHg-intoxicated wild-type mice, (3) ApoE ko controls, and (4) MeHg-intoxicated ApoE ko mice. All mice were housed in microisolators in temperature and humidity-controlled ventilators and received food and water *ad libitum*.

### 2.3 MeHg Chronic Intoxication

Experimental mice were intoxicated by 20 mg of MeHg/L (MeHg chloride, Sigma-Aldrich, St. Louis, MI, USA) in drinking water for 20 days, according to Andersen and Andersen^28^ MeHg solution was changed weekly. To prevent cross-contamination of MeHg in the vivarium, intoxicated mice were housed in a separate room. According to international guidelines, precautions were taken for animal and experimenter safety, as well as proper disposal of chemical waste.

### 2.4 Measuring Hg Concentration in Hair, Liver, and Epididymal White Fat

Dorsal skin hair/fur, liver, and epididymal white adipose tissue samples were harvested for total elemental mercury measurements using the Direct Mercury Analyzer (DMA-80, Milestone, Sorisole, BG, Italy), as previously described by Windmoller et al.^29^ Liver and adipose tissue samples were lyophilized before analyses. Results are expressed as mg/kg for hair and liver measurements and μg/kg for epididymal white adipose tissue.

### 2.5 Body Weight Gain

To address whether MeHg intoxication could be obesogenic, we assess body weight changes by monitoring both the weekly body weight gain adjusted by the initial weight (%) and the delta body weight gain (g), which is the difference between final body weight (D61) and initial body weight (baseline) at the start of the MeHg challenge.

### 2.6 Evaluation of Dyslipidemia

To assess whether chronic MeHg intoxication could lead to dyslipidemia, blood samples were harvested through the retro-orbital plexus and centrifuged for 10 min at 3500 rpm. Plasma was obtained to determine the concentration of total cholesterol and triglycerides. The concentrations were determined using an enzymatic colorimetric reaction. Plasma was added to the test tube, followed by the appropriate reagents. The mixture was incubated at 37 °C for 10 min. The absorbance was read at 500 nm using a spectrophotometer. Total cholesterol and triglyceride concentrations were calculated using the following equations: Cholesterol/triglyceride = (Absorbance of the sample/Absorbance of the standard) × 200. Results are expressed as mg/dL. The results were determined by a semiautomatic analyzer (LabQuest, Labtest, Brazil) using diagnostic kits (Labtest, Brazil). Results are expressed as mg/dL.

### 2.7 Evaluation of MeHg Liver Toxicity

#### 2.7.1 Liver Function

To evaluate MeHg-induced liver dysfunction, we measured AST and ALT activity using standard diagnostic kits (Labtest, Brazil). The AST and ALT activity was determined based on the enzymatic colorimetric method. Appropriate reagents were added to a tube, followed by the addition of plasma. The mixtures were incubated at 37 °C for 1 min. The initial absorbance was recorded, and after 2 min, the final absorbance was recorded to perform the calculations indicated by the kits. The absorbance was read at 340 nm using a spectrophotometer. Results were expressed in AST and ALT (U/L).

#### 2.7.2 Oxidative Stress

To measure oxidative stress, we analyze liver superoxide dismutase (SOD) and thiobarbituric acid-reactive substances (TBARS) activity. For SOD measurements, samples were homogenized in phosphate buffer and pyrogallol, and the oxidizing agent MTT (dimethyl thiazole-diphenyltetrazolium bromide) was added. The reaction was determined spectrophotometrically by absorbance at 570 nm. For calculation, we assumed that 1 unit (U) of SOD could prevent the auto-oxidation of 50% of pyrogallol in the standard. The result was expressed in SOD units per mg of protein (U/mg protein).

TBARS assessment, samples were homogenized in ice-cold 1x PBS and centrifuged for 10 min at 12,000 rpm. 500 μL of a solution containing trichloroacetic acid (TCA 15%), thiobarbituric acid (TBA 0.0375%), and hydrochloric acid (HCl 0.25 N) were added to the supernatants (250 μL). The samples were kept in a boiling water bath for 15 min and then placed in running water until they cooled down. 750 μL of butyl alcohol was added and mixed. Samples were centrifuged for 10 min at 3,000 rpm at room temperature. 200 μL of the supernatant was added to the 96-well plate in duplicate. The absorbance was measured at a wavelength of 535 nm. Results were normalized by the protein concentration in the tissue^30^

#### 2.7.3 Hepatic Steatosis Scoring

Slides were processed, stained in hematoxylin and eosin (H&E), and blindly classified by a pathologist considering steatohepatitis and ballooning scores.^31^ The steatohepatitis score was according to the following classification: (0) no conspicuous steatohepatitis, (1) borderline steatohepatitis, (2) detectable steatohepatitis. Ballooning score graduation was: (0) none, (1) few ballooned cells, (2) many ballooned cells/prominent ballooning.

### 2.8 MeHg Effects on Epididymal White Fat Structure and Function

#### 2.8.1 Epididymal Adipose Tissue Weight and Morphometry

The weight of epididymal adipose tissue was evaluated shortly after mice euthanasia with the aid of an analytical scale (Filizola, SP, Brazil) to assess possible alterations between the studied groups. Results were expressed in mg of wet tissue. After weighing, EWF was fixed in 4% formaldehyde for 1 to 2 days. Following this, samples were embedded in paraffin, sectioned at 5 μm, and stained with hematoxylin/eosin.

Adipocyte diameter was assessed in digitalized images using a CX31 Olympus light microscope equipped with a QCapture image analysis software package. The adipocyte diameters were measured from ten random 10x-objective magnification fields per slide. Three slides per animal were analyzed, and nonconsecutive slides were taken. For cell diameter measurements after proper calibration, ImageJ 1.45 software (National Institutes of Health, Bethesda, MD, USA) was used.

### 2.9 Leptin Plasma Levels by ELISA Immunoassay

Blood was collected through the retro-orbital plexus, kept in heparin-coated capillary tubes, and centrifuged for 10 min at 3500 rpm. The supernatant was obtained and immediately stored in a −20 °C freezer for later analysis. Samples were analyzed following the instructions of the R&D standard protocols for the ELISA leptin duo set assay kit.

### 2.10 Lipidomics Assessment

#### 2.10.1 Sample Preparation

Tissues were weighed, frozen in liquid nitrogen, and macerated in 2 mL tubes, which were submitted to the SIMPLEX protocol^32^ for extraction and separation of lipids. Initially, 225 mL of ice-cold methanol was added to the tubes. Then, the tubes were vortexed for 20 min and sonicated for 10 min. After homogenization, 750 μL of ice-cold MTBE and 188 μL of 0.1% (m/v) aqueous ammonium acetate solution were added. The tubes were then centrifuged for 15 min at 10,000 g at 4 °C. The upper phases were collected, filtered with 0.22 μm nylon syringe filter, and placed in new 1.5 mL tubes. Subsequently, the solutions in these tubes were dried in a SpeedVac (Concentrator PlusTM, Eppendorf, Hamburg, Germany) at 30 °C for 2h. After this step, the samples were resuspended in a 60:40 (v/v) acetonitrile (ACN): water solution, vortexed, and centrifuged. The supernatant was transferred to vials with 250 μL inserts.

#### 2.10.2 Liquid Chromatography and Mass Spectrometry Conditions

Chromatographic separation was performed in an ultrahigh-performance liquid chromatography system (UltiMate 3000 RSLCnano system, Thermo Scientific, Waltham, MA, USA). The separation was performed using a Titan C18 2.1 mm × 100 mm x 1.7 μm column from Supelco (Sigma-Aldrich, Darmstadt, Germany). The sample injection volume was set to 5 μL. The temperature was maintained at 40 °C, and the separation was carried out at a flow rate of 0.25 mL min^–1^ under a gradient in which the mobile phases consisted of (A) 60:40 (v/v) ACN: water and (B) 90:10 (v/v) isopropanol: ACN. For ESI negative and positive modes, 10 mmol L^–1^ ammonium formate was added to both mobile phases.

The gradient for the ESI negative mode analyses started with 40% B for 2 min, increasing to 50% B from 3 to 6 min, and 70% B from 6.1 to 8 min. Over the next 2 min, the gradient was maintained at 100% B, then 40% B for 3 min. For ESI positive mode analyses, the gradient stopped at 9–11 min at 100% B. Detection and acquisition were performed using a Q-Exactive Plus (Thermo Fisher Scientific, Waltham, MA, USA) mass spectrometer employing ESI in positive and negative modes in a full scan range from *m*/*z* 100 to 1500, according to the methodology described by Lísa and collaborators.^33^ Briefly, the source temperature was set to 300 °C, and the capillary voltage was set to -3.2 or +3.5 kV. The Orbitrap mass analyzer was set to 1 scan/s at 70,000 resolution and an injection time of 100 ms. Full MS spectra were acquired for each sample. Data-dependent analysis (DDA) TopN 5 DD-MS2 was used for the acquisition of the MS/MS spectra and performed in one-tenth of the samples. This method consists of detecting the most abundant ion species in the primarily acquired full MS spectra. These five ion species are consequently fragmented by higher collision-induced dissociation in the next scan (*m*/*z* 200 to 2000 MS/MS scan range) at 17,500 resolution.

#### 2.10.3 Data Processing

The data obtained in raw format was converted to .ABF format, using the Analysis Base File Converter software (https://www.reifycs.com/abfconverter/), to be imported into the MS-DIAL 4.9 software (https://systemsomicslab.github.io/compms/msdial/main.html) to perform preprocessing and alignment. When importing the files, tolerance values for MS1 and MS2 of 0.01 and 0.025 were used, respectively. Furthermore, the retention time ranges from 0 to 14 min, with a mass detection range from 100 to 1500 Da, according to data acquisition constraints. To filter only the relevant ions and reduce the influence of noise, the minimum height of the peaks was defined as 1 × 10^7^. For identification, precise mass tolerance values equal to 0.01 for MS1 and 0.05 for MS2 were used (with an 80% cutoff).

After alignment, the data was exported in .txt format, generating a feature table with a combination of retention time and *m*/*z* for each molecular feature (MF). The intensity detected in each sample was also evaluated. During exporting, fields with a value equal to zero were filled in with a value equal to 1/10 of the minimum intensity of the peaks in the samples (data completeness) using tools embedded in the software. The identification of the results detected and selected by the configured parameters was carried out using the MS-Finder 3.6 software (http://prime.psc.riken.jp/compms/msfinder/main.html), using the various available databases.

MetaboAnalyst, an online platform available for data analysis in omics approaches (https://www.metaboanalyst.ca/MetaboAnalyst/home.xhtml) was used for multivariate statistical analyses. The matrix was uploaded to the platform and evaluated by the following multivariate methods: Principal Component Analysis (PCA) and supervised techniques (PLS-DA and oPLS-DA). Peak values were normalized using mean values and transformed to log 10. The normalized matrix was evaluated using Hierarchical Cluster Analysis (HCA), carried out using the Ward grouping method with Euclidean distance, and these same parameters were used to generate the heatmaps.

### 2.11 Statistical Analysis

The GraphPad Prism statistical package was used in all analyses (GraphPad Prism version 10.1, San Diego, CA, USA). Results were expressed as mean ± standard error of the mean (SEM). Statistical comparisons and significance were verified using a two-way ANOVA with a Bonferroni post hoc test. We used either the Kruskal–Wallis test or the Mann–Whitney test when appropriate for the histopathological scoring analyses. A *p* < 0.05 value was considered significant.

### 2.12 Ethics Statement

All experimental procedures involving animals were approved by the Animal Ethics Committee (CEUA) of University of Fortaleza (UNIFOR), under protocol number 4831110618. All animal procedures were conducted by the ethical standards and guidelines established by the National Institutes of Health (NIH, USA) for the Care and Use of Laboratory Animals (National Research Council, 2011). The study was also reported following the ARRIVE (Animal Research: Reporting of In Vivo Experiments) guidelines to ensure transparent and reproducible animal research.

## 3 Results

### 3.1 In Silico Analysis of MeHg-ApoE Interactions

Semiempirical calculations revealed distinct energetic and structural behaviors among ApoE isoforms upon CH_3_Hg binding (Table 1). ApoE2 and ApoE3 isoforms, which contain cysteine residues, exhibited negative interaction enthalpies, indicating stable complex formation. The most stable interactions were observed for ApoE2-Cys112 (−128.41 kcal mol^–1^) + ApoE2-Cys158 (−150.50 kcal mol^–1^), followed by ApoE3 (−191.18 kcal mol^–1^). In contrast, the ApoE4 isoform, which lacks cysteine residues, exhibited a positive interaction energy (+64.80 kcal mol^–1^), suggesting unfavorable binding and structural instability of the ApoE4-CH_3_Hg complex.

**Table 1:** Interaction Energy Values of the Sets ApoE-CH_3_Hg and ApoE + CH_3_Hg, and the Interaction between the Two Components[Table-fn t1fn2]

**isoform**	**ApoE energy**(kcal/mol)	**CH**_**3**_ **Hg energy**(kcal/mol)	**ApoE + CH**_**3**_ **Hg energy**(kcal/mol)	**ApoE-CH**_**3**_ **Hg system**(kcal/mol)	**interaction energy**(kcal/mol)
ApoE2 (CYS 112)	–2056.09	224.40	–1831.69	–1960.10	–128.41
ApoE2 (CYS 158)	–2941.6	224.40	–2717.20	–2867.70	–150.50
ApoE3	–2973.22	224.40	–2748.82	–2940.00	–191.18
ApoE4	–3036.47	224.40	–2812.07	–2747.27	64.80

a ApoE energy = energy value calculated for the isolated isoform; CH_3_Hg energy = fixed value corresponding to methylmercury (224.40 kcal/mol); ApoE + CH_3_Hg energy = sum of the energies of the isoform and CH_3_Hg; ApoE-CH_3_Hg system = interaction energy value of the ApoE-CH_3_Hg complexes; interaction energy = difference between the total value of the system and the sum of the isolated components.

The ApoE3-MeHg interactions, represented in Figure 1A, show the mercury atom coordinated mainly by S and O atoms, in addition to C and H. In Figure 1B, the BCPs confirm the shared electron density. The values presented in Table 2 indicate that interactions with sulfur are more intense, as indicated by ρ and ELF; the nature of the interaction is confirmed by (∇^2^ρ > 0). For sulfur, ρ172 = 0.0542 and ρ143 = 0.0330 were obtained, in addition to ELF172 = 0.2901 and ELF143 = 0.1375. The interaction with oxygen presented ρ160 = 0.0339 and ELF160 = 0.0687. Although ρ and ELF for CP160 do not follow the same trend, it is noteworthy that sulfur, with two BCPs, presents a more expressive total electron density.

**Table 2:** ρ, ELF (Electronic Location Function), and ∇^2^ρ Values of the ApoE2 Structures at Cysteine 112 and Cysteine 158, ApoE3, and ApoE4 with CH^3^Hg

**structure: ApoE^3^-CH^3^Hg**			
**BCPs**	**ρ/a.u.**	**ELF**	**∇** ^**2**^ **ρ/a.u.**
172	0.0542	0.2901	0.1028
143	0.0330	0.1375	0.0906
160	0.0339	0.0687	0.1619
166	0.1156	0.4591	0.1522

**structure: ApoE4—CH^3^Hg**			
**BCPs**	**ρ/a.u.**	**ELF**	**∇** ^**2**^ **ρ/a.u.**
229	0.0032	0.0221	0.0066
207	0.0117	0.0786	0.0252
168	0.0093	0.0346	0.0293
162	0.0301	0.1301	0.0849
190	0.0624	0.2158	0.1605
160	0.0361	0.1256	0.1075
199	0.0950	0.3901	0.1529

**structure: ApoE2-cys112—CH^3^Hg**			
**BCPs**	**ρ/a.u.**	**ELF**	**∇** ^**2**^ **ρ/a.u.**
184	0.0585	0.3085	0.1079
199	0.0405	0.1674	0.1071
220	0.0033	0.0114	0.0100
186	0.1140	0.4750	0.1386

**structure: ApoE2-cys112—CH^3^Hg**			
**BCPs**	**ρ/a.u.**	**ELF**	**∇** ^**2**^ **ρ/a.u.**
150	0.0057	0.0217	0.0172
157	0.0809	0.3858	0.1308
175	0.0037	0.0090	0.0126
178	0.0937	0.5326	0.0812

**Figure 1:**
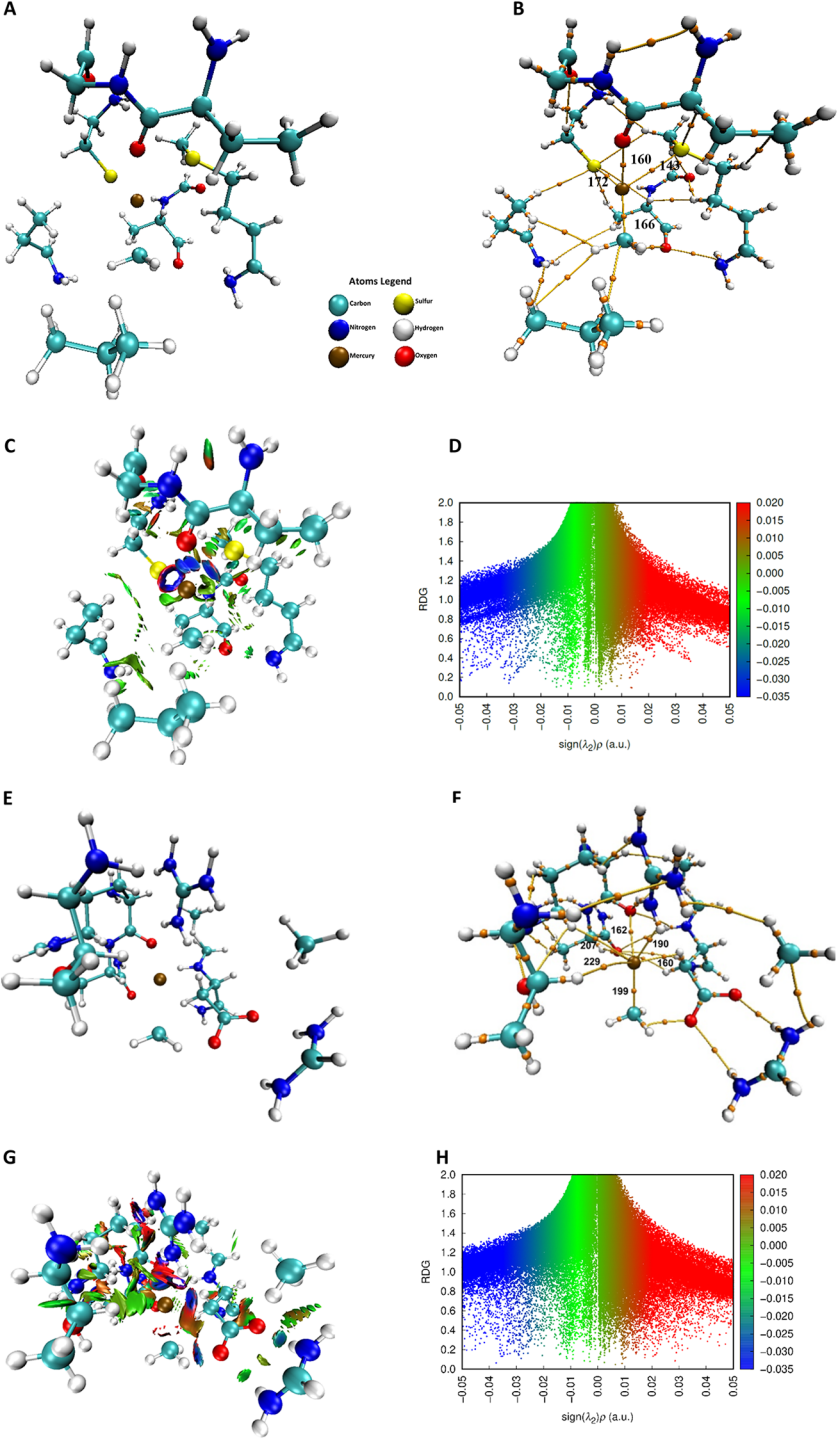
6 Å cross section of the ApoE3 and ApoE4 structures with MeHg. (A) Geometric structure of the cross-section of ApoE3 and MeHg. (B) Binding pathways and binding critical points (BCPs) obtained by QTAIM calculations with identification of the BCPs around Hg in the ApoE3-MeHg geometry. (C) Isosurfaces obtained by NCI calculations for the ApoE3-MeHg interaction. (D) RDG indicates the strong, weak, and repulsive attractive interactions, complementing the isosurfaces. (E) Geometric structure of the cross-section of ApoE4 and MeHg. (F) Binding pathways and binding critical points (BCPs) obtained by QTAIM calculations with identification of the BCPs around Hg in the ApoE4- MeHg geometry. (G) Isosurfaces obtained by NCI calculations for the ApoE4-MeHg interaction. (H) RDG indicates the strong, weak, and repulsive attractive interactions, complementing the isosurfaces.

Figure 1C,D qualitatively show the Hg atom surrounded by a blue isosurface, predominant in the direction of the S and O atoms. Other green isosurfaces indicate weaker van der Waals interactions. The RDG map (Figure 1D) displays blue peaks between sing­(λ2)­ρ −0.03, −0.04, and −0.05, characterizing strong interactions, and green peaks of sing­(λ2)­ρ between 0 and −0.02, denser, which reflect the total geometry.

Figure 1E shows the geometry of ApoE4-MeHg, showing the mercury atom coordinated mainly by O and H atoms. Figure 1F highlights the 6 BCPs found around the Hg atom and the ApoE4 residues, confirming the shared electron density, with BCP199 referring to the MeHg binding. The values presented in Table 2 indicate that the interactions related to BCP190 and BCP162 with values ρ = 0.0624, ρ = 0.0624, and ELF = 0.2158 and ELF = 0.1301, respectively. Although the oxygen atom in BCP190 is more electronegative, BCP 162 has a higher electron density. This occurs because it is from a fragment that has 3 nitrogen atoms, which makes the electronic inductive effect prevalent. Furthermore, these interactions form a cyclic region of electron density that can be seen in Figure 1G with the isosurfaces around the Hg atom. The region corresponding to these BCPs has an intense blue coloration. The RDG map in Figure 1H has a blue peak in the sing­(λ2)­ρ region between −0.02 and −0.05.

Figure 2A shows the geometry of ApoE2 with cysteine at position 112 (ApoE2-Cys112) and MeHg interacting at this site of the respective ApoE. The obtained geometry shows the mercury atom coordinated mainly by S and H atoms, with the bonding paths demonstrating the interaction (Figure 2B). The three BCPs found around the Hg atom and the residues of ApoE2-Cys112 confirm the shared electron density. The BCP values are easier to evaluate because the most prominent values corresponding to ρ and ELF are justified by the high electron density of the S atom, and the fact that these atoms are bonded to groups with atoms of high electron density, contributing to inductive effects. Figure 2 **C–D**, with the isosurfaces around the Hg atom, highlights in blue the direction of interactions with the sulfur atoms and in green the interactions of the other fragments surrounding the Hg. Since it is not possible to observe many strong interactions in this fragment, Figure 2D does not show a directed peak, only a dispersion in the area corresponding to these interactions. On the other hand, the interactions in green, with sing­(λ2)­ρ between 0 and −0.01, have high density and good definition.

**Figure 2:**
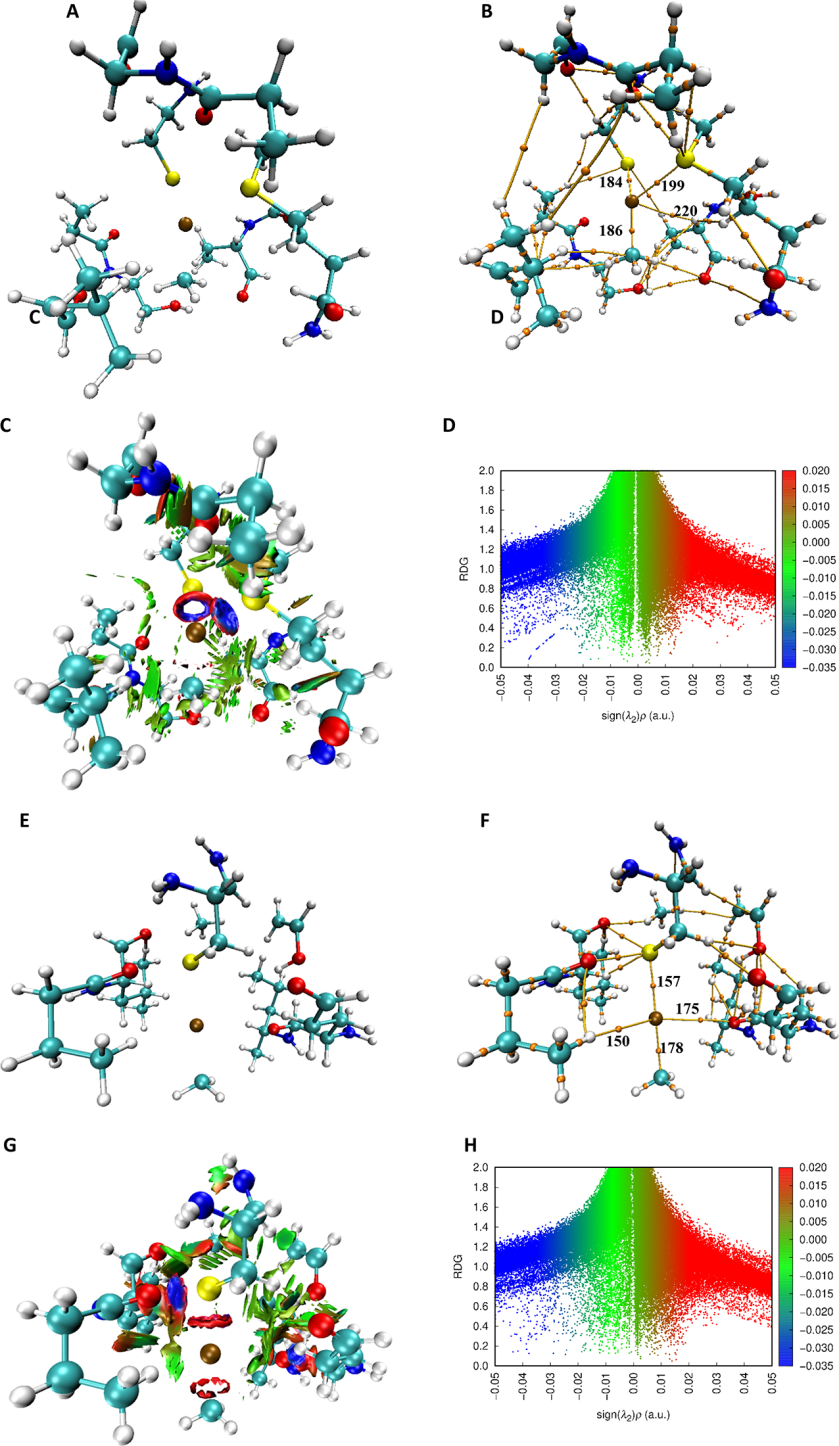
6 Å cross section of the ApoE2 structure with MeHg. (A) Geometric structure of the ApoE2 cross-section with cysteine 112 and MeHg. (B) Binding pathways and binding critical points (BCPs) obtained by QTAIM calculations with identification of BCPs around Hg in the ApoE2-Cys112-MeHg geometry. (C) Isosurfaces obtained by NCI calculations for the interaction of the ApoE2-Cys112-MeHg geometry. (D) RDG indicates the strong, weak, and repulsive attractive interactions, complementing the isosurfaces. (E) Geometric structure of the cross-section of ApoE2 with cysteine 158 and MeHg. (F) Binding pathways and binding critical points (BCPs) obtained by QTAIM calculations with identification of the BCPs around Hg in the ApoE2-Cys158-MeHg geometry. (G) Isosurfaces obtained by NCI calculations for the ApoE2-Cys158-MeHg geometry interaction. (H) RDG indicates the strong, weak, and repulsive attractive interactions, complementing the isosurfaces.

Figure 2E shows the geometry of ApoE2 with cysteine at position 158 (ApoE2-Cys158). The mercury atom is coordinated mainly by S, O, and H atoms, with three bonding paths between Hg and these atoms. It is also possible to observe some electron density sharing structures, as in Figure 2F, where the bonding paths of BCPs157 and 150 form a four-membered cyclic structure. The electron density of this structure can be seen in Figure 2G with the isosurface corresponding to this region, ranging from light blue to green to red. This variation causes the RDG plot in Figure 2H to have a profile in which the strong interactions do not present a defined peak, but rather a more dispersed area at the values of sing­(λ2)­ρ < 0, with some discrete indications at the values of −0.04 and between the values of −0.02 and −0.03. On the other hand, in red in the RDG, a peak is notable with a sing­(λ2)­ρ value between 0.01 and 0.02, which indicates only specific regions and despite being repulsive interactions, it is of low intensity. These computational findings provide structural insights that support the differential reactivity of MeHg with ApoE isoforms observed in vivo.

### 3.2 Hg Concentration in Hair, Liver, and EWF Following Chronic MeHg Intoxication

As expected, orally intoxicated wild-type and ApoE ko mice showed significantly higher Hg levels than the respective control groups in all tested samples (*p* < 0.001). Interestingly, intoxicated wild-type mice exhibited significantly lower Hg deposits in the hair than intoxicated mice lacking ApoE, *p* < 0.001 (Figure 3A). Intriguingly, Hg levels were higher in the liver and EWF samples in wild-type MeHg-exposed mice than their ApoE ko counterparts (*p* < 0.05) (Figures 3 **B, C**).

**Figure 3:**
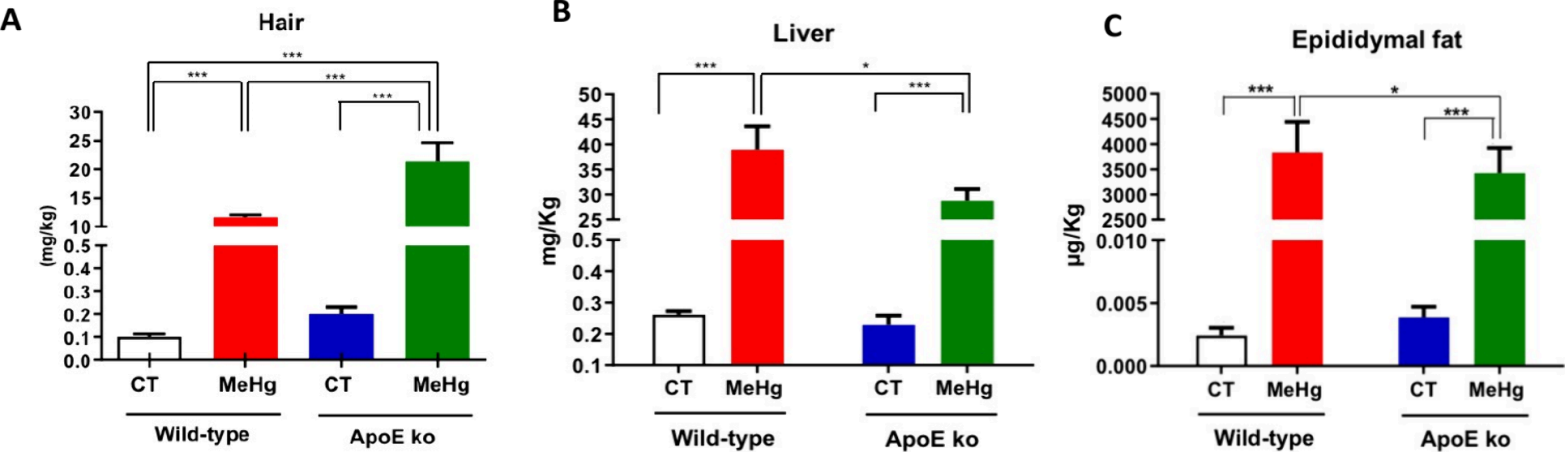
Effect of oral administration of MeHg (20 mg/L) for 20 days on MeHg concentration in hair (A), liver (B), and epididymal adipose tissue (C) of young ApoE ko and wild-type (WT) mice. The experimental groups consisted of WT control (CT), WT + MeHg, ApoE ko CT, and ApoE ko + MeHg. MeHg concentrations are expressed as mg/kg in hair and liver, and μg/kg in epididymal adipose tissue. Data are presented as mean ± SEM. The number of animals per group was at least *n* = 10. *p < 0.05 and **p < 0.001 between groups by two-way analysis of variance (ANOVA).

### 3.3 Body Weight Gain and Blood Lipids

MeHg chronic intoxication induced significantly lower weight gain in wild-type but not among ApoE ko mice, *p* < 0.01. Indeed, MeHg-intoxicated ApoE ko mice showed significantly greater weight gain than intoxicated wild-type counterparts, *p* < 0.01. No statistical significance was found between ApoE ko control versus intoxicated ApoE ko mice, suggesting that ApoE deficiency is the major driver of body weight gain. MeHg chronic exposure was not able to affect weight gain regardless of genetic background (Figure 4A,B). MeHg intoxication did not affect plasma triglyceride levels in both wild-type and ApoE ko mice compared to nonintoxicated controls. However, our findings indicate that the lack of ApoE was the major driver of the rise in triglyceride levels, regardless of MeHg intoxication (*p* < 0.01) (Figure 4C).

**Figure 4:**
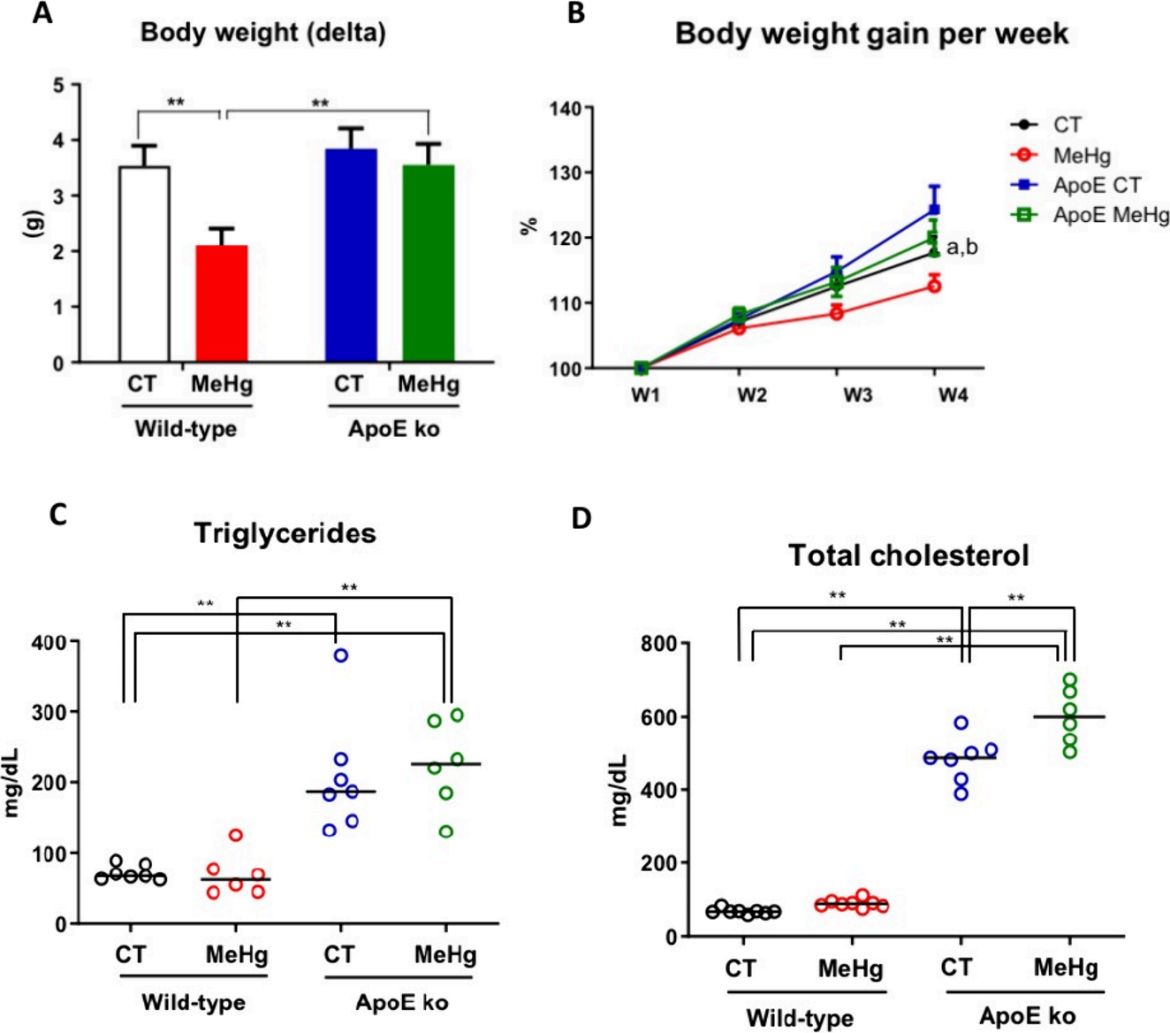
Effect of oral administration of MeHg (20 mg/L) for 20 days on body weight variation and lipid profile of young WT and ApoE KO mice. (A) Body weight change (delta, g); (B) weekly body weight gain expressed as percentage of baseline weight; (C) plasma triglyceride levels; and (D) plasma total cholesterol levels. The experimental groups consisted of wild-type controls (CT), MeHg-intoxicated mice (MeHg), ApoE knockout controls (ApoE ko CT), and MeHg-intoxicated ApoE knockout mice (ApoE ko MeHg). Data are presented as mean ± SEM. The number of animals per group was at least *n* = 6. **p < 0.001 between groups by two-way analysis of variance (ANOVA).

We did not detect significant differences in plasma total cholesterol levels between the control and intoxicated wild-type mice. The ApoE deficiency also significantly increases total cholesterol levels independent of MeHg intoxication (*p* < 0.01). Conversely, ApoE ko mice showed the highest total cholesterol levels following MeHg intoxication (Figure 4D).

### 3.4 Liver Enzymes

MeHg intoxication did not affect plasma AST activity in both wild-type and ApoE ko mice compared to nonintoxicated controls. However, our findings indicate that the lack of ApoE was the major driver of the rise in AST activity, regardless of MeHg intoxication (*p* < 0.01) (Figure 5A).

**Figure 5:**
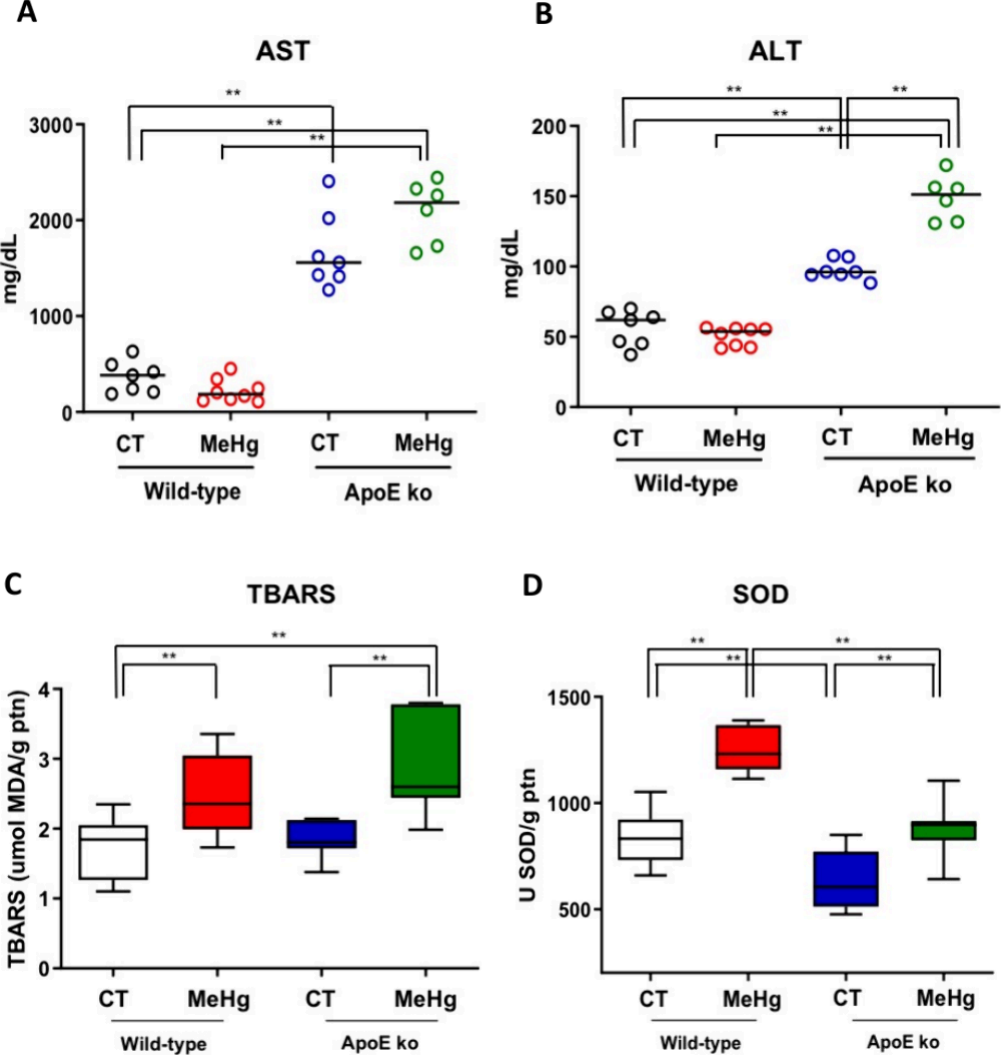
Effect of oral administration of MeHg (20 mg/L) for 20 days on hepatic injury markers and oxidative stress parameters in young WT and ApoE KO mice. (A) Plasma aspartate aminotransferase (AST) activity; (B) plasma alanine aminotransferase (ALT) activity; (C) thiobarbituric acid reactive substances (TBARS) levels in liver tissue; and (D) superoxide dismutase (SOD) activity in liver tissue. The experimental groups consisted of wild-type controls (CT), MeHg-intoxicated mice (MeHg), ApoE knockout controls (ApoE ko CT), and MeHg-intoxicated ApoE knockout mice (ApoE ko MeHg). Data are presented as mean ± SEM *N* = 6 mice per experimental group. **p < 0.001 between groups. by two-way analysis of variance (ANOVA).

We did not detect significant differences in plasma ALT activity between the control and intoxicated wild-type mice. The ApoE deficiency also led to a significant increase in ALT activity independent of MeHg intoxication (*p* < 0.01). However, ApoE ko mice showed the highest ALT activity following MeHg intoxication (Figure 5B).

### 3.5 Oxidative Stress in Liver Tissue

MeHg intoxication has led to significantly higher TBARS and SOD activity in the liver when compared to their respective nonintoxicated controls, regardless of genetic background (*p* < 0.01). We found an increase in oxidative stress, as indicated by TBARS levels in ApoE ko mice intoxicated with MeHg compared to wild-type controls (*p* < 0.01), suggesting a compound effect of these two conditions (Figure 5C).

ApoE ko intoxicated animals presented lower activity of SOD when compared to wild-type intoxicated animals (*p* < 0.01), indicating that the absence of ApoE may have contributed to this difference in the presence of MeHg (Figure 5D).

### 3.6 Hepatic Steatosis

Hepatic steatosis was not found in wild-type mice regardless of MeHg intoxication. ApoE deficiency alone was not sufficient to induce significant alterations in hepatic steatosis. However, MeHg intoxication (20 mg/L in drinking water) for 20 days caused a significant increase in hepatic steatosis scores in ApoE-deficient mice when compared to ApoE nonintoxicated controls or wild-type control mice (*p* < 0.05). Regarding the cell ballooning score, statistical differences were only detected after comparing wild-type controls versus ApoE ko intoxicated groups (Table 3).

**Table 3:** Effects of Mercury Intoxication (20 mg/L in Drinking Water) on Histopathological Scores in the Liver of Wild-Type C57BL6/J and ApoE ko Mice^d^

groups	steatosis	ballooning
Control	0 (0–0)	0 (0–1)
MeHg	0 (0–0)	0 (0–2)
ApoE Ko CT	0 (0–1)	1 (0–2)
ApoE Ko MeHg	0 (0–1)^a^ ^,^ ^b^	1 (0–2)^c^

a *p* < 0.05 compared to wild-type controls by the Kruskal–Wallis test.

b *p* < 0.05 compared to ApoE Ko by the Kruskal–Wallis test.

c *p* < 0.05 compared to wild-type controls by the Mann–Whitney test.

d *N* = 10–12 mice per group. CT = unintoxicated mice.

### 3.7 Plasma Leptin Levels

Nonintoxicated wild-type mice showed significantly higher plasma leptin levels when compared to MeHg-intoxicated and nonintoxicated ApoE ko mice. No statistical difference was observed between wild-type controls versus MeHg-exposed wild-type mice and ApoE ko control vs ApoE ko intoxicated groups (Figure 6B).

**Figure 6:**
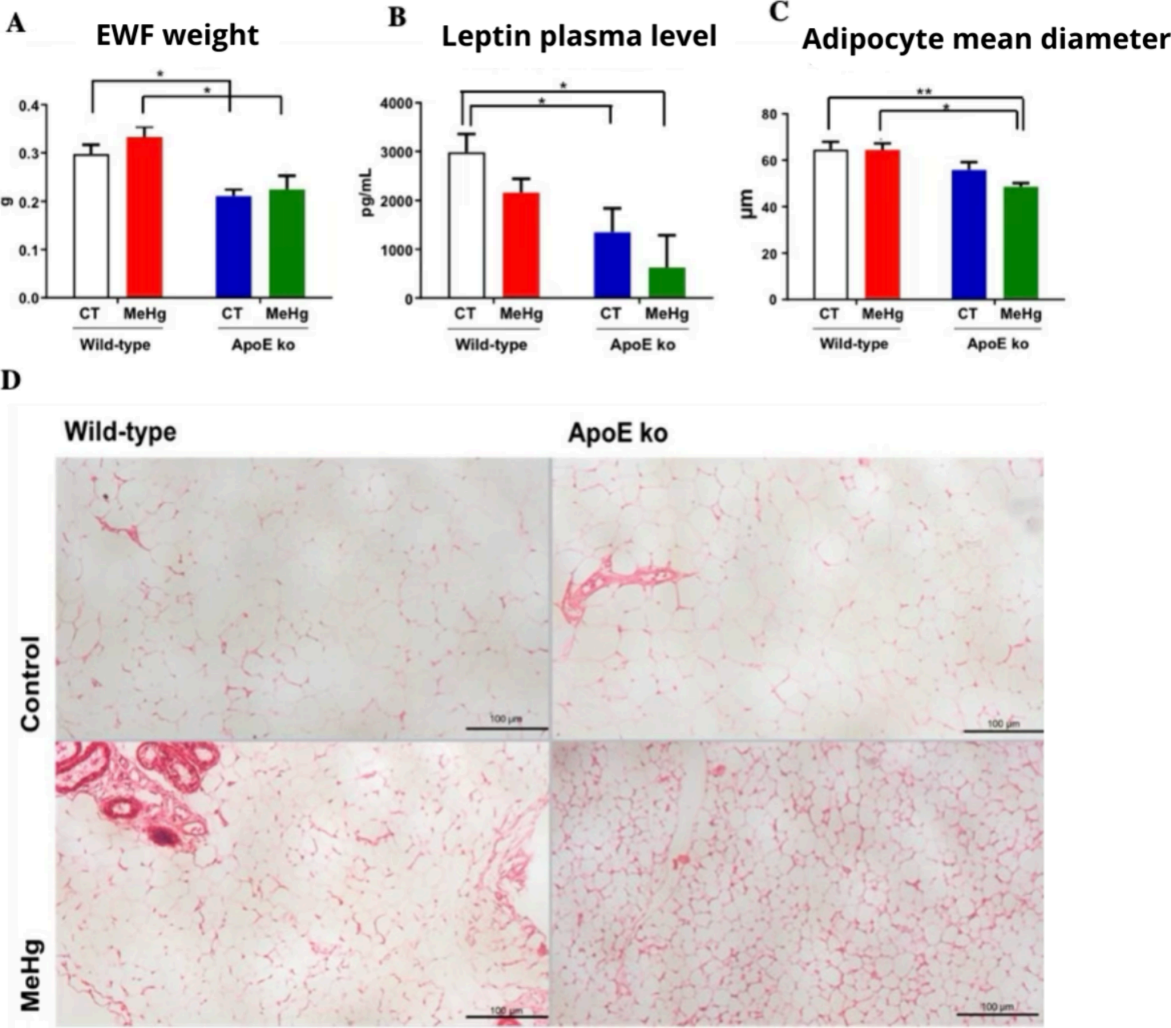
Effect of oral administration of MeHg (20 mg/L) for 20 days on epididymal white fat (EWF) tissue parameters in adult WT and ApoE ko mice. (A) EWF weight; (B) plasma leptin concentration; (C) mean adipocyte diameter; and (D) representative histological sections of EWF. The experimental groups consisted of wild-type controls (CT), MeHg-intoxicated mice (MeHg), ApoE knockout controls (ApoE ko CT), and MeHg-intoxicated ApoE knockout mice (ApoE ko MeHg). Plasma leptin concentrations are expressed as pg/mL, adipocyte diameter as μm, and EWF weight as grams (g). Data are presented as mean ± SEM *N* = 6 mice per experimental group. *p < 0.05 and **p < 0.01 by two-way analysis of variance (ANOVA). Histological images were obtained from EWF sections stained with hematoxylin and eosin (H&E); scale bar = 100 μm.

### 3.8 Weight of EWF and Morphometry of EWF Adipocytes

MeHg intoxication did not significantly affect EWF weight in wild-type mice. Interestingly, ApoE ko mice showed reduced EWF weight than the wild-type mice, regardless of experimentally MeHg exposure (*p* < 0.05). Among ApoE ko mice, no significant differences were observed between controls compared with intoxicated mice, indicating a stronger effect of ApoE deficiency than MeHg intoxication (Figure 6A)

Among intoxicated mice, ApoE deficiency was sufficient to reduce the diameter of adipocytes (*p* < 0.05). Intoxicated ApoE ko mice showed the greatest reduction in adipocyte diameter (*p* < 0.01). MeHg intoxication did not cause significant reductions in adipocyte diameter among wild-type mice compared to their respective controls (Figure 6C,D).

### 3.9 EWF Metabolomics Analysis

Evaluating all biological scenarios, the multivariate statistics display a good discriminant separation (Q2 = 0.66) in the partial least-squares-discriminant analysis (PLS-DA), with a clearer separation from the APOE-ko mice (ApoE ko CT; ApoE ko MeHg) when compared to the wild-types (WT CT; WT MeHg) (Figure 7A). Variable importance in projection (VIP) scores revealed high score (≥2) for most of the metabolites that were able to discriminate each biological scenario (Figure 7B) and the heatmap shows how these scenarios are clustered related to their molecular features (MFs) intensity. In Figure 7C, a clear separation was found regarding the intensity of these MFs when compared ApoE-ko phenotypes (ApoE ko CT; ApoE ko MeHg) vs wild-type phenotypes (WT CT; WT MeHg). An interactome map of the VIP metabolites is displayed in Figure 7D, where most of the metabolites, such as glycerophosphocholine, taurine, and creatine, are in the main interactome backbone.

**Figure 7:**
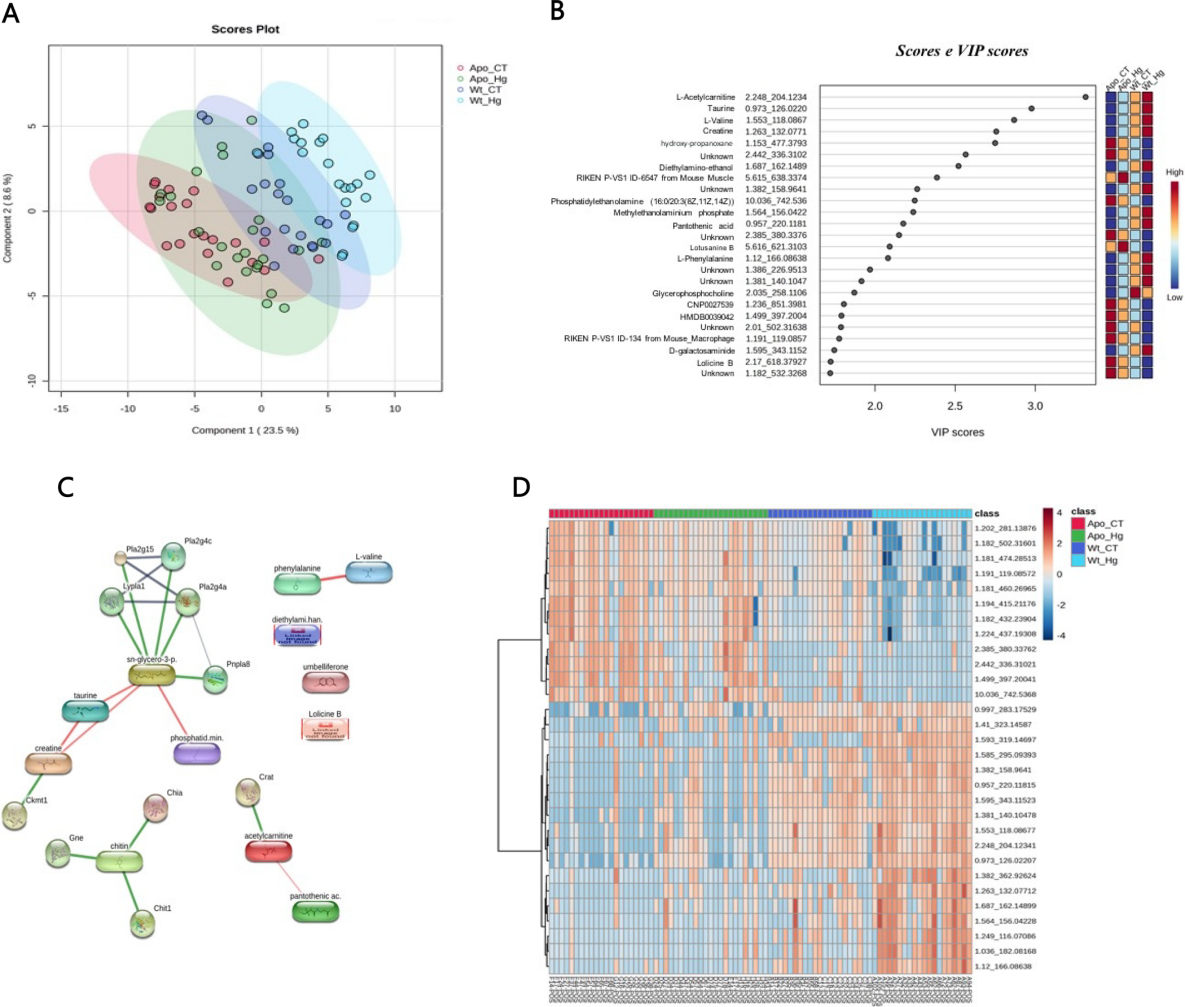
Multivariate and network-based analysis of metabolomics data across all experimental groups. The experimental groups consisted of wild-type controls (CT), MeHg-intoxicated mice (MeHg), ApoE knockout controls (ApoE ko CT), and MeHg-intoxicated ApoE knockout mice (ApoE ko MeHg). (A) Partial least-squares–discriminant analysis (PLS-DA) score plot showing group separation based on metabolomic profiles. (B) Variable importance in projection (VIP) scores derived from the PLS-DA model. Metabolite annotation includes metabolite name, experimental retention time, and experimental monoisotopic mass. (C) Heatmap showing the relative abundance of discriminant molecular features across samples, clustered by biological similarity. (D) Interactome analysis of VIP metabolites, illustrating functional associations between metabolites. Node size represents the number of connections (node size ≥ 2), and edges indicate interaction confidence (medium confidence = 0.400). Color scales indicate relative metabolite abundance, with warmer colors representing higher levels and cooler colors representing lower levels.

When we analyze the scenario ApoE-ko mice – ApoE ko CT vs ApoE ko MeHg, a low permutation value (p = 0.06) was seen in the orthogonal partial least-squares-discriminant analysis (oPLS-DA). The metabolites were able to discriminate the proposed model (Figure 8A). The metabolites L-acetylcarnitine, sphinganine, taurine, hexaethylene glycol, and diethanolamine were more intense in the ApoE ko MeHg scenario with VIP scores ≥ 2, therefore representing good discriminant features for this phenotype (Figure 8B). An interactome map of the VIP metabolites is displayed in Figure 7C. We can observe that most of the metabolites on the VIP score chart are interacting in the main backbone (Figure 8C).

**Figure 8:**
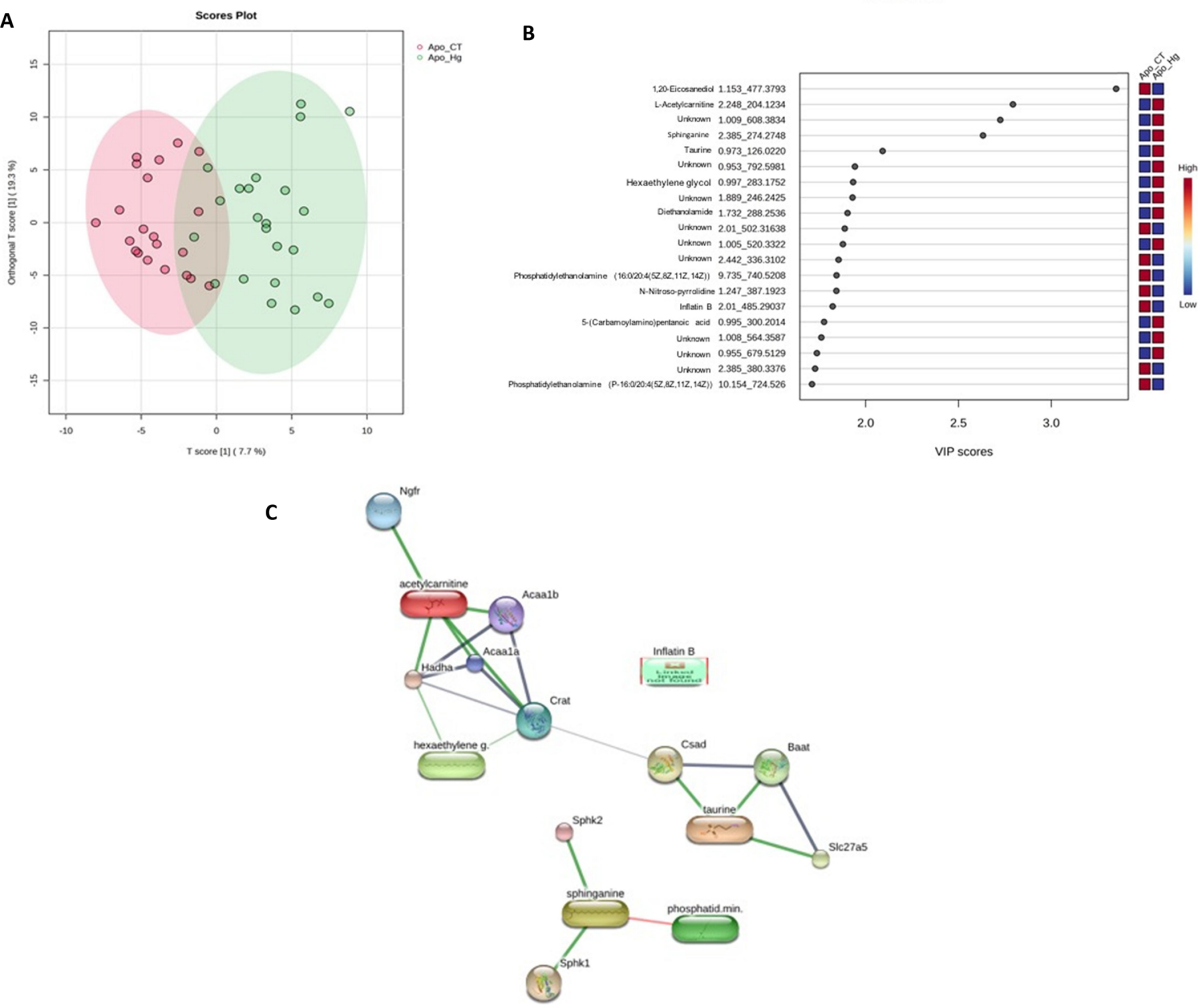
Metabolomics data analysis of ApoE ko mice exposed to MeHg. (A) Orthogonal partial least-squares–discriminant analysis (oPLS-DA) score plot of liver metabolomic profiles from ApoE ko mice in the ApoE ko CT and ApoE ko MeHg. Each point represents an individual animal. Shaded ellipses indicate the 95% confidence interval for each group. The percentage of variance explained by each component is shown on the axes. (B) Variable importance in projection (VIP) scores derived from the oPLS-DA model, highlighting metabolites contributing most to group discrimination. Metabolites are annotated by compound name, chromatographic retention time (min), and experimental monoisotopic mass (*m*/*z*). Higher VIP scores indicate greater contribution to the separation between ApoE ko CT and ApoE ko MeHg groups. (C) Protein–metabolite interaction network (interactome) constructed from VIP metabolites. Nodes represent metabolites or associated proteins, and edges represent predicted interactions with medium confidence (confidence score ≥ 0.400). Node size reflects interaction degree (node size ≥ 2). Multivariate statistical analysis was performed using oPLS-DA, with model validation conducted according to standard metabolomics criteria. A VIP score >1.0 was considered indicative of relevant discriminatory metabolites.

On the biological scenario related to the wild-type mice – WT CT vs WT MeHg, we observe a low permutation value (*p* < 0.01) in the orthogonal partial least-squares-discriminant analysis (oPLS-DA). The metabolites were able to discriminate the proposed model (Figure 9A). The metabolites phosethanolamine, creatine, and diethylethanolamine have VIP scores higher than three and are highly discriminant against the WT MeHg scenario. Also, the metabolites l-tyrosine, l-phenylalanine, and 1–4 oxazepane have high and discriminant VIP scores for the WT MeHg scenario, as shown in Figure 9B. An interactome map of the VIP metabolites is displayed in Figure 9C, and we can observe that the amino acids interacting in the same node (Figure 9C). A supplementary table (Table S1) lists mass-to-charge ratio (*m*/*z*), retention time, adduct, tandem mass spectrometry (MS/MS) availability, match score, and identification level of detected metabolites.

**Figure 9:**
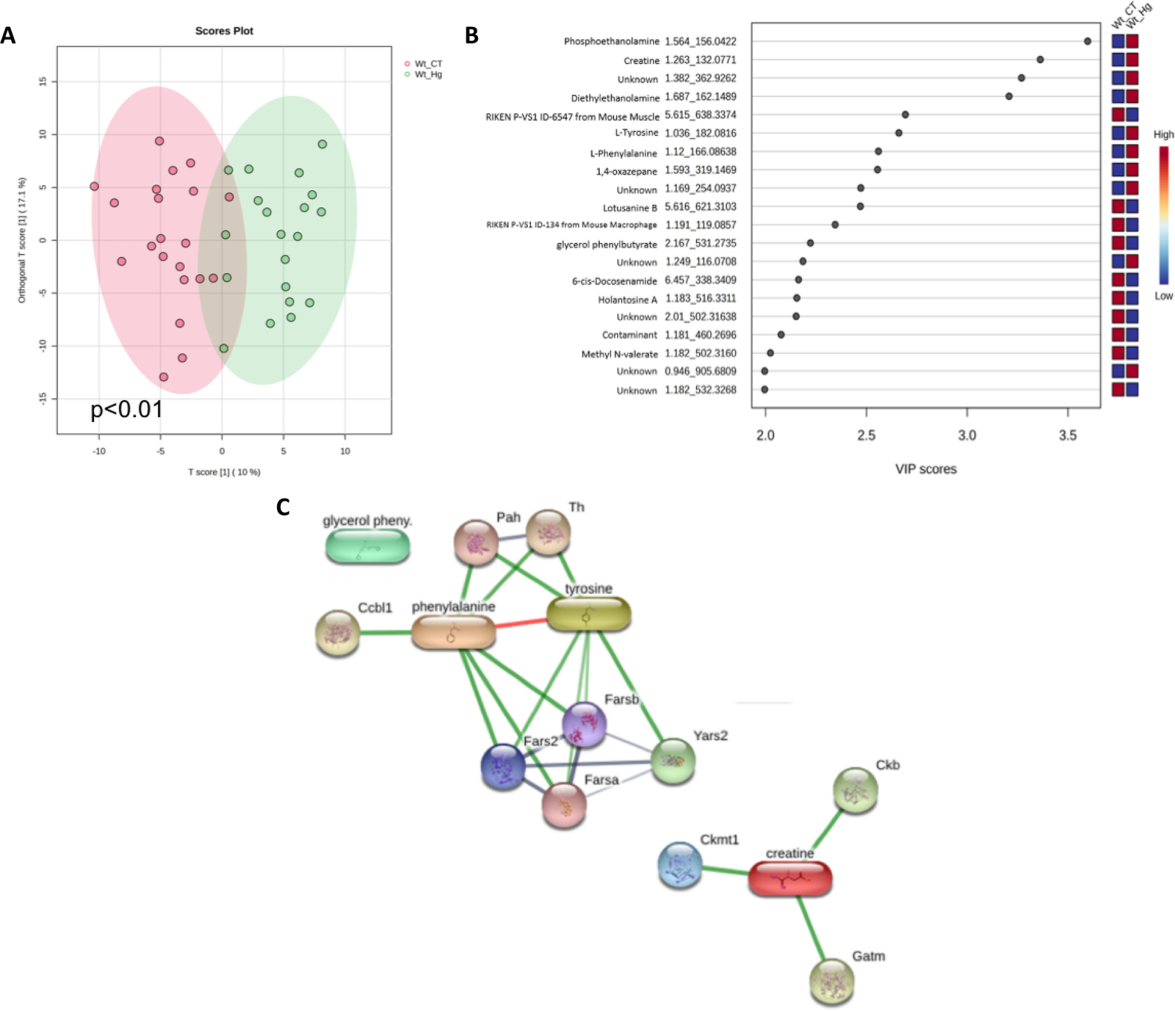
Metabolomics data analysis of wild-type mice exposed to MeHg. (A) Orthogonal partial least-squares–discriminant analysis (oPLS-DA) score plot of liver metabolomic profiles from WT mice in the WT CT and WT MeHg. Each point represents an individual animal. Shaded ellipses indicate the 95% confidence interval for each group. The percentage of variance explained by each component is shown on the axes. Group separation was statistically significant (*p* < 0.01). (B) Variable importance in projection (VIP) scores derived from the oPLS-DA model, highlighting metabolites that contribute most to the discrimination between WT CT and WT MeHg groups. Metabolites are annotated by compound name, chromatographic retention time (min), and experimental monoisotopic mass (*m*/*z*). Higher VIP scores indicate a greater contribution to group separation. (C) Protein–metabolite interaction network (interactome) constructed from VIP metabolites. Nodes represent metabolites or associated proteins, and edges represent predicted interactions with medium confidence (confidence score ≥ 0.400). Node size reflects the interaction degree (node size ≥ 2). Multivariate statistical analysis was performed using oPLS-DA, and a VIP score >1.0 was considered indicative of relevant discriminatory metabolites.

## 4 Discussion

Computational data provides a molecular perspective that complements the results obtained in ApoE ko animals. Although the in vivo model represents a condition of complete ApoE deficiency, the in-silico findings with human isoforms help elucidate how the absence or alteration of specific residues modulates mercury-binding capacity.

Semiempirical calculations revealed that ApoE2 and ApoE3 establish strong Hg–S interaction bonds with MeHg, resulting in energetically stable complexes. Conversely, ApoE4, lacking these thiol residues, exhibited positive interaction enthalpies and no stable MeHg docking. This loss of binding capacity in ApoE4 reflects, at the molecular level, the lack of ApoE-mediated protection observed in the knockout model, leading to increased MeHg bioavailability and greater susceptibility to oxidative and metabolic disorders.

Our findings depict a tendency for Hg–S interactions to form in the Cys112, ApoE3, and Cys158 systems. These interactions are characterized by high electron density sharing, which is corroborated by the electron density (ρ) and Electron Localization Function (ELF) values at the Binding Critical Points (BCPs). RDG and NCI analyses further confirmed the presence of attractive regions located near sulfur atoms only in ApoE2 and ApoE3. These structural and energetic differences support the hypothesis that the absence of ApoE expression, as observed in the knockout model, results in a complete loss of mercury-binding capacity, similar to the reduced affinity predicted for the ApoE4 isoform.

In this condition, MeHg remains more available to interact with other biomolecules, such as neuronal and mitochondrial proteins, promoting oxidative stress, mitochondrial dysfunction, neuroinflammation, and alterations in lipid metabolism. These mechanisms have already been described in association with metal toxicity^7,34^ and are consistent with the metabolic and inflammatory disturbances observed in ApoE-deficient tissues in vivo.

To the best of our knowledge, this is the first study to investigate the impact of MeHg intoxication on the function and structure of a metabolically active EWF in ApoE-deficient animals. The study aimed to investigate how MeHg and ApoE deficiency, alone or in combination, affect EWF and liver tissues and assess whether ApoE deficiency and MeHg intoxication exhibit compounded deleterious effects. ApoE ko mice are well-recognized to exhibit spontaneous dyslipidemia, even at an early stage of life^15^

As expected, MeHg-exposed mice had significantly higher concentrations of Hg in the fur, epididymal adipose tissue, and liver than the nonexposed groups, confirming the disseminated toxicity of the experimental model. Of note, Rizzetti et al. could not find higher significant Hg deposition in the epididymal adipose tissue from exposed mice (challenged by 20 mg/L of mercury chloride (HgCl^2^) in drinking water) compared to unchallenged mice.^35^ This difference is likely due to MeHg’s greater capacity to overcome cellular barriers compared to elemental Hg^7^

Intriguingly, ApoE ko mice display higher mercury levels in hair but lower levels in liver and epididymal white fat compared with wild-type mice. These possible differences may be due to distinct mercury transport, distribution, or clearance in the absence of ApoE. One possible explanation may be unique differences in the intestinal microbiota composition of these mice,^36^ as some bacterial taxa may affect MeHg bioavailability by promoting MeHg demethylation.^37,38^ The source of MeHg intoxication was oral intake, which would have reached the intestinal tract and was amenable to intestinal bacterial metabolism. We cannot rule out that the ApoE deficiency may affect the MeHg binding to the fat depots in the liver and the epididymal white fat. More studies are needed to dissect the mechanisms underlying MeHg bioavailability in dyslipidemic ApoE ko mice.

Although we could find reduced weight gain in intoxicated C57BL/6J mice when compared to their nonintoxicated controls, this is not a universal finding in MeHg intoxication studies. Ferrer and colleagues found that when 8-week-old C57BL/6J mice were chronically exposed to lower doses of MeHg (0.5 or 5 mg/L in drinking water), they did not show changes in body weight. However, they reported that it did affect the expression of hypothalamic neuropeptides (which participate in food intake and body weight control) and increased the anorectic neuropeptide pro-opiomelanocortin (POMC) in a dose-dependent manner.^39^

Increased adipogenesis can be seen in chronic and low-dose exposures, and inhibition of adipose tissue differentiation is found at higher doses,^40^ which may partly explain our findings. In our study, intoxication with MeHg did not alter plasma triglyceride levels in wild-type and ApoE ko mice compared to their nonintoxicated controls. Nonetheless, triglyceride levels were higher in ApoE ko mice regardless of MeHg exposure. Roque and colleagues found that 20 mg/L of MeHg in drinking water induced hypertriglyceridemia in wild-type mice. This MeHg effect was augmented by ApoE deficiency.^15^

Additionally, ApoE ko mice displayed significantly greater levels of cholesterol when compared to wild-type controls, and MeHg-challenged ApoE ko mice showed even more pronounced hypercholesterolemia. From these findings, long-term MeHg exposure may be more harmful to dyslipidemic individuals. Mounting evidence indicates worsening cardiovascular risk and accelerated atherosclerosis in ApoE ko mice. Chronic MeHg intoxication may raise total blood cholesterol due to increased atherogenic or non-HDL fraction.^41^ MeHg seems to bind to sulfhydryl groups in the main apoprotein of the non-HDL fraction (apoB-100), which can induce conformational rearrangements that compromise binding with its receptors.^42^ Reduced LDL intake would contribute to a further rise in serum total cholesterol levels.^41^

AST and ALT blood activities were not significantly altered by MeHg exposure, which may not rule out subtle hepatotoxicity or later effects outside the study’s time frame. Although another study showed that HgCl_2_-treated mice did not show changes in liver aminotransferase activities.^43^ Yet, ApoE ko mice showed increased blood ALT and AST activities compared to nonintoxicated wild-type mice, with amplifying effects following MeHg intoxication, suggesting that dyslipidemic (increased ALT, but not AST) mice are vulnerable to liver damage by MeHg. Hypercholesterolemic ApoE ko mice are at a higher probability of developing severe liver injury later, suggesting that increased oxidative stress and cholesterol products may act as a catalyst in the process of liver aging.^44^ In our model, increased liver oxidative stress (liver TBARS levels) with MeHg intoxication (regardless of ApoE background) may indicate an early subclinical liver injury that may precede a more significant liver damage. It is important to highlight the compounded effect of ApoE deficiency and MeHg intoxication on liver injury with increased blood ALT levels. ALT is considered a better biomarker of liver injury than AST^45^

Our findings did not show significant MeHg-induced liver steatosis in wild-type mice; however, MeHg induced hepatic steatosis in ApoE-deficient mice, suggesting that a prior dyslipidemic state could predispose to this alteration. Nonalcoholic steatosis in the liver with increased Kupffer cell counts was documented elsewhere after exposing mice to a higher dose of MeHg (20 mg/L in the drinking water) for 2 weeks in wild-type mice.^46^ This later finding reinforces the need for a more robust intoxication to trigger a significant liver pathology. Notably, Wistar rats treated with 5 mg/kg/day of MeHg and 1 mg/kg/day of diphenyl diselenide, administered intragastrically for 21 days, exhibited increased Hg accumulation in the liver and brain, resulting in motor deficits and weight loss.^47^

In our study, liver TBARS levels were augmented as a proxy of oxidative stress due to MeHg intoxication, corroborating a strong body of evidence. Although ApoE deficiency showed higher TBARS levels than MeHg-exposed wild-type mice, this difference did not reach a statistically significant level. Our data also showed increased liver SOD activity; this effect may be partly explained by a compensatory mechanism that counterbalances the increased oxidative stress to better neutralize excess free radicals.^48^

Reduced absolute and relative weight of EWT and adipocyte size were seen with more prolonged mercury (HgCl^2^) intoxication and at low doses. Additionally, HgCl^2^ has been recognized as a potent environmental disruptor of white adipose tissue, reducing the mean adipocyte size and affecting adipogenesis, adipokine synthesis, and secretion.^35^ We have documented a synergistic effect of ApoE deficiency and MeHg intoxication, resulting in a reduction in adipocyte mean diameter in EWF.

Furthermore, mice fed a high-fat diet and injected subcutaneously with HgCl^2^ (1.0 mg/kg) showed decreased adipocyte size in males.^49^ MeHg (5 mg/L) for 30 days was able to induce anorexia in C57BL/6J wild-type mice. MeHg intoxication has been linked to an altered leptin-induced Janus kinase 2 (JAK2)/signal transducer and activator of transcription 3 (STAT3) signaling pathway in the hypothalamus.^39^ Interestingly, HgCl^2^ treatment significantly decreased serum leptin levels, accompanied by downregulation of leptin mRNA expression in white adipose tissue.^49^ When adipose tissue is transplanted from ApoE ko mice into wild-type recipients after being fed a standard chow or high-fat diet for 8 to 10 weeks, transplanted ApoE ko adipocytes were found to be significantly smaller than transplanted wild-type adipocytes after receiving a standard chow diet while following a high-fat diet the size of transplanted wild-type adipocytes increased by 106 × 103 μm^3^ and the size of ApoE ko adipocytes increased by only 19 × 103 μm^3.^ ^50^

ApoE is highly expressed in adipocytes, which positively correlates with body fat mass. Conversely, ApoE deficiency in adipose cells compromises lipoprotein internalization and triglyceride accumulation in the tissue. ApoE-deficient lipoproteins cannot induce preadipocytes to form lipid-filled round adipocytes.^51^ Furthermore, when challenged with a high-fat and high-sucrose diet, lower adiposity and higher insulin sensitivity are observed in ApoE ko mice compared to ApoE-sufficient mice. The authors related the findings to reduced lipid transport to insulin-sensitive tissues, improving diet-induced obesity and insulin resistance in these animals.^52^ These findings corroborate our lower epididymal adipose tissue mass seen in the nonintoxicated ApoE ko mice.

In our study, we found increased activity of creatine, l-phenylalanine, and l-tyrosine in the EWF following MeHg intoxication. Creatine-dependent ADP/ATP substrate cycling has been identified in thermogenic beige adipocytes involving UCP-1.^53^ It would be interesting to know whether chronic MeHg exposure could augment the number of beige adipocytes in EWF. l-tyrosine serum levels are elevated after long-distance aerobic exercise, with increased glucose and lipid metabolism.^54^ A high fat-induced mouse model of metabolic dysfunction-associated liver steatosis revealed that l-phenylalanine and l-tyrosine levels in liver samples were reduced when compared to the control group. Yu and colleagues have shown that l-phenylalanine and l-tyrosine were negatively associated with the levels of liver ALT, AST, and epididymal fat but were positively associated with bacteria of the genus *Muribaculaceae* in metabolomic analysis of cecum samples.^55^

In our metabolomics studies, we found increased activity of L-acylcarnitine, sphingomyelin, taurine, and hexamethylene glycol in the EWF, under ApoE deficiency and MeHg intoxication. Interestingly, the l-carnitine and acylcarnitine pathways are involved in liver flavin-containing monooxygenase-catalyzed oxidation of trimethylamine (TMA), generating trimethylamine oxide (TMAO). Of note, the intestinal microbiota can synthesize TMA. TMAO is considered an atherosclerosis biomarker, as it may disrupt the reverse cholesterol transport, leading to an increased accumulation of foam cells in arterial plaques.^56^ Elevated blood concentrations of L-acylcarnitine are also indicative of fatty acid oxidation disorders.^57^

In addition, it has been recognized that sphingomyelin plasma levels correlate with coronary heart disease, even independently of cholesterol plasma levels, and the blockage of sphingomyelin activity could reduce atherosclerosis in ApoE ko mice.^58^ Conversely, taurine supplementation is associated with improved adipose tissue metabolism and lipolysis, and aging-related diseases,^59,60^ and hexamethylene glycol is considered a ligand of the Toll-like-9 receptor^61^ which may be compensatory mechanisms in the context of the systemic adipose function. The plausible interactions between paracrine and systemic EWF functions of these metabolites remain elusive.

Although our screening metabolomics analyses are extensive, we need to underscore that the data remain mostly descriptive and require further confirmation in future mechanistic studies to better address their toxicological relevance in clinical settings and human populations.

The elevated levels of MeHg in the fur of mice, compared with average human hair concentrations worldwide, are a limiting factor in our study. However, it is noticeable that severe anthropogenic-related environmental disasters have increased substantially,^3^ which can lead to alarming levels of Hg contamination in water reservoirs and impact adjacent communities.

We acknowledge that our preclinical findings are from high-dose MeHg exposure; therefore, they should be distinguished from environmentally relevant or chronic low-dose exposure, and caution should be taken when extrapolating our data to population settings. Our findings may be more relevant to high-dose MeHg poisoning associated with life-threatening environmental disasters or severe human intoxication accidents. In addition, we acknowledge that the ApoE ko mice, which show ApoE deficiency and associated dyslipidemia, although more phenotypically similar to ApoE4 carriage,^62^ do not fully represent human ApoE polymorphic features; hence, we cannot extrapolate the data to depict the human isoform-specific variation of this protein and its outcomes.

More studies are also needed to address the dynamic molecular interactions between ApoE isoforms and MeHg within a real, in vivo physiological environment to confirm the in silico findings, as our analyses were based on localized residues and a specific cutoff radius, which may not fully capture the impact of protein folding on mercury binding.

## 5 Conclusions

Our findings integrate in silico and in vivo evidence, expanding our understanding of how ApoE modulates susceptibility to MeHg toxicity. Combining experimental and computational approaches, this study demonstrates that the absence or structural impairment of ApoE increases vulnerability to MeHg-induced oxidative and metabolic dysfunction.

Altogether, our results suggest that ApoE deficiency and MeHg intoxication negatively affect the structure and function of white adipose tissue with liver toxicity. More studies are warranted to investigate the fine crosstalk signaling mechanisms underlying these modifications in ApoE ko mice. A better understanding of the negative interactions between dyslipidemia and MeHg intoxication is key for building efficacious nutritional interventions to mitigate any compound deleterious effects in highly exposed human populations.

## Supplementary Material



## Notes

(Table S1) Mass-to-charge ratio (*m*/*z*), retention time, adduct, tandem mass spectrometry (MS/MS) availability, match score, and identification level of detected metabolites (PDF)
