# Supplementary material for: In Silico ApoE Isoform Interactions with Methylmercury (MeHg) and In Vivo MeHg Intoxication Effects on Epididymal White Fat Tissue and Liver Function in Young ApoE Knockout Mice
Source: Chem Res Toxicol. 2026 May 5;39(5):862–78. doi: 10.1021/acs.chemrestox.5c00450 (PMC13188165; doi:10.1021/acs.chemrestox.5c00450)
Supplement: Supplementary file 1 [file tx5c00450_si_001.pdf]

Supporting Information

In silico ApoE isoforms interactions with methylmercury (MeHg) and in vivo MeHg intoxication effects on epididymal white fat tissue and liver function in young ApoE knockout mice

Synara C. Lopes[1], Vitória K. Felix Monteiro[1], Paola Caroline L. Leocádio[2], Marcus V. F. Rodrigues[3], Mirna Maciel d'Auriol Souza[2], Maria José N. de Paiva[2], Flávia Zandonadi[4], Alessandra Sussulini[4], Ramon Raposo[5], Francisco Leomar da Silva[6], Dávila Zampieri[6], Antonio Augusto Coppi[6], Aline M. A. Martins[7], Jacqueline Alvarez-Leite[2], Ámison R. Lopes da Silva[8], Norberto de K. V. Monteiro[3], Reinaldo B. Oriá[1]*

[1]Laboratory of the Biology of Tissue Healing and Nutrition, Department of Morphology and Institute of Biomedicine, School of Medicine, Federal University of Ceara, Fortaleza, CE 60430-270, Brazil
[2]Department of Biochemistry and Immunology, Federal University of Minas Gerais, Belo
Horizonte, MG 31270-901, Brazil
[3]Department of Analytical Chemistry and Physical Chemistry, Federal University of Ceara, Fortaleza, CE 60020-181, Brazil
[4]Department of Analytical Chemistry, Institute of Chemistry, State University of Campinas, Campinas, SP 13086-002, Brazil
[5]Experimental Biology Core, Health Sciences, University of Fortaleza, Fortaleza, CE 60811-905, Brazil
[6]University of Bristol Faculty of Health Sciences Bristol, England, UK BS8 1UD
[7]Integrated Space Stem Cell Orbital Research Center/Stanford Consortium for Regenerative Medicine, University of California, San Diego, La Jolla, CA 92093, USA
[8] Federal University of the São Francisco Valley, Senhor do Bonfim, BA 48970-000, Brazil
*Corresponding author. Email: oria@ufc.br

Table of Contents

Table S1. Mass-to-charge ratio (m/z), retention time, adduct, tandem mass spectrometry (MS/MS) availability, match score, and identification level of detected metabolites... S3

Table S1. Mass-to-charge ratio (m/z), retention time, adduct, tandem mass spectrometry (MS/MS) availability, match score, and identification level of detected metabolites.

File name	Title	MS1 count	MSMS coun	Precursor	Precursor t	Structure	Total score	Databases
ID00006_1	Unknown	0	462	1,140,916	[M+H]+	Epsilon-ca	5,955	HMDB=HM
ID00006_1	Unknown	0	462	1,140,916	[M+H]+	N,N-dimeth	5,692	ChEBI=CH
ID00006_1	Unknown	0	462	1,140,916	[M+H]+	1-isopropyl	5,615	ChEBI=CH
ID00006_1	Unknown	0	462	1,140,916	[M+H]+	2-Acetylpyr	55,391	HMDB=HM
ID00006_1	Unknown	0	462	1,140,916	[M+H]+	1-Piperidin	54,903	HMDB=HM
ID00006_1	Unknown	0	462	1,140,916	[M+H]+	2,5-Dihydr	52,393	HMDB=HM
ID00009_1	Unknown	0	161	1,161,437	[M+H]+	Isobutylpro	57,321	HMDB=HM
ID00009_1	Unknown	0	161	1,161,437	[M+H]+	diethyl(pro	57,119	ChEBI=CH
ID00009_1	Unknown	0	161	1,161,437	[M+H]+	2,4-dimeth	56,432	HMDB=HM
ID00009_1	Unknown	0	161	1,161,437	[M+H]+	methylhexa	54,716	ChEBI=CH
ID00009_1	Unknown	0	161	1,161,437	[M+H]+	tuaminohe	53,203	ChEBI=CH
ID00011_1	Unknown	0	144	1,161,437	[M+H]+	diethyl(pro	55,566	ChEBI=CH
ID00011_1	Unknown	0	144	1,161,437	[M+H]+	Isobutylpro	53,688	HMDB=HM
ID00011_1	Unknown	0	144	1,161,437	[M+H]+	2,4-dimeth	52,173	HMDB=HM
ID00011_1	Unknown	0	144	1,161,437	[M+H]+	tuaminohe	5,161	ChEBI=CH
ID00011_1	Unknown	0	144	1,161,437	[M+H]+	methylhexa	50,667	ChEBI=CH
ID00012_1	Unknown	0	139	1,161,438	[M+H]+	Isobutylpro	54,072	HMDB=HM
ID00012_1	Unknown	0	139	1,161,438	[M+H]+	2,4-dimeth	51,878	HMDB=HM
ID00012_1	Unknown	0	139	1,161,438	[M+H]+	diethyl(pro	51,845	ChEBI=CH
ID00012_1	Unknown	0	139	1,161,438	[M+H]+	methylhexa	50,733	ChEBI=CH
ID00012_1	Unknown	0	139	1,161,438	[M+H]+	tuaminohe	49,143	ChEBI=CH
ID00013_1	Unknown	0	247	1,180,868	[M+H]+	L-Valine	63,815	HMDB=HM
ID00013_1	Unknown	0	247	1,180,868	[M+H]+	Betaine	63,485	HMDB=HM
ID00013_1	Unknown	0	247	1,180,868	[M+H]+	5-Aminope	62,426	HMDB=HM
ID00013_1	Unknown	0	247	1,180,868	[M+H]+	Norvaline	59,787	HMDB=HM
ID00013_1	Unknown	0	247	1,180,868	[M+H]+	N,N-dimeth	5,771	ChEBI=CH
ID00013_1	Unknown	0	247	1,180,868	[M+H]+	N-Methyl-a	56,517	HMDB=HM
ID00013_1	Unknown	0	247	1,180,868	[M+H]+	N,N-dimeth	56,077	ChEBI=CH
ID00013_1	Unknown	0	247	1,180,868	[M+H]+	4R-aminop	55,196	LipidMAPS
ID00013_1	Unknown	0	247	1,180,868	[M+H]+	4-amino-3-	54,881	ChEBI=CH
ID00013_1	Unknown	0	247	1,180,868	[M+H]+	Amyl Nitrite	53,654	HMDB=HM
ID00014_1	Unknown	0	174	118,123	[M+H]+	2-Diethyla	5,816	HMDB=HM
ID00014_1	Unknown	0	174	118,123	[M+H]+	N,N-diethy	55,853	PubChem=
ID00016_0	Unknown	0	207	1,181,231	[M+H]+	2-Diethyla	58,107	HMDB=HM
ID00016_0	Unknown	0	207	1,181,231	[M+H]+	N,N-diethy	55,634	PubChem=
ID00017_1	Unknown	0	220	118,123	[M+H]+	2-Diethyla	59,023	HMDB=HM

ID00017_1	Unknown	0	220	118,123	[M+H]+	N,N-diethy	56,665	PubChem=
ID00022_1	Unknown	0	145	1,220,967	[M+H]+	Phenylethy	65,371	HMDB=HM
ID00022_1	Unknown	0	145	1,220,967	[M+H]+	1-Phenylet	62,157	HMDB=HM
ID00022_1	Unknown	0	145	1,220,967	[M+H]+	2,6-Dimeth	60,421	HMDB=HM
ID00022_1	Unknown	0	145	1,220,967	[M+H]+	N,N-Dimet	57,593	HMDB=HM
ID00022_1	Unknown	0	145	1,220,967	[M+H]+	N-Ethylanil	56,601	ChEBI=CH
ID00022_1	Unknown	0	145	1,220,967	[M+H]+	2-Ethyl-5-m	5,549	HMDB=HM

ID00022_1	Unknown	0	145	1,220,967	[M+H]+	5-Ethyl-2-m	5,549	HMDB=HM
ID00022_1	Unknown	0	145	1,220,967	[M+H]+	2-Propylpyr	5,549	HMDB=HM
ID00022_1	Unknown	0	145	1,220,967	[M+H]+	2,4-dimeth	54,865	ChEBI=CH
ID00022_1	Unknown	0	145	1,220,967	[M+H]+	3,4-dimeth	54,865	ChEBI=CH
ID00024_1	w/o MS2:N	0	259	1,230,555	[M+H]+	Niacinamid	59,231	HMDB=HM
ID00024_1	w/o MS2:N	0	259	1,230,555	[M+H]+	pyridine-3-	55,581	ChEBI=CH
ID00024_1	w/o MS2:N	0	259	1,230,555	[M+H]+	5H-imidazo	53,056	ChEBI=CH
ID00024_1	w/o MS2:N	0	259	1,230,555	[M+H]+	picolinami	52,287	ChEBI=CH
ID00024_1	w/o MS2:N	0	259	1,230,555	[M+H]+	isonicotina	52,287	ChEBI=CH
ID00024_1	w/o MS2:N	0	259	1,230,555	[M+H]+	2-Acetylpyr	51,528	HMDB=HM
ID00026_0	Unknown	0	241	1,260,222	[M+H]+	Taurine	60,833	HMDB=HM
ID00026_0	Unknown	0	241	1,260,222	[M+H]+	methyl met	48,669	ChEBI=CH
ID00029_1	Unknown	0	152	1,301,594	[M+H]+	octodrine	5,268	ChEBI=CH
ID00029_1	Unknown	0	152	1,301,594	[M+H]+	octan-1-am	50,462	ChEBI=CH
ID00031_1	Unknown	0	251	1,320,769	[M+H]+	Creatine	61,313	HMDB=HM
ID00031_1	Unknown	0	251	1,320,769	[M+H]+	Beta-Guani	53,388	HMDB=HM
ID00031_1	Unknown	0	251	1,320,769	[M+H]+	2-[diamino	52,687	PubChem=
ID00031_1	Unknown	0	251	1,320,769	[M+H]+	UNPD1638	52,018	UNPD=UN
ID00033_0	Unknown	0	245	1,370,458	[M+H]+	Hypoxanthi	68,274	HMDB=HM
ID00033_0	Unknown	0	245	1,370,458	[M+H]+	Allopurinol	59,613	HMDB=HM
ID00033_0	Unknown	0	245	1,370,458	[M+H]+	3H-pyrazol	5,88	ChEBI=CH
ID00033_0	Unknown	0	245	1,370,458	[M+H]+	7,9-dihydro	56,765	PubChem=
ID00033_0	Unknown	0	245	1,370,458	[M+H]+	5-amino-1-	56,682	ChEBI=CH
ID00033_0	Unknown	0	245	1,370,458	[M+H]+	1,4-dihydro	55,382	PubChem=
ID00033_0	Unknown	0	245	1,370,458	[M+H]+	1-Pentanes	4,16	HMDB=HM
ID00033_0	Unknown	0	245	1,370,458	[M+H]+	Ethyl isopro	4,16	HMDB=HM
ID00033_0	Unknown	0	245	1,370,458	[M+H]+	Ethyl propy	4,16	HMDB=HM
ID00041_1	Unknown	0	539	1,560,423	[M+H]+	N-Methylet	4,858	HMDB=HM
ID00041_1	Unknown	0	539	1,560,423	[M+H]+	(S)-1-amin	47,112	ChEBI=CH
ID00043_1	Unknown	0	391	162,149	[M+H]+		-1	
ID00045_1	Unknown	0	347	1,650,101	[M+H]+		-1	
ID00047_1	w/o MS2:P	0	370	1,660,864	[M+H]+	L-Phenylal	66,246	HMDB=HM
ID00047_1	w/o MS2:P	0	370	1,660,864	[M+H]+	Norsalsolin	59,211	HMDB=HM
ID00047_1	w/o MS2:P	0	370	1,660,864	[M+H]+	Benzocaine	58,916	HMDB=HM
ID00047_1	w/o MS2:P	0	370	1,660,864	[M+H]+	atrolactam	58,461	ChEBI=CH
ID00047_1	w/o MS2:P	0	370	1,660,864	[M+H]+	(R)-3-amm	58,155	ChEBI=CH
ID00047_1	w/o MS2:P	0	370	1,660,864	[M+H]+	4-Hydroxy-	57,389	HMDB=HM
ID00047_1	w/o MS2:P	0	370	1,660,864	[M+H]+	3-Pyridineb	5,717	HMDB=HM
ID00047_1	w/o MS2:P	0	370	1,660,864	[M+H]+	5-(3-Pyridy	57,046	HMDB=HM
ID00047_1	w/o MS2:P	0	370	1,660,864	[M+H]+	ethenzami	56,772	ChEBI=CH
ID00047_1	w/o MS2:P	0	370	1,660,864	[M+H]+	5-propyl-2-	56,638	ChEBI=CH
ID00048_1	Unknown	0	546	1,730,788	[M+H]+		-1	
ID00051_1	w/o MS2:Ty	0	637	1,820,817	[M+H]+	L-Tyrosine	67,003	HMDB=HM
ID00051_1	w/o MS2:Ty	0	637	1,820,817	[M+H]+	o-Tyrosine	62,837	HMDB=HM

ID00051_1	w/o MS2:Ty	0	637	1,820,817	[M+H]+	Beta-Tyrosi	6,059	HMDB=HM
ID00051_1	w/o MS2:Ty	0	637	1,820,817	[M+H]+	Meta-Tyros	60,059	HMDB=HM
ID00051_1	w/o MS2:Ty	0	637	1,820,817	[M+H]+	L-Threo-3-P	58,966	HMDB=HM
ID00051_1	w/o MS2:Ty	0	637	1,820,817	[M+H]+	4,6,7-Trihy	58,088	HMDB=HM
ID00051_1	w/o MS2:Ty	0	637	1,820,817	[M+H]+	styramate	57,524	ChEBI=CH
ID00051_1	w/o MS2:Ty	0	637	1,820,817	[M+H]+	N-hydroxy-	56,953	ChEBI=CH
ID00051_1	w/o MS2:Ty	0	637	1,820,817	[M+H]+	adrenalone	56,901	ChEBI=CH
ID00051_1	w/o MS2:Ty	0	637	1,820,817	[M+H]+	4-Hydroxy-	5,493	HMDB=HM
ID00052_1	Unknown	0	744	1,830,782	[M+H]+	diisopropyl	47,249	ChEBI=CH
ID00052_1	Unknown	0	744	1,830,782	[M+H]+	triethyl pho	45,933	ChEBI=CH
ID00054_1	Unknown	0	806	1,921,597	[M+H]+		-1	
ID00055_0	Unknown	0	723	1,951,227	[M+H]+	Tetraethyle	54,518	HMDB=HM
ID00055_0	Unknown	0	723	1,951,227	[M+H]+	UNPD2172	51,991	UNPD=UN
ID00055_0	Unknown	0	723	1,951,227	[M+H]+		-1	
ID00056_1	NAE 9:0	0	565	2,021,801	[M+H]+	11-Aminou	53,031	ChEBI=CH
ID00057_2	Unknown	0	214	2,041,233	[M+H]+	L-Acetylcar	64,981	HMDB=HM
ID00057_2	Unknown	0	214	2,041,233	[M+H]+	2-[(2R,5R,6	56,908	ChEBI=CH
ID00057_2	Unknown	0	214	2,041,233	[M+H]+	N-lactoyl-L	55,822	HMDB=HM
ID00058_1	Unknown	0	862	2,051,438	[M+H]+	(2xi,6xi)-7-	53,976	HMDB=HM
ID00058_1	Unknown	0	862	2,051,438	[M+H]+	(1S,2S,4R,	52,656	HMDB=HM
ID00058_1	Unknown	0	862	2,051,438	[M+H]+	(1alpha,2a	52,558	HMDB=HM
ID00058_1	Unknown	0	862	2,051,438	[M+H]+	(2xi,3xi,6E)	52,556	HMDB=HM
ID00058_1	Unknown	0	862	2,051,438	[M+H]+	3,7-Dimeth	52,531	HMDB=HM
ID00058_1	Unknown	0	862	2,051,438	[M+H]+	(3R)-3,10-d	52,265	ChEBI=CH
ID00058_1	Unknown	0	862	2,051,438	[M+H]+	(3R,9R)-3,9	50,904	ChEBI=CH
ID00058_1	Unknown	0	862	2,051,438	[M+H]+	6,6-dimeth	50,716	LipidMAPS
ID00058_1	Unknown	0	862	2,051,438	[M+H]+		-1	
ID00059_1	Unknown	0	283	2,070,573	[M+H]+		-1	
ID00060_1	Unknown	0	693	2,070,572	[M+H]+		-1	
ID00063_1	Unknown	0	419	2,090,546	[M+H]+		-1	
ID00064_1	Unknown	0	535	212,02	[M+H]+		-1	
ID00064_1	Unknown	0	535	212,02	[M+H]+		-1	
ID00064_1	Unknown	0	535	212,02	[M+H]+		-1	
ID00066_0	Unknown	0	322	220,118	[M+H]+	Pantotheni	63,123	HMDB=HM
ID00066_0	Unknown	0	322	220,118	[M+H]+		-1	
ID00067_1	Unknown	0	288	2,211,111	[M+H]+	1-methyl-2	47,001	ChEBI=CH
ID00067_1	Unknown	0	288	2,211,111	[M+H]+	N-(2,6-dim	4,583	ChEBI=CH
ID00067_1	Unknown	0	288	2,211,111	[M+H]+		-1	
ID00072_1	Unknown	0	244	222,086	[M+H]+		-1	

ID00073_1	Unknown	0	243	2,221,703	[M+H]+	-1
ID00073_1	Unknown	0	243	2,221,703	[M+H]+	-1
ID00074_1	Unknown	0	900	2,240,841	[M+H]+	-1
ID00074_1	Unknown	0	900	2,240,841	[M+H]+	-1
ID00075_1	Unknown	0	577	2,260,804	[M+H]+	-1

ID00076_1	Unknown	0	501	2,269,513	[M+H]+		-1	
ID00076_1	Unknown	0	501	2,269,513	[M+H]+		-1	
ID00076_1	Unknown	0	501	2,269,513	[M+H]+		-1	
ID00077_2	Unknown	0	504	2,302,479	[M+H]+	1-deoxytetr	52,765	ChEBI=CH
ID00077_2	Unknown	0	504	2,302,479	[M+H]+	dodecyldim	51,337	ChEBI=CH
ID00081_1	Unknown	0	492	2,380,029	[M+H]+		-1	
ID00081_1	Unknown	0	492	2,380,029	[M+H]+		-1	
ID00081_1	Unknown	0	492	2,380,029	[M+H]+		-1	
ID00082_1	Unknown	0	302	2,380,995	[M+H]+		-1	
ID00085_0	Unknown	0	772	2,391,493	[M+H]+	pentaethyl	50,343	ChEBI=CH
ID00085_0	Unknown	0	772	2,391,493	[M+H]+	pirimicarb	48,473	ChEBI=CH
ID00085_0	Unknown	0	772	2,391,493	[M+H]+		-1	
ID00086_1	Unknown	0	580	2,400,959	[M+H]+		-1	
ID00086_1	Unknown	0	580	2,400,959	[M+H]+		-1	
ID00086_1	Unknown	0	580	2,400,959	[M+H]+		-1	
ID00087_1	w/o MS2:S	0	1156	2,462,426	[M+H]+		-1	
ID00089_1	Unknown	0	1534	2,540,942	[M+H]+		-1	
ID00089_1	Unknown	0	1534	2,540,942	[M+H]+		-1	
ID00090_0	Unknown	0	1645	2,561,759	[M+H]+		-1	
ID00090_0	Unknown	0	1645	2,561,759	[M+H]+		-1	
ID00091_2	Unknown	0	734	2,581,102	[M+H]+	Glyceropho	62,652	HMDB=HM
ID00091_2	Unknown	0	734	2,581,102	[M+H]+		-1	
ID00091_2	Unknown	0	734	2,581,102	[M+H]+		-1	
ID00091_2	Unknown	0	734	2,581,102	[M+H]+		-1	
ID00092_1	NAE 13:0	0	1253	2,582,429	[M+H]+		-1	
ID00093_1	Unknown	0	402	2,600,813	[M+H]+		-1	
ID00093_1	Unknown	0	402	2,600,813	[M+H]+		-1	
ID00093_1	Unknown	0	402	2,600,813	[M+H]+		-1	
ID00093_1	Unknown	0	402	2,600,813	[M+H]+		-1	
ID00094_1	Unknown	0	393	2,620,783	[M+H]+		-1	
ID00094_1	Unknown	0	393	2,620,783	[M+H]+		-1	
ID00094_1	Unknown	0	393	2,620,783	[M+H]+		-1	
ID00094_1	Unknown	0	393	2,620,783	[M+H]+		-1	
ID00096_1	Unknown	0	1360	2,720,602	[M+H]+		-1	
ID00096_1	Unknown	0	1360	2,720,602	[M+H]+		-1	
ID00096_1	Unknown	0	1360	2,720,602	[M+H]+		-1	
ID00096_1	Unknown	0	1360	2,720,602	[M+H]+		-1	
ID00097_1	w/o MS2:S	0	935	2,742,737	[M+H]+		-1	
ID00098_2	w/o MS2:S	0	201	2,742,749	[M+H]+		-1	
ID00100_1	Unknown	0	568	2,811,382	[M+H]+	Nigellic aci	67,394	HMDB=HM
ID00100_1	Unknown	0	568	2,811,382	[M+H]+	13-Hydroxy	66,928	HMDB=HM
ID00100_1	Unknown	0	568	2,811,382	[M+H]+	Crispolide	65,637	HMDB=HM
ID00100_1	Unknown	0	568	2,811,382	[M+H]+	Phaseic ac 6,43		KNApSAcK

ID00100_1	Unknown	0	568	2,811,382	[M+H]+	(+)-Hepteli	6,387	KNApSAcK

ID00100_1	Unknown	0	568	2,811,382	[M+H]+	UNPD2131	63,536	UNPD=UN
ID00100_1	Unknown	0	568	2,811,382	[M+H]+	(-)-Mniopet	6,325	KNApSAcK
ID00100_1	Unknown	0	568	2,811,382	[M+H]+	UNPD4447	63,239	UNPD=UN
ID00100_1	Unknown	0	568	2,811,382	[M+H]+	Biatriospor	63,001	NPA=NPA0
ID00100_1	Unknown	0	568	2,811,382	[M+H]+	UNPD2070	62,981	UNPD=UN
ID00101_3	Unknown	0	1788	2,822,791	[M+H]+	Oleamide	54,795	HMDB=HM
ID00101_3	Unknown	0	1788	2,822,791	[M+H]+	dodemorph	51,316	ChEBI=CH
ID00102_1	Unknown	0	1643	2,830,941	[M+H]+	N-[(2-ethyl	43,944	ChEBI=CH
ID00102_1	Unknown	0	1643	2,830,941	[M+H]+		-1	
ID00102_1	Unknown	0	1643	2,830,941	[M+H]+		-1	
ID00102_1	Unknown	0	1643	2,830,941	[M+H]+		-1	
ID00103_1	Unknown	0	528	2,831,754	[M+H]+	Hexaethyle 5,6		HMDB=HM
ID00103_1	Unknown	0	528	2,831,754	[M+H]+		-1	
ID00103_1	Unknown	0	528	2,831,754	[M+H]+		-1	
ID00103_1	Unknown	0	528	2,831,754	[M+H]+		-1	
ID00104_4	w/o MS2:RI	0	1370	2,842,944	[M+H]+	Octadecan	57,526	HMDB=HM
ID00104_4	w/o MS2:RI	0	1370	2,842,944	[M+H]+	1-deoxy-3-	55,746	ChEBI=CH
ID00104_4	w/o MS2:RI	0	1370	2,842,944	[M+H]+	4-dodecyl-	52,785	ChEBI=CH
ID00104_4	w/o MS2:RI	0	1370	2,842,944	[M+H]+	4-dodecyl-	52,189	ChEBI=CH
ID00105_1	w/o MS2:S	0	1147	2,882,536	[M+H]+	prosopinin	57,785	ChEBI=CH
ID00105_1	w/o MS2:S	0	1147	2,882,536	[M+H]+	Lauroyl die	5,722	HMDB=HM
ID00106_2	w/o MS2:RI	0	314	2,882,895	[M+H]+	C17 Sphing	55,009	LipidMAPS
ID00107_0	Unknown	0	1254	2,901,604	[M+H]+	O-Adipoylc	56,955	HMDB=HM
ID00107_0	Unknown	0	1254	2,901,604	[M+H]+	3-Methylgl	56,878	HMDB=HM
ID00107_0	Unknown	0	1254	2,901,604	[M+H]+	etazolate	48,216	ChEBI=CH
ID00107_0	Unknown	0	1254	2,901,604	[M+H]+		-1	
ID00107_0	Unknown	0	1254	2,901,604	[M+H]+		-1	
ID00108_2	w/o MS2:S	0	306	2,902,685	[M+H]+	C16 phytos	54,797	ChEBI=CH
ID00110_1	Unknown	0	1388	2,950,937	[M+H]+	Sarubicin A	4,884	NPA=NPA0
ID00110_1	Unknown	0	1388	2,950,937	[M+H]+		-1	
ID00110_1	Unknown	0	1388	2,950,937	[M+H]+		-1	
ID00110_1	Unknown	0	1388	2,950,937	[M+H]+		-1	
ID00111_0	Unknown	0	441	3,002,021	[M+H]+		-1	
ID00111_0	Unknown	0	441	3,002,021	[M+H]+		-1	
ID00111_0	Unknown	0	441	3,002,021	[M+H]+		-1	
ID00112_4	Unknown	0	1745	3,103,108	[M+H]+	N-Hexadec	50,752	HMDB=HM
ID00113_6	Unknown	0	1823	3,123,259	[M+H]+	2,6-dimeth	49,576	ChEBI=CH
ID00114_1	Unknown	0	1033	3,171,152	[M+H]+	UNPD4804	49,567	UNPD=UN
ID00114_1	Unknown	0	1033	3,171,152	[M+H]+	1-[2-[(2-me	44,626	ChEBI=CH
ID00114_1	Unknown	0	1033	3,171,152	[M+H]+		-1	
ID00114_1	Unknown	0	1033	3,171,152	[M+H]+		-1	
ID00115_2	w/o MS2:RI	0	275	3,182,999	[M+H]+	Phytosphin	62,099	HMDB=HM
ID00116_2	w/o MS2:RI	0	1164	3,182,999	[M+H]+	Phytosphin	61,848	HMDB=HM

ID00117_1	Unknown	0	1113	319,147	[M+H]+	-1

ID00117_1	Unknown	0	1113	319,147	[M+H]+		-1	
ID00117_1	Unknown	0	1113	319,147	[M+H]+		-1	
ID00117_1	Unknown	0	1113	319,147	[M+H]+		-1	
ID00118_1	Unknown	0	1906	3,231,472	[M+H]+	adenosine-	5,112	ChEBI=CH
ID00118_1	Unknown	0	1906	3,231,472	[M+H]+		-1	
ID00118_1	Unknown	0	1906	3,231,472	[M+H]+		-1	
ID00118_1	Unknown	0	1906	3,231,472	[M+H]+		-1	
ID00119_1	Unknown	0	1616	3,242,102	[M+H]+		-1	
ID00119_1	Unknown	0	1616	3,242,102	[M+H]+		-1	
ID00119_1	Unknown	0	1616	3,242,102	[M+H]+		-1	
ID00119_1	Unknown	0	1616	3,242,102	[M+H]+		-1	
ID00120_1	Unknown	0	392	3,251,051	[M+H]+		-1	
ID00120_1	Unknown	0	392	3,251,051	[M+H]+		-1	
ID00120_1	Unknown	0	392	3,251,051	[M+H]+		-1	
ID00120_1	Unknown	0	392	3,251,051	[M+H]+		-1	
ID00121_1	Unknown	0	1006	3,271,207	[M+H]+	8-azido-5-	5,005	ChEBI=CH
ID00121_1	Unknown	0	1006	3,271,207	[M+H]+		-1	
ID00121_1	Unknown	0	1006	3,271,207	[M+H]+		-1	
ID00121_1	Unknown	0	1006	3,271,207	[M+H]+		-1	
ID00122_1	Unknown	0	573	3,272,018	[M+H]+	Heptaethyl	55,986	HMDB=HM
ID00122_1	Unknown	0	573	3,272,018	[M+H]+		-1	
ID00122_1	Unknown	0	573	3,272,018	[M+H]+		-1	
ID00122_1	Unknown	0	573	3,272,018	[M+H]+		-1	
ID00123_0	Unknown	0	883	3,341,866	[M+H]+		-1	
ID00123_0	Unknown	0	883	3,341,866	[M+H]+		-1	
ID00123_0	Unknown	0	883	3,341,866	[M+H]+		-1	
ID00123_0	Unknown	0	883	3,341,866	[M+H]+		-1	
ID00124_2	w/o MS2:D	0	1142	3,342,953	[M+H]+		-1	
ID00124_2	w/o MS2:D	0	1142	3,342,953	[M+H]+		-1	
ID00125_2	Unknown	0	1320	3,363,106	[M+H]+		-1	
ID00126_5	Unknown	0	1425	3,363,264	[M+H]+	Pipericine	52,286	HMDB=HM
ID00127_1	Unknown	0	826	3,382,257	[M+H]+		-1	
ID00127_1	Unknown	0	826	3,382,257	[M+H]+		-1	
ID00127_1	Unknown	0	826	3,382,257	[M+H]+		-1	
ID00127_1	Unknown	0	826	3,382,257	[M+H]+		-1	
ID00128_6	w/o MS2:1	0	459	338,341	[M+H]+	6-cis-Doco	52,322	ChEBI=CH
ID00129_1	Unknown	0	1424	3,402,603	[M+H]+		-1	
ID00129_1	Unknown	0	1424	3,402,603	[M+H]+		-1	
ID00129_1	Unknown	0	1424	3,402,603	[M+H]+		-1	
ID00129_1	Unknown	0	1424	3,402,603	[M+H]+		-1	
ID00130_1	Unknown	0	1505	3,431,152	[M+H]+	4-nitrophen	47,011	ChEBI=CH
ID00130_1	Unknown	0	1505	3,431,152	[M+H]+	2-nitrophen	46,774	ChEBI=CH
ID00130_1	Unknown	0	1505	3,431,152	[M+H]+		-1	
ID00130_1	Unknown	0	1505	3,431,152	[M+H]+		-1	

ID00130_1	Unknown	0	1505	3,431,152	[M+H]+		-1	
ID00131_1	Unknown	0	267	3,442,285	[M+H]+		-1	
ID00131_1	Unknown	0	267	3,442,285	[M+H]+		-1	
ID00131_1	Unknown	0	267	3,442,285	[M+H]+		-1	
ID00131_1	Unknown	0	267	3,442,285	[M+H]+		-1	
ID00132_1	Unknown	0	1374	3,492,648	[M+H]+		-1	
ID00132_1	Unknown	0	1374	3,492,648	[M+H]+		-1	
ID00132_1	Unknown	0	1374	3,492,648	[M+H]+		-1	
ID00132_1	Unknown	0	1374	3,492,648	[M+H]+		-1	
ID00133_1	Unknown	0	1085	3,522,414	[M+H]+		-1	
ID00133_1	Unknown	0	1085	3,522,414	[M+H]+		-1	
ID00133_1	Unknown	0	1085	3,522,414	[M+H]+		-1	
ID00133_1	Unknown	0	1085	3,522,414	[M+H]+		-1	
ID00134_1	Unknown	0	1739	3,551,152	[M+H]+	N-[3-[[dieth	49,678	ChEBI=CH
ID00134_1	Unknown	0	1739	3,551,152	[M+H]+	Gliovictin;	4,89	KNApSAcK
ID00134_1	Unknown	0	1739	3,551,152	[M+H]+	Podocarpia	4,668	NPA=NPA0
ID00134_1	Unknown	0	1739	3,551,152	[M+H]+		-1	
ID00134_1	Unknown	0	1739	3,551,152	[M+H]+		-1	
ID00135_1	Unknown	0	1985	3,571,315	[M+H]+	2-(2,4-dihy	57,061	HMDB=HM
ID00135_1	Unknown	0	1985	3,571,315	[M+H]+	Kievitone	57,034	HMDB=HM
ID00135_1	Unknown	0	1985	3,571,315	[M+H]+	Xanthoxylo	55,925	HMDB=HM
ID00135_1	Unknown	0	1985	3,571,315	[M+H]+	Sigmoidin	54,554	KNApSAcK
ID00135_1	Unknown	0	1985	3,571,315	[M+H]+	5-Deoxykie	54,492	HMDB=HM
ID00135_1	Unknown	0	1985	3,571,315	[M+H]+	Monoteson	54,474	KNApSAcK
ID00135_1	Unknown	0	1985	3,571,315	[M+H]+	Cubebin	53,712	HMDB=HM
ID00135_1	Unknown	0	1985	3,571,315	[M+H]+	UNPD2231	53,607	UNPD=UN
ID00135_1	Unknown	0	1985	3,571,315	[M+H]+	(-)-Pluviato	53,588	KNApSAcK
ID00135_1	Unknown	0	1985	3,571,315	[M+H]+	Flowerone;	53,539	KNApSAcK
ID00136_1	Unknown	0	1382	3,592,326	[M+2H]2+	Ribocyclop	53,754	NPA=NPA0
ID00136_1	Unknown	0	1382	3,592,326	[M+2H]2+		-1	
ID00136_1	Unknown	0	1382	3,592,326	[M+2H]2+		-1	
ID00136_1	Unknown	0	1382	3,592,326	[M+2H]2+		-1	
ID00137_2	w/o MS2:D	0	964	3,623,254	[M+H]+	2-amino-1,	54,075	ChEBI=CH
ID00137_2	w/o MS2:D	0	964	3,623,254	[M+H]+		-1	
ID00138_1	Unknown	0	845	362,926	[M+H]+		-1	
ID00138_1	Unknown	0	845	362,926	[M+H]+		-1	
ID00138_1	Unknown	0	845	362,926	[M+H]+		-1	
ID00138_1	Unknown	0	845	362,926	[M+H]+		-1	
ID00139_8	Unknown	0	2049	3,663,736	[M+H]+		-1	
ID00140_0	Unknown	0	741	3,702,697	[M+2H]2+		-1	
ID00140_0	Unknown	0	741	3,702,697	[M+2H]2+		-1	
ID00140_0	Unknown	0	741	3,702,697	[M+2H]2+		-1	
ID00140_0	Unknown	0	741	3,702,697	[M+2H]2+		-1	
ID00141_2	CAR 14:1	0	1728	3,702,949	[M+H]+	cis-5-Tetra	58,444	HMDB=HM

ID00141_2 CAR 14:1	0	1728	3,702,949	[M+H]+	trans-2-Tet	5,663	HMDB=HM
ID00141_2 CAR 14:1	0	1728	3,702,949	[M+H]+	O-(2-tetrad	55,633	HMDB=HM
ID00141_2 CAR 14:1	0	1728	3,702,949	[M+H]+	O-[(9Z)-tetr	53,951	ChEBI=CH
ID00141_2 CAR 14:1	0	1728	3,702,949	[M+H]+	Besarhana	5,191	KNApSAcK
ID00141_2 CAR 14:1	0	1728	3,702,949	[M+H]+		-1	
ID00141_2 CAR 14:1	0	1728	3,702,949	[M+H]+		-1	
ID00141_2 CAR 14:1	0	1728	3,702,949	[M+H]+		-1	
ID00142_1 w/o MS2:RI	0	881	3,712,279	[M+H]+	Octaethyle	53,839	HMDB=HM
ID00142_1 w/o MS2:RI	0	881	3,712,279	[M+H]+	Ala-Leu-Ala	50,368	ChEBI=CH
ID00142_1 w/o MS2:RI	0	881	3,712,279	[M+H]+		-1	
ID00142_1 w/o MS2:RI	0	881	3,712,279	[M+H]+		-1	
ID00143_2 Unknown	0	1492	380,337	[M+H]+		-1	
ID00143_2 Unknown	0	1492	380,337	[M+H]+		-1	
ID00144_1 Unknown	0	913	3,811,891	[M+H]+	Chaparrin	54,022	KNApSAcK
ID00144_1 Unknown	0	913	3,811,891	[M+H]+	Neurolenin	52,436	KNApSAcK
ID00144_1 Unknown	0	913	3,811,891	[M+H]+	Neurolenin	52,065	KNApSAcK
ID00144_1 Unknown	0	913	3,811,891	[M+H]+	6b-Angeloy	51,703	HMDB=HM
ID00144_1 Unknown	0	913	3,811,891	[M+H]+	UNPD1340	51,694	UNPD=UN
ID00144_1 Unknown	0	913	3,811,891	[M+H]+	Cinncassio	51,146	HMDB=HM
ID00144_1 Unknown	0	913	3,811,891	[M+H]+	Deacetylis	5,109	HMDB=HM
ID00144_1 Unknown	0	913	3,811,891	[M+H]+	3,4,5-trihyd	49,655	HMDB=HM
ID00144_1 Unknown	0	913	3,811,891	[M+H]+	Pteroside B	48,749	HMDB=HM
ID00144_1 Unknown	0	913	3,811,891	[M+H]+		-1	
ID00145_1 Unknown	0	901	3,831,465	[M+H]+	N-(3-isoxaz	5,004	ChEBI=CH
ID00145_1 Unknown	0	901	3,831,465	[M+H]+	N-[3-[[dieth	49,344	ChEBI=CH
ID00145_1 Unknown	0	901	3,831,465	[M+H]+		-1	
ID00145_1 Unknown	0	901	3,831,465	[M+H]+		-1	
ID00146_1 Unknown	0	728	3,871,799	[M+H]+	(+)-ganoth	62,629	NPA=NPA0
ID00146_1 Unknown	0	728	3,871,799	[M+H]+	UNPD4289	62,412	UNPD=UN
ID00146_1 Unknown	0	728	3,871,799	[M+H]+	Astellolide	61,502	NPA=NPA0
ID00146_1 Unknown	0	728	3,871,799	[M+H]+	Astellolide	61,488	NPA=NPA0
ID00146_1 Unknown	0	728	3,871,799	[M+H]+	Glabramyc	61,389	NPA=NPA0
ID00146_1 Unknown	0	728	3,871,799	[M+H]+	Pitholide B	60,609	NPA=NPA0
ID00146_1 Unknown	0	728	3,871,799	[M+H]+	(-)-Furaqui	6,015	KNApSAcK
ID00146_1 Unknown	0	728	3,871,799	[M+H]+	UNPD1052	59,598	UNPD=UN
ID00146_1 Unknown	0	728	3,871,799	[M+H]+	UNPD3577	59,482	UNPD=UN
ID00146_1 Unknown	0	728	3,871,799	[M+H]+	(+)-(7S,8R, 5,94		ChEBI=CH
ID00147_1 Unknown	0	1199	3,871,935	[M+H]+		-1	
ID00147_1 Unknown	0	1199	3,871,935	[M+H]+		-1	
ID00147_1 Unknown	0	1199	3,871,935	[M+H]+		-1	
ID00147_1 Unknown	0	1199	3,871,935	[M+H]+		-1	
ID00148_1 w/o MS2:N	0	457	3,882,552	[M+H]+		-1	
ID00148_1 w/o MS2:N	0	457	3,882,552	[M+H]+		-1	
ID00148_1 w/o MS2:N	0	457	3,882,552	[M+H]+		-1	

ID00148_1	w/o MS2:N	0	457	3,882,552	[M+H]+		-1	
ID00149_0	Unknown	0	797	3,942,231	[M+H]+	Spliceostat	59,836	NPA=NPA0
ID00149_0	Unknown	0	797	3,942,231	[M+H]+		-1	
ID00149_0	Unknown	0	797	3,942,231	[M+H]+		-1	
ID00149_0	Unknown	0	797	3,942,231	[M+H]+		-1	
ID00150_0	Unknown	0	520	396,801	[M+Na]2+		-1	
ID00150_0	Unknown	0	520	396,801	[M+Na]2+		-1	
ID00150_0	Unknown	0	520	396,801	[M+Na]2+		-1	
ID00150_0	Unknown	0	520	396,801	[M+Na]2+		-1	
ID00150_0	Unknown	0	520	396,801	[M+Na]2+		-1	
ID00151_1	Unknown	0	318	3,968,028	[M+2H]2+		-1	
ID00151_1	Unknown	0	318	3,968,028	[M+2H]2+		-1	
ID00151_1	Unknown	0	318	3,968,028	[M+2H]2+		-1	
ID00151_1	Unknown	0	318	3,968,028	[M+2H]2+		-1	
ID00151_1	Unknown	0	318	3,968,028	[M+2H]2+		-1	
ID00152_1	Unknown	0	852	3,971,628	[M+H]+	Rotenonic	58,068	KNApSAcK
ID00152_1	Unknown	0	852	3,971,628	[M+H]+	7a-O-Meth	55,184	KNApSAcK
ID00152_1	Unknown	0	852	3,971,628	[M+H]+	Ixerochinol	54,803	KNApSAcK
ID00152_1	Unknown	0	852	3,971,628	[M+H]+	cochinchin	54,653	ChEBI=CH
ID00152_1	Unknown	0	852	3,971,628	[M+H]+	Rotenol	54,447	KNApSAcK
ID00152_1	Unknown	0	852	3,971,628	[M+H]+	Glabrachal	5,434	KNApSAcK
ID00152_1	Unknown	0	852	3,971,628	[M+H]+	BR-Xantho	53,868	HMDB=HM
ID00152_1	Unknown	0	852	3,971,628	[M+H]+	5-O-demet	53,838	ChEBI=CH
ID00152_1	Unknown	0	852	3,971,628	[M+H]+	egonol-2-m	53,664	ChEBI=CH
ID00152_1	Unknown	0	852	3,971,628	[M+H]+	Ponganone	53,636	KNApSAcK
ID00153_1	Unknown	0	1428	3,972,003	[M+H]+	(3'x,5'a,9'x,	63,653	HMDB=HM
ID00153_1	Unknown	0	1428	3,972,003	[M+H]+	Fukanemar	6,281	KNApSAcK
ID00153_1	Unknown	0	1428	3,972,003	[M+H]+	1-hydroxyx	62,518	NPA=NPA0
ID00153_1	Unknown	0	1428	3,972,003	[M+H]+	(2R*,3R*)-	62,149	ChEBI=CH
ID00153_1	Unknown	0	1428	3,972,003	[M+H]+	Fukanefuro	61,931	KNApSAcK
ID00153_1	Unknown	0	1428	3,972,003	[M+H]+	22-hydroxy	61,569	NPA=NPA0
ID00153_1	Unknown	0	1428	3,972,003	[M+H]+	alpha,4,2'-	61,291	KNApSAcK
ID00153_1	Unknown	0	1428	3,972,003	[M+H]+	(S)-(E)-2'-(	60,634	HMDB=HM
ID00153_1	Unknown	0	1428	3,972,003	[M+H]+	(S)-(E)-8-(3	60,542	HMDB=HM
ID00153_1	Unknown	0	1428	3,972,003	[M+H]+	UNPD1352	59,622	UNPD=UN
ID00154_1	Unknown	0	1172	3,992,507	[M+H]+	Antibiotic B	50,027	KNApSAcK
ID00154_1	Unknown	0	1172	3,992,507	[M+H]+	tris(2-butox	46,916	ChEBI=CH
ID00154_1	Unknown	0	1172	3,992,507	[M+H]+		-1	
ID00154_1	Unknown	0	1172	3,992,507	[M+H]+		-1	
ID00154_1	Unknown	0	1172	3,992,507	[M+H]+		-1	
ID00155_1	Unknown	0	1230	4,042,077	[M+H]+	Torrubiello	59,726	ChEBI=CH
ID00155_1	Unknown	0	1230	4,042,077	[M+H]+	Jomthonic	59,559	NPA=NPA0
ID00155_1	Unknown	0	1230	4,042,077	[M+H]+	(1R,4S,5R)	59,163	PubChem=
ID00155_1	Unknown	0	1230	4,042,077	[M+H]+	6beta-[N-(c	59,123	ChEBI=CH

ID00155_1	Unknown	0	1230	4,042,077	[M+H]+	O-benzoyle	5,849	ChEBI=CH
ID00155_1	Unknown	0	1230	4,042,077	[M+H]+	2'-Hydroxy	57,485	KNApSAcK
ID00155_1	Unknown	0	1230	4,042,077	[M+H]+	N-benzoyl-	49,526	ChEBI=CH
ID00155_1	Unknown	0	1230	4,042,077	[M+H]+	N2,N6-bis[	48,405	ChEBI=CH
ID00155_1	Unknown	0	1230	4,042,077	[M+H]+		-1	
ID00155_1	Unknown	0	1230	4,042,077	[M+H]+		-1	
ID00156_1	Unknown	0	1180	4,058,062	[M+2H]2+		-1	
ID00156_1	Unknown	0	1180	4,058,062	[M+2H]2+		-1	
ID00156_1	Unknown	0	1180	4,058,062	[M+2H]2+		-1	
ID00156_1	Unknown	0	1180	4,058,062	[M+2H]2+		-1	
ID00156_1	Unknown	0	1180	4,058,062	[M+2H]2+		-1	
ID00157_1	RIKEN P-VS	0	731	4,152,123	[M+H]+	Eplerenone	62,282	HMDB=HM
ID00157_1	RIKEN P-VS	0	731	4,152,123	[M+H]+	Chaetopen	59,233	NPA=NPA0
ID00157_1	RIKEN P-VS	0	731	4,152,123	[M+H]+	Armillarin	58,886	HMDB=HM
ID00157_1	RIKEN P-VS	0	731	4,152,123	[M+H]+	4-O-Methyl	58,787	HMDB=HM
ID00157_1	RIKEN P-VS	0	731	4,152,123	[M+H]+	6'-dechloro	58,764	NPA=NPA0
ID00157_1	RIKEN P-VS	0	731	4,152,123	[M+H]+	Armillaripin	58,572	HMDB=HM
ID00157_1	RIKEN P-VS	0	731	4,152,123	[M+H]+	Austinoneo	57,955	NPA=NPA0
ID00157_1	RIKEN P-VS	0	731	4,152,123	[M+H]+	UNPD4905	56,608	UNPD=UN
ID00157_1	RIKEN P-VS	0	731	4,152,123	[M+H]+	Estra-1,3,5	56,456	ChEBI=CH
ID00157_1	RIKEN P-VS	0	731	4,152,123	[M+H]+	Talaromyci	54,884	NPA=NPA0
ID00159_1	Unknown	0	1479	415,774	[M+2H]2+		-1	
ID00159_1	Unknown	0	1479	415,774	[M+2H]2+		-1	
ID00159_1	Unknown	0	1479	415,774	[M+2H]2+		-1	
ID00159_1	Unknown	0	1479	415,774	[M+2H]2+		-1	
ID00159_1	Unknown	0	1479	415,774	[M+2H]2+		-1	
ID00160_1	Unknown	0	981	4,159,834	[M+H]+		-1	
ID00160_1	Unknown	0	981	4,159,834	[M+H]+		-1	
ID00160_1	Unknown	0	981	4,159,834	[M+H]+		-1	
ID00160_1	Unknown	0	981	4,159,834	[M+H]+		-1	
ID00160_1	Unknown	0	981	4,159,834	[M+H]+		-1	
ID00161_1	Unknown	0	1370	4,167,976	[M+H]+		-1	
ID00161_1	Unknown	0	1370	4,167,976	[M+H]+		-1	
ID00162_1	w/o MS2:N	0	1042	4,222,895	[M+H]+	Talatisamin	55,765	KNApSAcK
ID00162_1	w/o MS2:N	0	1042	4,222,895	[M+H]+		-1	
ID00162_1	w/o MS2:N	0	1042	4,222,895	[M+H]+		-1	
ID00162_1	w/o MS2:N	0	1042	4,222,895	[M+H]+		-1	
ID00162_1	w/o MS2:N	0	1042	4,222,895	[M+H]+		-1	
ID00163_2	Unknown	0	1195	4,242,639	[M+H]+		-1	
ID00163_2	Unknown	0	1195	4,242,639	[M+H]+		-1	
ID00163_2	Unknown	0	1195	4,242,639	[M+H]+		-1	
ID00163_2	Unknown	0	1195	4,242,639	[M+H]+		-1	
ID00163_2	Unknown	0	1195	4,242,639	[M+H]+		-1	
ID00164_2	Unknown	0	1530	4,243,642	[M+H]+		-1	

ID00164_2	Unknown	0	1530	4,243,642	[M+H]+		-1	
ID00164_2	Unknown	0	1530	4,243,642	[M+H]+		-1	
ID00164_2	Unknown	0	1530	4,243,642	[M+H]+		-1	
ID00165_0	Unknown	0	771	4,268,119	[M+2H]2+		-1	
ID00165_0	Unknown	0	771	4,268,119	[M+2H]2+		-1	
ID00165_0	Unknown	0	771	4,268,119	[M+2H]2+		-1	
ID00165_0	Unknown	0	771	4,268,119	[M+2H]2+		-1	
ID00165_0	Unknown	0	771	4,268,119	[M+2H]2+		-1	
ID00166_1	RIKEN P-VS	0	930	432,239	[M+H]+	Myxopyroni	57,694	KNApSAcK
ID00166_1	RIKEN P-VS	0	930	432,239	[M+H]+	UNPD3201	56,458	UNPD=UN
ID00166_1	RIKEN P-VS	0	930	432,239	[M+H]+		-1	
ID00166_1	RIKEN P-VS	0	930	432,239	[M+H]+		-1	
ID00166_1	RIKEN P-VS	0	930	432,239	[M+H]+		-1	
ID00166_1	RIKEN P-VS	0	930	432,239	[M+H]+		-1	
ID00167_1	w/o MS2:N	0	474	4,322,801	[M+H]+	Istamycin C	54,117	ChEBI=CH
ID00167_1	w/o MS2:N	0	474	4,322,801	[M+H]+		-1	
ID00167_1	w/o MS2:N	0	474	4,322,801	[M+H]+		-1	
ID00167_1	w/o MS2:N	0	474	4,322,801	[M+H]+		-1	
ID00167_1	w/o MS2:N	0	474	4,322,801	[M+H]+		-1	
ID00168_1	Unknown	0	884	4,371,935	[M+H]+	Deisovaler	57,989	KNApSAcK
ID00168_1	Unknown	0	884	4,371,935	[M+H]+		-1	
ID00168_1	Unknown	0	884	4,371,935	[M+H]+		-1	
ID00168_1	Unknown	0	884	4,371,935	[M+H]+		-1	
ID00168_1	Unknown	0	884	4,371,935	[M+H]+		-1	
ID00169_1	Unknown	0	1478	4,461,442	[M+H]+	Azicemicin	57,938	KNApSAcK
ID00169_1	Unknown	0	1478	4,461,442	[M+H]+	adenosine	56,188	ChEBI=CH
ID00169_1	Unknown	0	1478	4,461,442	[M+H]+	N-[4,6-bis(	4,998	ChEBI=CH
ID00169_1	Unknown	0	1478	4,461,442	[M+H]+		-1	
ID00169_1	Unknown	0	1478	4,461,442	[M+H]+		-1	
ID00170_1	Unknown	0	554	4,531,674	[M+K]+	Eplerenone	60,784	HMDB=HM
ID00170_1	Unknown	0	554	4,531,674	[M+K]+	Armillarin	60,187	HMDB=HM
ID00170_1	Unknown	0	554	4,531,674	[M+K]+	Armillaripin	59,112	HMDB=HM
ID00170_1	Unknown	0	554	4,531,674	[M+K]+	4-O-Methyl	59,098	HMDB=HM
ID00170_1	Unknown	0	554	4,531,674	[M+K]+	6'-dechloro	58,601	NPA=NPA0
ID00170_1	Unknown	0	554	4,531,674	[M+K]+	Austinoneo	56,818	NPA=NPA0
ID00170_1	Unknown	0	554	4,531,674	[M+K]+	Chaetopen	55,901	NPA=NPA0
ID00170_1	Unknown	0	554	4,531,674	[M+K]+	Estra-1,3,5	55,857	ChEBI=CH
ID00170_1	Unknown	0	554	4,531,674	[M+K]+	Clausarino	55,362	HMDB=HM
ID00170_1	Unknown	0	554	4,531,674	[M+K]+	UNPD4905	54,857	UNPD=UN
ID00171_0	Unknown	0	505	4,533,443	[M+2H]2+		-1	
ID00171_0	Unknown	0	505	4,533,443	[M+2H]2+		-1	
ID00171_0	Unknown	0	505	4,533,443	[M+2H]2+		-1	
ID00171_0	Unknown	0	505	4,533,443	[M+2H]2+		-1	
ID00171_0	Unknown	0	505	4,533,443	[M+2H]2+		-1	

ID00172_1	Unknown	0	388	4,533,442	[M+2H]2+		-1	
ID00172_1	Unknown	0	388	4,533,442	[M+2H]2+		-1	
ID00172_1	Unknown	0	388	4,533,442	[M+2H]2+		-1	
ID00172_1	Unknown	0	388	4,533,442	[M+2H]2+		-1	
ID00172_1	Unknown	0	388	4,533,442	[M+2H]2+		-1	
ID00173_1	Unknown	0	1308	4,533,442	[M+2H]2+		-1	
ID00173_1	Unknown	0	1308	4,533,442	[M+2H]2+		-1	
ID00173_1	Unknown	0	1308	4,533,442	[M+2H]2+		-1	
ID00173_1	Unknown	0	1308	4,533,442	[M+2H]2+		-1	
ID00173_1	Unknown	0	1308	4,533,442	[M+2H]2+		-1	
ID00175_1	w/o MS2:N	0	991	459,346	[M+H]+	1alpha,25-	53,548	LipidMAPS
ID00175_1	w/o MS2:N	0	991	459,346	[M+H]+	UNPD2261	52,998	UNPD=UN
ID00175_1	w/o MS2:N	0	991	459,346	[M+H]+	1alpha,25-	52,978	LipidMAPS
ID00175_1	w/o MS2:N	0	991	459,346	[M+H]+	18-acetoxy	52,956	ChEBI=CH
ID00175_1	w/o MS2:N	0	991	459,346	[M+H]+	UNPD2040	52,677	UNPD=UN
ID00175_1	w/o MS2:N	0	991	459,346	[M+H]+	fruticoside	52,101	ChEBI=CH
ID00175_1	w/o MS2:N	0	991	459,346	[M+H]+	(3beta,17a	51,909	HMDB=HM
ID00175_1	w/o MS2:N	0	991	459,346	[M+H]+	Stolonifero	51,896	LipidMAPS
ID00175_1	w/o MS2:N	0	991	459,346	[M+H]+	(3beta,5alp	5,183	HMDB=HM
ID00175_1	w/o MS2:N	0	991	459,346	[M+H]+	18-acetoxy	5,171	LipidMAPS
ID00177_1	RIKEN P-VS	0	676	4,602,696	[M+H]+	sch 21376	6,083	ChEBI=CH
ID00177_1	RIKEN P-VS	0	676	4,602,696	[M+H]+	T-2636 E	60,154	NPA=NPA0
ID00177_1	RIKEN P-VS	0	676	4,602,696	[M+H]+	UNPD1697	59,639	UNPD=UN
ID00177_1	RIKEN P-VS	0	676	4,602,696	[M+H]+	UNPD2219	59,542	UNPD=UN
ID00177_1	RIKEN P-VS	0	676	4,602,696	[M+H]+	Arthpyrone	59,401	NPA=NPA0
ID00177_1	RIKEN P-VS	0	676	4,602,696	[M+H]+	UNPD5780	59,357	UNPD=UN
ID00177_1	RIKEN P-VS	0	676	4,602,696	[M+H]+	UNPD2085	59,171	UNPD=UN
ID00177_1	RIKEN P-VS	0	676	4,602,696	[M+H]+	Militarinon	59,059	KNApSAcK
ID00177_1	RIKEN P-VS	0	676	4,602,696	[M+H]+	Mohangic a	58,896	NPA=NPA0
ID00177_1	RIKEN P-VS	0	676	4,602,696	[M+H]+	4-(2,3-dihy	48,306	ChEBI=CH
ID00178_1	Unknown	0	1563	4,623,477	[M+H]+		-1	
ID00178_1	Unknown	0	1563	4,623,477	[M+H]+		-1	
ID00178_1	Unknown	0	1563	4,623,477	[M+H]+		-1	
ID00178_1	Unknown	0	1563	4,623,477	[M+H]+		-1	
ID00178_1	Unknown	0	1563	4,623,477	[M+H]+		-1	
ID00179_1	Unknown	0	1125	473,341	[M+2H]2+		-1	
ID00179_1	Unknown	0	1125	473,341	[M+2H]2+		-1	
ID00179_1	Unknown	0	1125	473,341	[M+2H]2+		-1	
ID00179_1	Unknown	0	1125	473,341	[M+2H]2+		-1	
ID00179_1	Unknown	0	1125	473,341	[M+2H]2+		-1	
ID00180_1	w/o MS2:ST	0	717	4,742,851	[M+H]+	prednisola	60,881	ChEBI=CH
ID00180_1	w/o MS2:ST	0	717	4,742,851	[M+H]+	Mohangic a	58,193	NPA=NPA0
ID00180_1	w/o MS2:ST	0	717	4,742,851	[M+H]+		-1	
ID00180_1	w/o MS2:ST	0	717	4,742,851	[M+H]+		-1	

ID00180_1	w/o MS2:ST	0	717	4,742,851	[M+H]+		-1	
ID00180_1	w/o MS2:ST	0	717	4,742,851	[M+H]+		-1	
ID00181_1	Unknown	0	333	4,753,267	[M+H]+	3-cyclohex	50,532	ChEBI=CH
ID00181_1	Unknown	0	333	4,753,267	[M+H]+	N-[(2R,3S)-	49,351	ChEBI=CH
ID00181_1	Unknown	0	333	4,753,267	[M+H]+		-1	
ID00181_1	Unknown	0	333	4,753,267	[M+H]+		-1	
ID00181_1	Unknown	0	333	4,753,267	[M+H]+		-1	
ID00181_1	Unknown	0	333	4,753,267	[M+H]+		-1	
ID00182_1	w/o MS2:SL	0	366	4,763,055	[M+H]+	Netilmicin	56,544	HMDB=HM
ID00182_1	w/o MS2:SL	0	366	4,763,055	[M+H]+		-1	
ID00182_1	w/o MS2:SL	0	366	4,763,055	[M+H]+		-1	
ID00182_1	w/o MS2:SL	0	366	4,763,055	[M+H]+		-1	
ID00182_1	w/o MS2:SL	0	366	4,763,055	[M+H]+		-1	
ID00183_1	Unknown	0	671	4,773,792	[M+H]+	1-O-alpha-	47,301	LipidMAPS
ID00183_1	Unknown	0	671	4,773,792	[M+H]+		-1	
ID00183_1	Unknown	0	671	4,773,792	[M+H]+		-1	
ID00183_1	Unknown	0	671	4,773,792	[M+H]+		-1	
ID00183_1	Unknown	0	671	4,773,792	[M+H]+		-1	
ID00184_2	LPE 18:2	0	1003	4,782,923	[M+H]+	LysoPE(18:	62,077	HMDB=HM
ID00184_2	LPE 18:2	0	1003	4,782,923	[M+H]+	LysoPE(0:0	60,568	HMDB=HM
ID00184_2	LPE 18:2	0	1003	4,782,923	[M+H]+	Simplicilliu	52,395	NPA=NPA0
ID00184_2	LPE 18:2	0	1003	4,782,923	[M+H]+	aminopote	5,135	ChEBI=CH
ID00184_2	LPE 18:2	0	1003	4,782,923	[M+H]+		-1	
ID00184_2	LPE 18:2	0	1003	4,782,923	[M+H]+		-1	
ID00185_4	LPE 18:0	0	1547	4,823,243	[M+H]+	LysoPE(18:	62,778	HMDB=HM
ID00185_4	LPE 18:0	0	1547	4,823,243	[M+H]+	LysoPC(15	61,889	HMDB=HM
ID00185_4	LPE 18:0	0	1547	4,823,243	[M+H]+	LysoPE(0:0	59,882	HMDB=HM
ID00185_4	LPE 18:0	0	1547	4,823,243	[M+H]+	1-hexadecy	55,913	ChEBI=CH
ID00185_4	LPE 18:0	0	1547	4,823,243	[M+H]+		-1	
ID00185_4	LPE 18:0	0	1547	4,823,243	[M+H]+		-1	
ID00185_4	LPE 18:0	0	1547	4,823,243	[M+H]+		-1	
ID00185_4	LPE 18:0	0	1547	4,823,243	[M+H]+		-1	
ID00186_0	Unknown	0	778	4,833,535	[M+2H]2+		-1	
ID00186_0	Unknown	0	778	4,833,535	[M+2H]2+		-1	
ID00186_0	Unknown	0	778	4,833,535	[M+2H]2+		-1	
ID00186_0	Unknown	0	778	4,833,535	[M+2H]2+		-1	
ID00186_0	Unknown	0	778	4,833,535	[M+2H]2+		-1	
ID00187_2	Unknown	0	1417	4,852,913	[M+H]+	Inflatin B	6,092	ChEBI=CH
ID00187_2	Unknown	0	1417	4,852,913	[M+H]+	UNPD1975	60,649	UNPD=UN
ID00187_2	Unknown	0	1417	4,852,913	[M+H]+	UNPD2275	60,438	UNPD=UN
ID00187_2	Unknown	0	1417	4,852,913	[M+H]+	Stigmatelli	59,132	ChEBI=CH
ID00187_2	Unknown	0	1417	4,852,913	[M+H]+	4-[4-[(8S,9	5,414	ChEBI=CH
ID00187_2	Unknown	0	1417	4,852,913	[M+H]+	3-[4-[(8R,9	5,414	ChEBI=CH
ID00187_2	Unknown	0	1417	4,852,913	[M+H]+	4-[4-[(8S,9	5,414	ChEBI=CH

ID00187_2	Unknown	0	1417	4,852,913	[M+H]+	Commune	5,362	KNApSAcK
ID00187_2	Unknown	0	1417	4,852,913	[M+H]+		-1	
ID00187_2	Unknown	0	1417	4,852,913	[M+H]+		-1	
ID00188_1	Unknown	0	1545	4,970,421	[M+H]+		-1	
ID00188_1	Unknown	0	1545	4,970,421	[M+H]+		-1	
ID00188_1	Unknown	0	1545	4,970,421	[M+H]+		-1	
ID00188_1	Unknown	0	1545	4,970,421	[M+H]+		-1	
ID00188_1	Unknown	0	1545	4,970,421	[M+H]+		-1	
ID00189_2	LPE 20:4	0	1493	5,022,932	[M+H]+	Fexofenadi	57,121	HMDB=HM
ID00189_2	LPE 20:4	0	1493	5,022,932	[M+H]+	LysoPE(20:	56,105	HMDB=HM
ID00189_2	LPE 20:4	0	1493	5,022,932	[M+H]+	LysoPE(0:0	56,007	HMDB=HM
ID00189_2	LPE 20:4	0	1493	5,022,932	[M+H]+	Aflatrem;al	5,564	KNApSAcK
ID00189_2	LPE 20:4	0	1493	5,022,932	[M+H]+	LysoPE(20:	55,353	HMDB=HM
ID00189_2	LPE 20:4	0	1493	5,022,932	[M+H]+	LysoPE(0:0	55,289	HMDB=HM
ID00189_2	LPE 20:4	0	1493	5,022,932	[M+H]+	(2R,4bS,6a	55,046	NPA=NPA0
ID00189_2	LPE 20:4	0	1493	5,022,932	[M+H]+	Pyrrospiron	54,266	NPA=NPA0
ID00189_2	LPE 20:4	0	1493	5,022,932	[M+H]+	Paspalitrem	54,266	KNApSAcK
ID00189_2	LPE 20:4	0	1493	5,022,932	[M+H]+		-1	
ID00190_1	w/o MS2:SL	0	474	502,316	[M+H]+	Delaminom	5,194	NPA=NPA0
ID00190_1	w/o MS2:SL	0	474	502,316	[M+H]+		-1	
ID00190_1	w/o MS2:SL	0	474	502,316	[M+H]+		-1	
ID00190_1	w/o MS2:SL	0	474	502,316	[M+H]+		-1	
ID00190_1	w/o MS2:SL	0	474	502,316	[M+H]+		-1	
ID00191_2	Unknown	0	966	5,023,372	[M+H]+		-1	
ID00191_2	Unknown	0	966	5,023,372	[M+H]+		-1	
ID00191_2	Unknown	0	966	5,023,372	[M+H]+		-1	
ID00191_2	Unknown	0	966	5,023,372	[M+H]+		-1	
ID00191_2	Unknown	0	966	5,023,372	[M+H]+		-1	
ID00192_1	Unknown	0	523	5,098,862	[M+2H]2+		-1	
ID00192_1	Unknown	0	523	5,098,862	[M+2H]2+		-1	
ID00192_1	Unknown	0	523	5,098,862	[M+2H]2+		-1	
ID00192_1	Unknown	0	523	5,098,862	[M+2H]2+		-1	
ID00192_1	Unknown	0	523	5,098,862	[M+2H]2+		-1	
ID00193_1	Unknown	0	1059	5,098,861	[M+2H]2+		-1	
ID00193_1	Unknown	0	1059	5,098,861	[M+2H]2+		-1	
ID00193_1	Unknown	0	1059	5,098,861	[M+2H]2+		-1	
ID00193_1	Unknown	0	1059	5,098,861	[M+2H]2+		-1	
ID00193_1	Unknown	0	1059	5,098,861	[M+2H]2+		-1	
ID00194_0	Unknown	0	533	5,098,863	[M+2H]2+		-1	
ID00194_0	Unknown	0	533	5,098,863	[M+2H]2+		-1	
ID00194_0	Unknown	0	533	5,098,863	[M+2H]2+		-1	
ID00194_0	Unknown	0	533	5,098,863	[M+2H]2+		-1	
ID00194_0	Unknown	0	533	5,098,863	[M+2H]2+		-1	
ID00195_0	DG 27:4	0	1705	5,133,636	[M+Na]+		-1	

ID00195_0	DG 27:4	0	1705	5,133,636	[M+Na]+		-1	
ID00195_0	DG 27:4	0	1705	5,133,636	[M+Na]+		-1	
ID00195_0	DG 27:4	0	1705	5,133,636	[M+Na]+		-1	
ID00195_0	DG 27:4	0	1705	5,133,636	[M+Na]+		-1	
ID00197_1	w/o MS2:SL	0	532	5,163,311	[M+Na]+	Borrelidin B	52,087	NPA=NPA0
ID00197_1	w/o MS2:SL	0	532	5,163,311	[M+Na]+		-1	
ID00197_1	w/o MS2:SL	0	532	5,163,311	[M+Na]+		-1	
ID00197_1	w/o MS2:SL	0	532	5,163,311	[M+Na]+		-1	
ID00197_1	w/o MS2:SL	0	532	5,163,311	[M+Na]+		-1	
ID00198_1	Unknown	0	760	5,188,914	[M+2H]2+		-1	
ID00198_1	Unknown	0	760	5,188,914	[M+2H]2+		-1	
ID00198_1	Unknown	0	760	5,188,914	[M+2H]2+		-1	
ID00198_1	Unknown	0	760	5,188,914	[M+2H]2+		-1	
ID00198_1	Unknown	0	760	5,188,914	[M+2H]2+		-1	
ID00199_1	Unknown	0	350	5,203,323	[M+H]+		-1	
ID00199_1	Unknown	0	350	5,203,323	[M+H]+		-1	
ID00199_1	Unknown	0	350	5,203,323	[M+H]+		-1	
ID00199_1	Unknown	0	350	5,203,323	[M+H]+		-1	
ID00199_1	Unknown	0	350	5,203,323	[M+H]+		-1	
ID00200_1	Unknown	0	1407	5,298,828	[M+2H]2+		-1	
ID00200_1	Unknown	0	1407	5,298,828	[M+2H]2+		-1	
ID00200_1	Unknown	0	1407	5,298,828	[M+2H]2+		-1	
ID00200_1	Unknown	0	1407	5,298,828	[M+2H]2+		-1	
ID00200_1	Unknown	0	1407	5,298,828	[M+2H]2+		-1	
ID00201_2	Unknown	0	1246	5,312,739	[M+H]+	glycerol ph	54,157	ChEBI=CH
ID00201_2	Unknown	0	1246	5,312,739	[M+H]+		-1	
ID00201_2	Unknown	0	1246	5,312,739	[M+H]+		-1	
ID00201_2	Unknown	0	1246	5,312,739	[M+H]+		-1	
ID00201_2	Unknown	0	1246	5,312,739	[M+H]+		-1	
ID00202_1	Unknown	0	770	5,323,289	[M+K]+		-1	
ID00202_1	Unknown	0	770	5,323,289	[M+K]+		-1	
ID00202_1	Unknown	0	770	5,323,289	[M+K]+		-1	
ID00202_1	Unknown	0	770	5,323,289	[M+K]+		-1	
ID00202_1	Unknown	0	770	5,323,289	[M+K]+		-1	
ID00205_0	Unknown	0	953	5,398,973	[M+2H]2+	UNPD2002	51,787	UNPD=UN
ID00205_0	Unknown	0	953	5,398,973	[M+2H]2+		-1	
ID00205_0	Unknown	0	953	5,398,973	[M+2H]2+		-1	
ID00205_0	Unknown	0	953	5,398,973	[M+2H]2+		-1	
ID00205_0	Unknown	0	953	5,398,973	[M+2H]2+		-1	
ID00206_1	Unknown	0	1499	5,408,719	[M+H]+		-1	
ID00206_1	Unknown	0	1499	5,408,719	[M+H]+		-1	
ID00206_1	Unknown	0	1499	5,408,719	[M+H]+		-1	
ID00206_1	Unknown	0	1499	5,408,719	[M+H]+		-1	
ID00207_2	Unknown	0	1075	5,483,033	[M+H]+		-1	

ID00207_2	Unknown	0	1075	5,483,033	[M+H]+		-1	
ID00207_2	Unknown	0	1075	5,483,033	[M+H]+		-1	
ID00207_2	Unknown	0	1075	5,483,033	[M+H]+		-1	
ID00208_1	Unknown	0	444	5,643,583	[M+H]+		-1	
ID00208_1	Unknown	0	444	5,643,583	[M+H]+		-1	
ID00208_1	Unknown	0	444	5,643,583	[M+H]+		-1	
ID00208_1	Unknown	0	444	5,643,583	[M+H]+		-1	
ID00209_1	w/o MS2:LP	0	916	5,664,274	[M+2H]2+		-1	
ID00209_1	w/o MS2:LP	0	916	5,664,274	[M+2H]2+		-1	
ID00209_1	w/o MS2:LP	0	916	5,664,274	[M+2H]2+		-1	
ID00209_1	w/o MS2:LP	0	916	5,664,274	[M+2H]2+		-1	
ID00210_1	Unknown	0	1236	5,664,296	[M+H]+	UNPD2295	48,316	UNPD=UN
ID00210_1	Unknown	0	1236	5,664,296	[M+H]+	UNPD2251	47,845	UNPD=UN
ID00210_1	Unknown	0	1236	5,664,296	[M+H]+	Clavatustid	47,845	NPA=NPA0
ID00210_1	Unknown	0	1236	5,664,296	[M+H]+		-1	
ID00210_1	Unknown	0	1236	5,664,296	[M+H]+		-1	
ID00210_1	Unknown	0	1236	5,664,296	[M+H]+		-1	
ID00211_0	w/o MS2:LP	0	536	5,664,288	[M+H]+	UNPD2295	50,283	UNPD=UN
ID00211_0	w/o MS2:LP	0	536	5,664,288	[M+H]+	Clavatustid	49,748	NPA=NPA0
ID00211_0	w/o MS2:LP	0	536	5,664,288	[M+H]+	UNPD2251	49,748	UNPD=UN
ID00211_0	w/o MS2:LP	0	536	5,664,288	[M+H]+		-1	
ID00211_0	w/o MS2:LP	0	536	5,664,288	[M+H]+		-1	
ID00211_0	w/o MS2:LP	0	536	5,664,288	[M+H]+		-1	
ID00213_1	w/o MS2:N	0	1256	5,754,343	[M+2H]2+		-1	
ID00213_1	w/o MS2:N	0	1256	5,754,343	[M+2H]2+		-1	
ID00213_1	w/o MS2:N	0	1256	5,754,343	[M+2H]2+		-1	
ID00213_1	w/o MS2:N	0	1256	5,754,343	[M+2H]2+		-1	
ID00214_1	Unknown	0	1993	5,806,036	[M+H]+	Cer(m18:0	48,492	LipidMAPS
ID00215_1	w/o MS2:V	0	1076	5,834,553	[M+H]+		-1	
ID00215_1	w/o MS2:V	0	1076	5,834,553	[M+H]+		-1	
ID00215_1	w/o MS2:V	0	1076	5,834,553	[M+H]+		-1	
ID00215_1	w/o MS2:V	0	1076	5,834,553	[M+H]+		-1	
ID00216_1	w/o MS2:C	0	1226	5,864,244	[M+2H]2+		-1	
ID00216_1	w/o MS2:C	0	1226	5,864,244	[M+2H]2+		-1	
ID00216_1	w/o MS2:C	0	1226	5,864,244	[M+2H]2+		-1	
ID00216_1	w/o MS2:C	0	1226	5,864,244	[M+2H]2+		-1	
ID00217_1	Unknown	0	1125	5,884,113	[M+Na]+	UNPD2295	50,477	UNPD=UN
ID00217_1	Unknown	0	1125	5,884,113	[M+Na]+	UNPD2251	49,709	UNPD=UN
ID00217_1	Unknown	0	1125	5,884,113	[M+Na]+	Clavatustid	49,709	NPA=NPA0
ID00217_1	Unknown	0	1125	5,884,113	[M+Na]+		-1	
ID00217_1	Unknown	0	1125	5,884,113	[M+Na]+		-1	
ID00217_1	Unknown	0	1125	5,884,113	[M+Na]+		-1	
ID00218_1	Unknown	0	592	594,161	[M+H]+	Buanmycin	51,186	NPA=NPA0
ID00218_1	Unknown	0	592	594,161	[M+H]+		-1	

ID00218_1	Unknown	0	592	594,161	[M+H]+		-1	
ID00218_1	Unknown	0	592	594,161	[M+H]+		-1	
ID00219_0	w/o MS2:N	0	1825	5,964,373	[M+2H]2+		-1	
ID00219_0	w/o MS2:N	0	1825	5,964,373	[M+2H]2+		-1	
ID00219_0	w/o MS2:N	0	1825	5,964,373	[M+2H]2+		-1	
ID00219_0	w/o MS2:N	0	1825	5,964,373	[M+2H]2+		-1	
ID00220_1	w/o MS2:RI	0	1071	597,416	[M+2H]2+		-1	
ID00220_1	w/o MS2:RI	0	1071	597,416	[M+2H]2+		-1	
ID00220_1	w/o MS2:RI	0	1071	597,416	[M+2H]2+		-1	
ID00220_1	w/o MS2:RI	0	1071	597,416	[M+2H]2+		-1	
ID00221_1	Unknown	0	1393	6,041,207	[M+H]+		-1	
ID00221_1	Unknown	0	1393	6,041,207	[M+H]+		-1	
ID00221_1	Unknown	0	1393	6,041,207	[M+H]+		-1	
ID00221_1	Unknown	0	1393	6,041,207	[M+H]+		-1	
ID00222_1	w/o MS2:SL	0	985	6,044,564	[M+H]+		-1	
ID00222_1	w/o MS2:SL	0	985	6,044,564	[M+H]+		-1	
ID00222_1	w/o MS2:SL	0	985	6,044,564	[M+H]+		-1	
ID00222_1	w/o MS2:SL	0	985	6,044,564	[M+H]+		-1	
ID00223_1	Unknown	0	318	6,083,864	[M+H]+		-1	
ID00223_1	Unknown	0	318	6,083,864	[M+H]+		-1	
ID00223_1	Unknown	0	318	6,083,864	[M+H]+		-1	
ID00223_1	Unknown	0	318	6,083,864	[M+H]+		-1	
ID00225_2	w/o MS2:SL	0	956	6,183,793	[M+H]+	Lolicine B	57,309	KNApSAcK
ID00225_2	w/o MS2:SL	0	956	6,183,793	[M+H]+	UNPD6731	57,264	UNPD=UN
ID00225_2	w/o MS2:SL	0	956	6,183,793	[M+H]+		-1	
ID00225_2	w/o MS2:SL	0	956	6,183,793	[M+H]+		-1	
ID00225_2	w/o MS2:SL	0	956	6,183,793	[M+H]+		-1	
ID00226_5	Unknown	0	1306	6,213,099	[M+H]+	(3R)-2-tert-	51,851	ChEBI=CH
ID00226_5	Unknown	0	1306	6,213,099	[M+H]+		-1	
ID00226_5	Unknown	0	1306	6,213,099	[M+H]+		-1	
ID00226_5	Unknown	0	1306	6,213,099	[M+H]+		-1	
ID00228_1	Unknown	0	903	6,229,711	[M+2H]2+		-1	
ID00228_1	Unknown	0	903	6,229,711	[M+2H]2+		-1	
ID00228_1	Unknown	0	903	6,229,711	[M+2H]2+		-1	
ID00228_1	Unknown	0	903	6,229,711	[M+2H]2+		-1	
ID00229_0	w/o MS2:SL	0	951	6,264,492	[M+H]+		-1	
ID00229_0	w/o MS2:SL	0	951	6,264,492	[M+H]+		-1	
ID00229_0	w/o MS2:SL	0	951	6,264,492	[M+H]+		-1	
ID00229_0	w/o MS2:SL	0	951	6,264,492	[M+H]+		-1	
ID00230_1	Unknown	0	1400	6,319,762	[M+2H]2+		-1	
ID00230_1	Unknown	0	1400	6,319,762	[M+2H]2+		-1	
ID00230_1	Unknown	0	1400	6,319,762	[M+2H]2+		-1	
ID00230_1	Unknown	0	1400	6,319,762	[M+2H]2+		-1	
ID00231_2	RIKEN P-VS	0	900	6,373,058	[M+H]+		-1	

ID00231_2	RIKEN P-VS	0	900	6,373,058	[M+H]+		-1	
ID00231_2	RIKEN P-VS	0	900	6,373,058	[M+H]+		-1	
ID00231_2	RIKEN P-VS	0	900	6,373,058	[M+H]+		-1	
ID00232_1	Unknown	0	778	638,288	[M+H]+		-1	
ID00232_1	Unknown	0	778	638,288	[M+H]+		-1	
ID00232_1	Unknown	0	778	638,288	[M+H]+		-1	
ID00232_1	Unknown	0	778	638,288	[M+H]+		-1	
ID00233_5	RIKEN P-VS	0	1873	6,383,375	[M+H]+		-1	
ID00233_5	RIKEN P-VS	0	1873	6,383,375	[M+H]+		-1	
ID00233_5	RIKEN P-VS	0	1873	6,383,375	[M+H]+		-1	
ID00233_5	RIKEN P-VS	0	1873	6,383,375	[M+H]+		-1	
ID00234_1	Unknown	0	1167	6,421,527	[M+K]+		-1	
ID00234_1	Unknown	0	1167	6,421,527	[M+K]+		-1	
ID00234_1	Unknown	0	1167	6,421,527	[M+K]+		-1	
ID00234_1	Unknown	0	1167	6,421,527	[M+K]+		-1	
ID00236_1	Unknown	0	646	6,523,029	[M+H]+		-1	
ID00236_1	Unknown	0	646	6,523,029	[M+H]+		-1	
ID00236_1	Unknown	0	646	6,523,029	[M+H]+		-1	
ID00236_1	Unknown	0	646	6,523,029	[M+H]+		-1	
ID00237_1	w/o MS2:B	0	556	652,412	[M+H]+		-1	
ID00237_1	w/o MS2:B	0	556	652,412	[M+H]+		-1	
ID00237_1	w/o MS2:B	0	556	652,412	[M+H]+		-1	
ID00237_1	w/o MS2:B	0	556	652,412	[M+H]+		-1	
ID00239_2	RIKEN P-VS	0	1179	6,543,331	[M+H]+		-1	
ID00239_2	RIKEN P-VS	0	1179	6,543,331	[M+H]+		-1	
ID00239_2	RIKEN P-VS	0	1179	6,543,331	[M+H]+		-1	
ID00239_2	RIKEN P-VS	0	1179	6,543,331	[M+H]+		-1	
ID00240_1	TG 36:0|TG	0	2151	6,565,834	[M+NH4]+	TG(12:0/12	55,779	HMDB=HM
ID00240_1	TG 36:0|TG	0	2151	6,565,834	[M+NH4]+	TG(10:0/a-	53,477	HMDB=HM
ID00240_1	TG 36:0|TG	0	2151	6,565,834	[M+NH4]+	TG(10:0/i-1	53,477	HMDB=HM
ID00240_1	TG 36:0|TG	0	2151	6,565,834	[M+NH4]+	TG(a-13:0/	53,467	HMDB=HM
ID00240_1	TG 36:0|TG	0	2151	6,565,834	[M+NH4]+	TG(10:0/i-1	53,446	HMDB=HM
ID00240_1	TG 36:0|TG	0	2151	6,565,834	[M+NH4]+	TG(10:0/a-	53,417	HMDB=HM
ID00240_1	TG 36:0|TG	0	2151	6,565,834	[M+NH4]+	TG(i-12:0/i-	53,348	HMDB=HM
ID00240_1	TG 36:0|TG	0	2151	6,565,834	[M+NH4]+	TG(12:0/12	53,348	HMDB=HM
ID00240_1	TG 36:0|TG	0	2151	6,565,834	[M+NH4]+	TG(i-12:0/8	53,288	HMDB=HM
ID00240_1	TG 36:0|TG	0	2151	6,565,834	[M+NH4]+	TG(i-12:0/i-	53,286	HMDB=HM
ID00241_1	RIKEN P-VS	0	1512	6,634,526	[M+H]+		-1	
ID00241_1	RIKEN P-VS	0	1512	6,634,526	[M+H]+		-1	
ID00241_1	RIKEN P-VS	0	1512	6,634,526	[M+H]+		-1	
ID00241_1	RIKEN P-VS	0	1512	6,634,526	[M+H]+		-1	
ID00243_6	RIKEN P-VS	0	1582	6,756,752	[M+H]+		-1	
ID00243_6	RIKEN P-VS	0	1582	6,756,752	[M+H]+		-1	
ID00243_6	RIKEN P-VS	0	1582	6,756,752	[M+H]+		-1	

ID00243_6	RIKEN P-VS	0	1582	6,756,752	[M+H]+		-1	
ID00244_0	DG 39:5	0	629	6,795,138	[M+Na]+		-1	
ID00244_0	DG 39:5	0	629	6,795,138	[M+Na]+		-1	
ID00244_0	DG 39:5	0	629	6,795,138	[M+Na]+		-1	
ID00244_0	DG 39:5	0	629	6,795,138	[M+Na]+		-1	
ID00245_1	DG 39:5	0	432	6,795,138	[M+Na]+		-1	
ID00245_1	DG 39:5	0	432	6,795,138	[M+Na]+		-1	
ID00245_1	DG 39:5	0	432	6,795,138	[M+Na]+		-1	
ID00245_1	DG 39:5	0	432	6,795,138	[M+Na]+		-1	
ID00246_1	RIKEN P-VS	0	890	6,804,816	[M+H]+		-1	
ID00246_1	RIKEN P-VS	0	890	6,804,816	[M+H]+		-1	
ID00246_1	RIKEN P-VS	0	890	6,804,816	[M+H]+		-1	
ID00246_1	RIKEN P-VS	0	890	6,804,816	[M+H]+		-1	
ID00250_0	w/o MS2:H	0	569	6,965,364	[M+H]+		-1	
ID00250_0	w/o MS2:H	0	569	6,965,364	[M+H]+		-1	
ID00250_0	w/o MS2:H	0	569	6,965,364	[M+H]+		-1	
ID00250_0	w/o MS2:H	0	569	6,965,364	[M+H]+		-1	
ID00251_1	w/o MS2:H	0	529	6,965,381	[M+H]+		-1	
ID00251_1	w/o MS2:H	0	529	6,965,381	[M+H]+		-1	
ID00251_1	w/o MS2:H	0	529	6,965,381	[M+H]+		-1	
ID00251_1	w/o MS2:H	0	529	6,965,381	[M+H]+		-1	
ID00252_1	w/o MS2:SL	0	1028	6,975,207	[M+H]+		-1	
ID00252_1	w/o MS2:SL	0	1028	6,975,207	[M+H]+		-1	
ID00252_1	w/o MS2:SL	0	1028	6,975,207	[M+H]+		-1	
ID00252_1	w/o MS2:SL	0	1028	6,975,207	[M+H]+		-1	
ID00253_1	w/o MS2:SL	0	95	701,493	[M+Na]+	Campechic	42,738	NPA=NPA0
ID00253_1	w/o MS2:SL	0	95	701,493	[M+Na]+		-1	
ID00253_1	w/o MS2:SL	0	95	701,493	[M+Na]+		-1	
ID00253_1	w/o MS2:SL	0	95	701,493	[M+Na]+		-1	
ID00255_1	PE 34:2|PE	0	1474	7,165,226	[M+H]+	PE(16:0/18	61,129	HMDB=HM
ID00255_1	PE 34:2|PE	0	1474	7,165,226	[M+H]+	PE(18:1(9Z	60,043	HMDB=HM
ID00255_1	PE 34:2|PE	0	1474	7,165,226	[M+H]+	PE(18:2(9Z	59,715	HMDB=HM
ID00255_1	PE 34:2|PE	0	1474	7,165,226	[M+H]+	PE(14:0/20	59,486	HMDB=HM
ID00255_1	PE 34:2|PE	0	1474	7,165,226	[M+H]+	PE(20:2(11	59,415	HMDB=HM
ID00255_1	PE 34:2|PE	0	1474	7,165,226	[M+H]+	PE(20:1(11	58,758	HMDB=HM
ID00255_1	PE 34:2|PE	0	1474	7,165,226	[M+H]+	PE(14:1(9Z	58,702	HMDB=HM
ID00255_1	PE 34:2|PE	0	1474	7,165,226	[M+H]+	PE(16:1(9Z	58,628	HMDB=HM
ID00255_1	PE 34:2|PE	0	1474	7,165,226	[M+H]+	PC(13:0/18	58,298	LipidMAPS
ID00255_1	PE 34:2|PE	0	1474	7,165,226	[M+H]+	PC(18:2(9Z	58,298	LipidMAPS
ID00256_1	PE P-36:4|	0	1944	7,245,272	[M+H]+	PE(P-16:0/	60,154	HMDB=HM
ID00256_1	PE P-36:4|	0	1944	7,245,272	[M+H]+	PE(P-18:0/	58,441	HMDB=HM
ID00256_1	PE P-36:4|	0	1944	7,245,272	[M+H]+	PE(O-16:0/	57,481	LipidMAPS
ID00256_1	PE P-36:4|	0	1944	7,245,272	[M+H]+	PE(20:4(5Z	57,214	HMDB=HM
ID00256_1	PE P-36:4|	0	1944	7,245,272	[M+H]+	PE(18:4(6Z	56,934	HMDB=HM

ID00256_1 PE P-36:4|	0	1944	7,245,272	[M+H]+	PE(P-16:0/	56,864	HMDB=HM
ID00256_1 PE P-36:4|	0	1944	7,245,272	[M+H]+	PE(P-18:1(	56,767	HMDB=HM
ID00256_1 PE P-36:4|	0	1944	7,245,272	[M+H]+	PE(P-18:1(	56,755	HMDB=HM
ID00256_1 PE P-36:4|	0	1944	7,245,272	[M+H]+	PE(20:4(8Z	56,689	HMDB=HM
ID00256_1 PE P-36:4|	0	1944	7,245,272	[M+H]+	PE(18:3(6Z	56,618	HMDB=HM
ID00258_1 Unknown	0	989	7,360,544	[M+2H]2+		-1	
ID00258_1 Unknown	0	989	7,360,544	[M+2H]2+		-1	
ID00258_1 Unknown	0	989	7,360,544	[M+2H]2+		-1	
ID00258_1 Unknown	0	989	7,360,544	[M+2H]2+		-1	
ID00260_2 w/o MS2:P	0	537	7,384,274	[M+H]+		-1	
ID00260_2 w/o MS2:P	0	537	7,384,274	[M+H]+		-1	
ID00260_2 w/o MS2:P	0	537	7,384,274	[M+H]+		-1	
ID00260_2 w/o MS2:P	0	537	7,384,274	[M+H]+		-1	
ID00261_0 DG 44:10	0	866	739,531	[M+Na]+		-1	
ID00261_0 DG 44:10	0	866	739,531	[M+Na]+		-1	
ID00261_0 DG 44:10	0	866	739,531	[M+Na]+		-1	
ID00261_0 DG 44:10	0	866	739,531	[M+Na]+		-1	
ID00262_9 PE 36:4|PE	0	1665	7,405,212	[M+H]+	PE(16:0/20	60,051	HMDB=HM
ID00262_9 PE 36:4|PE	0	1665	7,405,212	[M+H]+	PC(15:0/18	58,932	HMDB=HM
ID00262_9 PE 36:4|PE	0	1665	7,405,212	[M+H]+	PC(18:4(6Z	58,901	HMDB=HM
ID00262_9 PE 36:4|PE	0	1665	7,405,212	[M+H]+	PE(20:4(5Z	58,614	HMDB=HM
ID00262_9 PE 36:4|PE	0	1665	7,405,212	[M+H]+	PE(18:2(9Z	58,424	HMDB=HM
ID00262_9 PE 36:4|PE	0	1665	7,405,212	[M+H]+	PE(18:0/18	58,383	HMDB=HM
ID00262_9 PE 36:4|PE	0	1665	7,405,212	[M+H]+	PE(18:4(6Z	58,355	HMDB=HM
ID00262_9 PE 36:4|PE	0	1665	7,405,212	[M+H]+	PE(18:1(9Z	58,205	HMDB=HM
ID00262_9 PE 36:4|PE	0	1665	7,405,212	[M+H]+	PE(18:3(6Z	58,175	HMDB=HM
ID00262_9 PE 36:4|PE	0	1665	7,405,212	[M+H]+	PE(14:0/22	58,065	HMDB=HM
ID00263_1 PE 36:3|PE	0	1241	7,425,372	[M+H]+	PE(16:0/20	61,054	HMDB=HM
ID00263_1 PE 36:3|PE	0	1241	7,425,372	[M+H]+	PE(18:1(9Z6,03		HMDB=HM
ID00263_1 PE 36:3|PE	0	1241	7,425,372	[M+H]+	PC(18:3(6Z	60,208	HMDB=HM
ID00263_1 PE 36:3|PE	0	1241	7,425,372	[M+H]+	PC(15:0/18	60,208	HMDB=HM
ID00263_1 PE 36:3|PE	0	1241	7,425,372	[M+H]+	PE(18:3(6Z	60,134	HMDB=HM
ID00263_1 PE 36:3|PE	0	1241	7,425,372	[M+H]+	PE(18:0/18	60,134	HMDB=HM
ID00263_1 PE 36:3|PE	0	1241	7,425,372	[M+H]+	PC(18:3(9Z	60,043	HMDB=HM
ID00263_1 PE 36:3|PE	0	1241	7,425,372	[M+H]+	PC(15:0/18	60,043	HMDB=HM
ID00263_1 PE 36:3|PE	0	1241	7,425,372	[M+H]+	PE(18:0/18	59,757	HMDB=HM
ID00263_1 PE 36:3|PE	0	1241	7,425,372	[M+H]+	PE(18:3(9Z	59,755	HMDB=HM
ID00264_9 PE P-38:6|	0	1866	748,527	[M+H]+	PE(O-16:1(	58,224	HMDB=HM
ID00264_9 PE P-38:6|	0	1866	748,527	[M+H]+	PE(P-18:1(	57,066	HMDB=HM
ID00264_9 PE P-38:6|	0	1866	748,527	[M+H]+	PE(P-18:1(	55,669	HMDB=HM
ID00264_9 PE P-38:6|	0	1866	748,527	[M+H]+	PE(20:5(5Z	5,551	HMDB=HM
ID00264_9 PE P-38:6|	0	1866	748,527	[M+H]+	PE(20:5(5Z	55,507	HMDB=HM
ID00264_9 PE P-38:6|	0	1866	748,527	[M+H]+	PE(22:6(4Z	5,533	HMDB=HM
ID00264_9 PE P-38:6|	0	1866	748,527	[M+H]+		-1	

ID00264_9	PE P-38:6|	0	1866	748,527	[M+H]+		-1	
ID00264_9	PE P-38:6|	0	1866	748,527	[M+H]+		-1	
ID00266_1	w/o MS2:H	0	679	7,505,573	[M+H]+		-1	
ID00266_1	w/o MS2:H	0	679	7,505,573	[M+H]+		-1	
ID00266_1	w/o MS2:H	0	679	7,505,573	[M+H]+		-1	
ID00266_1	w/o MS2:H	0	679	7,505,573	[M+H]+		-1	
ID00267_9	PE 38:6|PE	0	1754	7,645,236	[M+H]+	PE(16:0/22	60,563	HMDB=HM
ID00267_9	PE 38:6|PE	0	1754	7,645,236	[M+H]+	PE(22:6(4Z	59,174	HMDB=HM
ID00267_9	PE 38:6|PE	0	1754	7,645,236	[M+H]+	PE(20:5(5Z	59,169	HMDB=HM
ID00267_9	PE 38:6|PE	0	1754	7,645,236	[M+H]+	PE(18:1(9Z	5,916	HMDB=HM
ID00267_9	PE 38:6|PE	0	1754	7,645,236	[M+H]+	PE(20:4(5Z	58,993	HMDB=HM
ID00267_9	PE 38:6|PE	0	1754	7,645,236	[M+H]+	PE(18:2(9Z	58,984	HMDB=HM
ID00267_9	PE 38:6|PE	0	1754	7,645,236	[M+H]+	PE(18:4(6Z	58,751	HMDB=HM
ID00267_9	PE 38:6|PE	0	1754	7,645,236	[M+H]+	PE(20:2(11	58,746	HMDB=HM
ID00267_9	PE 38:6|PE	0	1754	7,645,236	[M+H]+	PC(22:6(4Z	58,481	LipidMAPS
ID00267_9	PE 38:6|PE	0	1754	7,645,236	[M+H]+	PC(13:0/22	5,848	LipidMAPS
ID00268_1	w/o MS2:T	0	602	7,645,748	[M+Na]+		-1	
ID00268_1	w/o MS2:T	0	602	7,645,748	[M+Na]+		-1	
ID00268_1	w/o MS2:T	0	602	7,645,748	[M+Na]+		-1	
ID00268_1	w/o MS2:T	0	602	7,645,748	[M+Na]+		-1	
ID00269_9	PE 38:5|PE	0	1694	766,538	[M+H]+	PE(18:1(9Z	59,221	HMDB=HM
ID00269_9	PE 38:5|PE	0	1694	766,538	[M+H]+	PC(20:5(5Z	5,856	HMDB=HM
ID00269_9	PE 38:5|PE	0	1694	766,538	[M+H]+	PC(15:0/20	58,559	HMDB=HM
ID00269_9	PE 38:5|PE	0	1694	766,538	[M+H]+	PE(20:4(5Z	57,908	HMDB=HM
ID00269_9	PE 38:5|PE	0	1694	766,538	[M+H]+	PE(18:0/20	57,864	HMDB=HM
ID00269_9	PE 38:5|PE	0	1694	766,538	[M+H]+	PE(20:5(5Z	57,836	HMDB=HM
ID00269_9	PE 38:5|PE	0	1694	766,538	[M+H]+	PE(20:1(11	57,678	HMDB=HM
ID00269_9	PE 38:5|PE	0	1694	766,538	[M+H]+	PE(18:4(6Z	57,668	HMDB=HM
ID00269_9	PE 38:5|PE	0	1694	766,538	[M+H]+	PE(20:2(11	57,467	HMDB=HM
ID00269_9	PE 38:5|PE	0	1694	766,538	[M+H]+	PE(18:3(6Z	57,457	HMDB=HM
ID00270_1	PE 38:4|PE	0	2058	7,685,543	[M+H]+	PE(18:0/20	62,033	HMDB=HM
ID00270_1	PE 38:4|PE	0	2058	7,685,543	[M+H]+	PC(15:0/20	60,896	HMDB=HM
ID00270_1	PE 38:4|PE	0	2058	7,685,543	[M+H]+	PE(16:0/22	60,047	HMDB=HM
ID00270_1	PE 38:4|PE	0	2058	7,685,543	[M+H]+	PC(20:4(5Z	59,509	HMDB=HM
ID00270_1	PE 38:4|PE	0	2058	7,685,543	[M+H]+	PE(20:4(5Z	5,925	HMDB=HM
ID00270_1	PE 38:4|PE	0	2058	7,685,543	[M+H]+	PE(20:0/18	59,048	HMDB=HM
ID00270_1	PE 38:4|PE	0	2058	7,685,543	[M+H]+	PE(18:4(6Z	59,035	HMDB=HM
ID00270_1	PE 38:4|PE	0	2058	7,685,543	[M+H]+	PE(18:1(9Z	59,019	HMDB=HM
ID00270_1	PE 38:4|PE	0	2058	7,685,543	[M+H]+	PE(18:3(6Z	58,807	HMDB=HM
ID00270_1	PE 38:4|PE	0	2058	7,685,543	[M+H]+	PE(20:1(11	58,805	HMDB=HM
ID00273_1	w/o MS2:H	0	760	7,805,682	[M+K]+		-1	
ID00273_1	w/o MS2:H	0	760	7,805,682	[M+K]+		-1	
ID00273_1	w/o MS2:H	0	760	7,805,682	[M+K]+		-1	
ID00273_1	w/o MS2:H	0	760	7,805,682	[M+K]+		-1	

ID00275_1 w/o MS2:LP	0	413	792,598	[M+2H]2+		-1	
ID00275_1 w/o MS2:LP	0	413	792,598	[M+2H]2+		-1	
ID00275_1 w/o MS2:LP	0	413	792,598	[M+2H]2+		-1	
ID00275_1 w/o MS2:LP	0	413	792,598	[M+2H]2+		-1	
ID00276_0 w/o MS2:LP	0	473	7,925,981	[M+H]+		-1	
ID00276_0 w/o MS2:LP	0	473	7,925,981	[M+H]+		-1	
ID00276_0 w/o MS2:LP	0	473	7,925,981	[M+H]+		-1	
ID00276_0 w/o MS2:LP	0	473	7,925,981	[M+H]+		-1	
ID00280_0 w/o MS2:S	0	489	8,096,212	[M+K]+		-1	
ID00280_0 w/o MS2:S	0	489	8,096,212	[M+K]+		-1	
ID00280_0 w/o MS2:S	0	489	8,096,212	[M+K]+		-1	
ID00280_0 w/o MS2:S	0	489	8,096,212	[M+K]+		-1	
ID00281_1 w/o MS2:S	0	551	809,624	[M+H]+		-1	
ID00281_1 w/o MS2:S	0	551	809,624	[M+H]+		-1	
ID00281_1 w/o MS2:S	0	551	809,624	[M+H]+		-1	
ID00281_1 w/o MS2:S	0	551	809,624	[M+H]+		-1	
ID00282_1 w/o MS2:P	0	144	8,145,768	[M+H]+	1-(8-[5]-lad	4,498	LipidMAPS
ID00282_1 w/o MS2:P	0	144	8,145,768	[M+H]+		-1	
ID00282_1 w/o MS2:P	0	144	8,145,768	[M+H]+		-1	
ID00282_1 w/o MS2:P	0	144	8,145,768	[M+H]+		-1	
ID00283_1 TG 48:2|TG	0	1751	8,207,395	[M+NH4]+	TG(16:0/16	59,517	HMDB=HM
ID00283_1 TG 48:2|TG	0	1751	8,207,395	[M+NH4]+	TG(16:0/14	59,107	HMDB=HM
ID00283_1 TG 48:2|TG	0	1751	8,207,395	[M+NH4]+	15-Palmito	58,967	HMDB=HM
ID00283_1 TG 48:2|TG	0	1751	8,207,395	[M+NH4]+	TG(16:1(9Z	58,366	HMDB=HM
ID00283_1 TG 48:2|TG	0	1751	8,207,395	[M+NH4]+	TG(16:1(9Z	58,349	HMDB=HM
ID00283_1 TG 48:2|TG	0	1751	8,207,395	[M+NH4]+	TG(14:0/16	57,753	HMDB=HM
ID00283_1 TG 48:2|TG	0	1751	8,207,395	[M+NH4]+	TG(15:0/15	5,764	HMDB=HM
ID00283_1 TG 48:2|TG	0	1751	8,207,395	[M+NH4]+	TG(14:0/14	5,739	HMDB=HM
ID00283_1 TG 48:2|TG	0	1751	8,207,395	[M+NH4]+	TG(14:0/16	5,698	HMDB=HM
ID00283_1 TG 48:2|TG	0	1751	8,207,395	[M+NH4]+	TG(14:1(9Z	56,957	HMDB=HM
ID00284_1 TG 48:1|TG	0	1669	8,227,556	[M+NH4]+	TG(16:0/16	59,282	HMDB=HM
ID00284_1 TG 48:1|TG	0	1669	8,227,556	[M+NH4]+	TG(14:0/16	58,155	HMDB=HM
ID00284_1 TG 48:1|TG	0	1669	8,227,556	[M+NH4]+	TG(16:0/14	58,126	HMDB=HM
ID00284_1 TG 48:1|TG	0	1669	8,227,556	[M+NH4]+	TG(18:0/14	58,081	HMDB=HM
ID00284_1 TG 48:1|TG	0	1669	8,227,556	[M+NH4]+	TG(14:0/16	56,724	HMDB=HM
ID00284_1 TG 48:1|TG	0	1669	8,227,556	[M+NH4]+	TG(15:0/15	56,645	HMDB=HM
ID00284_1 TG 48:1|TG	0	1669	8,227,556	[M+NH4]+	TG(15:0/18	56,645	HMDB=HM
ID00284_1 TG 48:1|TG	0	1669	8,227,556	[M+NH4]+	TG(18:0/16	56,592	HMDB=HM
ID00284_1 TG 48:1|TG	0	1669	8,227,556	[M+NH4]+	TG(14:0/14	56,559	HMDB=HM
ID00284_1 TG 48:1|TG	0	1669	8,227,556	[M+NH4]+	TG(14:0/14	56,442	HMDB=HM
ID00285_1 TG 48:0|TG	0	1279	8,247,689	[M+NH4]+	TG(16:0/16	56,928	HMDB=HM
ID00285_1 TG 48:0|TG	0	1279	8,247,689	[M+NH4]+	TG(14:0/16	55,966	HMDB=HM
ID00285_1 TG 48:0|TG	0	1279	8,247,689	[M+NH4]+	TG(13:0/17	5,462	HMDB=HM
ID00285_1 TG 48:0|TG	0	1279	8,247,689	[M+NH4]+	TG(14:0/17	54,614	HMDB=HM

ID00285_1	TG 48:0|TG	0	1279	8,247,689	[M+NH4]+	TG(20:0/15	54,603 HMDB=HM
ID00285_1	TG 48:0|TG	0	1279	8,247,689	[M+NH4]+	TG(13:0/16	54,564 HMDB=HM
ID00285_1	TG 48:0|TG	0	1279	8,247,689	[M+NH4]+	TG(21:0/15	54,547 HMDB=HM
ID00285_1	TG 48:0|TG	0	1279	8,247,689	[M+NH4]+	TG(12:0/17	54,533 HMDB=HM
ID00285_1	TG 48:0|TG	0	1279	8,247,689	[M+NH4]+	TG(21:0/14	54,512 HMDB=HM
ID00285_1	TG 48:0|TG	0	1279	8,247,689	[M+NH4]+	TG(15:0/16	54,509 HMDB=HM
ID00287_1	TG 50:2|TG	0	1664	8,487,701	[M+NH4]+	TG(16:0/16	61,973 HMDB=HM
ID00287_1	TG 50:2|TG	0	1664	8,487,701	[M+NH4]+	TG(16:0/16	61,069 HMDB=HM
ID00287_1	TG 50:2|TG	0	1664	8,487,701	[M+NH4]+	TG(16:1(9Z	60,984 HMDB=HM
ID00287_1	TG 50:2|TG	0	1664	8,487,701	[M+NH4]+	TG(18:0/14	60,601 HMDB=HM
ID00287_1	TG 50:2|TG	0	1664	8,487,701	[M+NH4]+	TG(16:1(9Z	59,679 HMDB=HM
ID00287_1	TG 50:2|TG	0	1664	8,487,701	[M+NH4]+	Glycerol 1,	59,643 HMDB=HM
ID00287_1	TG 50:2|TG	0	1664	8,487,701	[M+NH4]+	TG(18:1(11	59,623 HMDB=HM
ID00287_1	TG 50:2|TG	0	1664	8,487,701	[M+NH4]+	TG(18:1(9Z	59,617 HMDB=HM
ID00287_1	TG 50:2|TG	0	1664	8,487,701	[M+NH4]+	TG(18:1(11	59,554 HMDB=HM
ID00287_1	TG 50:2|TG	0	1664	8,487,701	[M+NH4]+	TG(14:0/18	59,234 HMDB=HM
ID00288_1	TG 50:1|TG	0	2097	8,507,861	[M+NH4]+	TG(16:0/16	61,251 HMDB=HM
ID00288_1	TG 50:1|TG	0	2097	8,507,861	[M+NH4]+	TG(16:0/16	61,213 HMDB=HM
ID00288_1	TG 50:1|TG	0	2097	8,507,861	[M+NH4]+	TG(16:0/16	61,067 HMDB=HM
ID00288_1	TG 50:1|TG	0	2097	8,507,861	[M+NH4]+	TG(18:0/14	59,806 HMDB=HM
ID00288_1	TG 50:1|TG	0	2097	8,507,861	[M+NH4]+	TG(18:0/14	59,622 HMDB=HM
ID00288_1	TG 50:1|TG	0	2097	8,507,861	[M+NH4]+	Glycerol 1,	58,473 HMDB=HM
ID00288_1	TG 50:1|TG	0	2097	8,507,861	[M+NH4]+	TG(14:0/16	58,454 HMDB=HM
ID00288_1	TG 50:1|TG	0	2097	8,507,861	[M+NH4]+	TG(14:0/18	58,448 HMDB=HM
ID00288_1	TG 50:1|TG	0	2097	8,507,861	[M+NH4]+	TG(20:0/16	5,834 HMDB=HM
ID00288_1	TG 50:1|TG	0	2097	8,507,861	[M+NH4]+	TG(18:0/18	58,266 HMDB=HM
ID00289_1	Unknown	0	404	8,513,978	[M+H]+		-1
ID00289_1	Unknown	0	404	8,513,978	[M+H]+		-1
ID00289_1	Unknown	0	404	8,513,978	[M+H]+		-1
ID00289_1	Unknown	0	404	8,513,978	[M+H]+		-1
ID00292_1	TG 52:2|TG	0	2086	8,768,024	[M+NH4]+	TG(16:0/18	60,377 HMDB=HM
ID00292_1	TG 52:2|TG	0	2086	8,768,024	[M+NH4]+	TG(16:1(9Z	59,489 HMDB=HM
ID00292_1	TG 52:2|TG	0	2086	8,768,024	[M+NH4]+	TG(16:0/18	5,948 HMDB=HM
ID00292_1	TG 52:2|TG	0	2086	8,768,024	[M+NH4]+	TG(16:0/16	59,451 HMDB=HM
ID00292_1	TG 52:2|TG	0	2086	8,768,024	[M+NH4]+	TG(16:1(9Z	59,394 HMDB=HM
ID00292_1	TG 52:2|TG	0	2086	8,768,024	[M+NH4]+	TG(16:1(9Z 5,81	HMDB=HM
ID00292_1	TG 52:2|TG	0	2086	8,768,024	[M+NH4]+	TG(18:1(11	58,048 HMDB=HM
ID00292_1	TG 52:2|TG	0	2086	8,768,024	[M+NH4]+	TG(18:1(11	58,045 HMDB=HM
ID00292_1	TG 52:2|TG	0	2086	8,768,024	[M+NH4]+	TG(16:0/16	57,526 HMDB=HM
ID00292_1	TG 52:2|TG	0	2086	8,768,024	[M+NH4]+	TG(14:0/18	57,487 HMDB=HM
ID00293_1	TG 52:1|TG	0	1499	8,788,193	[M+NH4]+	TG(16:0/18	56,566 HMDB=HM
ID00293_1	TG 52:1|TG	0	1499	8,788,193	[M+NH4]+	TG(16:1(9Z	56,502 HMDB=HM
ID00293_1	TG 52:1|TG	0	1499	8,788,193	[M+NH4]+	TG(16:0/16	5,649 HMDB=HM
ID00293_1	TG 52:1|TG	0	1499	8,788,193	[M+NH4]+	TG(16:0/16	56,305 HMDB=HM

ID00293_1	TG 52:1|TG	0	1499	8,788,193	[M+NH4]+	TG(18:0/16	54,975	HMDB=HM
ID00293_1	TG 52:1|TG	0	1499	8,788,193	[M+NH4]+	TG(16:0/18	53,788	HMDB=HM
ID00293_1	TG 52:1|TG	0	1499	8,788,193	[M+NH4]+	TG(14:0/18	53,741	HMDB=HM
ID00293_1	TG 52:1|TG	0	1499	8,788,193	[M+NH4]+	TG(20:0/18	53,674	HMDB=HM
ID00293_1	TG 52:1|TG	0	1499	8,788,193	[M+NH4]+	TG(14:0/18	53,583	HMDB=HM
ID00293_1	TG 52:1|TG	0	1499	8,788,193	[M+NH4]+	TG(14:0/16	53,502	HMDB=HM
ID00294_1	TG 54:3|TG	0	1570	9,028,134	[M+NH4]+	TG(16:0/18	59,311	HMDB=HM
ID00294_1	TG 54:3|TG	0	1570	9,028,134	[M+NH4]+	TG(16:1(9Z	59,226	HMDB=HM
ID00294_1	TG 54:3|TG	0	1570	9,028,134	[M+NH4]+	TG(18:0/18	59,118	HMDB=HM
ID00294_1	TG 54:3|TG	0	1570	9,028,134	[M+NH4]+	TG(16:1(9Z	57,864	HMDB=HM
ID00294_1	TG 54:3|TG	0	1570	9,028,134	[M+NH4]+	TG(18:1(9Z	57,843	HMDB=HM
ID00294_1	TG 54:3|TG	0	1570	9,028,134	[M+NH4]+	TG(18:1(11	57,742	HMDB=HM
ID00294_1	TG 54:3|TG	0	1570	9,028,134	[M+NH4]+	TG(16:0/18	57,385	HMDB=HM
ID00294_1	TG 54:3|TG	0	1570	9,028,134	[M+NH4]+	TG(18:0/18	5,724	HMDB=HM
ID00294_1	TG 54:3|TG	0	1570	9,028,134	[M+NH4]+	TG(16:0/18	57,192	HMDB=HM
ID00294_1	TG 54:3|TG	0	1570	9,028,134	[M+NH4]+	TG(16:0/18	57,131	HMDB=HM
ID00295_0	w/o MS2:T	0	528	9,056,809	[M+H]+		-1	
ID00295_0	w/o MS2:T	0	528	9,056,809	[M+H]+		-1	
ID00295_0	w/o MS2:T	0	528	9,056,809	[M+H]+		-1	
ID00295_0	w/o MS2:T	0	528	9,056,809	[M+H]+		-1	
ID00296_1	w/o MS2:D	0	562	9,056,814	[M+H]+		-1	
ID00296_1	w/o MS2:D	0	562	9,056,814	[M+H]+		-1	
ID00296_1	w/o MS2:D	0	562	9,056,814	[M+H]+		-1	
ID00296_1	w/o MS2:D	0	562	9,056,814	[M+H]+		-1	
ID00297_1	Unknown	0	366	9,276,634	[M+Na]+		-1	
ID00297_1	Unknown	0	366	9,276,634	[M+Na]+		-1	
ID00297_1	Unknown	0	366	9,276,634	[M+Na]+		-1	
ID00297_1	Unknown	0	366	9,276,634	[M+Na]+		-1	

Formula	Ontology	InChIKey	SMILES
C6H11NO	Caprolacta	JBKVHLHD	O=C1NCCCCC1
C6H11NO	Cyclopropa	DVQLGAFY	O=C(N(C)C)C1CC1
C6H11NO	Trialkylami	RHDNNKQ	O=CC1N(C1)C(C)C
C6H11NO	Pyrrolidine	FYCFJMPA	O=C(C)C1NCCC1
C6H11NO	Piperidines	FEWLNYSY	O=CN1CCCCC1
C6H11NO	Oxazolines	YFSGRMON	N1=C(C)C(OC1C)C
C7H17N	Dialkylami	SQGSVBHT	N(CCC)CC(C)C
C7H17N	Trialkylami	PQZTVWVY	N(CC)(CC)CCC
C7H17N	Monoalkyla	FATQVQVM	NC(C(C)C)C(C)C
C7H17N	Monoalkyla	YAHRDLIC	NC(C)CC(C)CC
C7H17N	Monoalkyla	VSRBKQFN	NC(C)CCCCC
C7H17N	Trialkylami	PQZTVWVY	N(CC)(CC)CCC
C7H17N	Dialkylami	SQGSVBHT	N(CCC)CC(C)C
C7H17N	Monoalkyla	FATQVQVM	NC(C(C)C)C(C)C
C7H17N	Monoalkyla	VSRBKQFN	NC(C)CCCCC
C7H17N	Monoalkyla	YAHRDLIC	NC(C)CC(C)CC
C7H17N	Dialkylami	SQGSVBHT	N(CCC)CC(C)C
C7H17N	Monoalkyla	FATQVQVM	NC(C(C)C)C(C)C
C7H17N	Trialkylami	PQZTVWVY	N(CC)(CC)CCC
C7H17N	Monoalkyla	YAHRDLIC	NC(C)CC(C)CC
C7H17N	Monoalkyla	VSRBKQFN	NC(C)CCCCC
C5H11NO2	Valine and	KZSNJWFQ	O=C(O)C(N)C(C)C
C5H11NO2	Alpha amin	KWIUHFFT	O=C([O-])C[N+](C)(C)C
C5H11NO2	Delta amin	JJMDCOVW	O=C(O)CCCCN
C5H11NO2	L-alpha-am	SNDPXSYF	O=C(O)C(N)CCC
C5H11NO2	Trialkylami	JMOXSQYG	O=C(O)CCN(C)C
C5H11NO2	Alpha amin	DLAMVQG	O=C(O)C(NC)(C)C
C5H11NO2	Alanine an	QCYOIFVB	O=C(O)C(N(C)C)C
C5H11NO2	Gamma am	ABSTXSZPG	O=C(O)CCC(N)C
C5H11NO2	Gamma am	CZGLBWZX	O=C(O)CC(C)CN
C5H11NO2	Organic O-	CSDTZUBP	O=NOCCCCC
C6H15NO	1,2-aminoa	BFSVOASY	OCCN(CC)CC
C6H15NO	Trialkyl ami	LFMTUFVY	[O-][N+](CC)(CC)CC
C6H15NO	1,2-aminoa	BFSVOASY	OCCN(CC)CC
C6H15NO	Trialkyl ami	LFMTUFVY	[O-][N+](CC)(CC)CC
C6H15NO	1,2-aminoa	BFSVOASY	OCCN(CC)CC
C6H15NO	Trialkyl ami	LFMTUFVY	[O-][N+](CC)(CC)CC
C8H11N	Phenethyla	BHHGXPLM	NCCC=1C=CC=CC1
C8H11N	Aralkylami	RQEUFEKY	NC(C=1C=CC=CC1)C
C8H11N	m-Xylenes	UFFBMTHB	NC=1C(=CC=CC1C)C
C8H11N	Dialkylaryla	JLTDJTHDQ	C=1C=CC(=CC1)N(C)C
C8H11N	Phenylalkyl	OJGMBLNI	C=1C=CC(=CC1)NCC
C8H11N	Methylpyrid	COHDGTR	N=1C=C(C=CC1CC)C

C8H11N	Methylpyrid	NTSLROIKF	N=1C=C(C=CC1C)CC
C8H11N	Pyridines a	OIALIKXML	N=1C=CC=CC1CCC
C8H11N	m-Xylenes	CZZZABOK	NC1=CC=C(C=C1C)C
C8H11N	o-Xylenes	DOLQYFPD	NC1=CC=C(C(=C1)C)C
C6H6N2O	Nicotinami	DFPAKSUC	O=C(N)C=1C=NC=CC1
C6H6N2O	Pyridines a	YBKOPFQC	ON=CC=1C=NC=CC1
C6H6N2O	N-substitut	UFGLSUVQ	N=1C=CN2C1OC=CC2
C6H6N2O	Pyridinecar	IBBMAWUL	O=C(N)C1=NC=CC=C1
C6H6N2O	Pyridinecar	VFQXVTOD	O=C(N)C=1C=CN=CC1
C6H6N2O	Aryl alkyl ke	DBZAKQW	O=C(C1=NC=CN=C1)C
C2H7NO3S	Organosulf	XOAAWQZ	O=S(=O)(O)CCN
C2H7NO3S	Organic sul	JKQMHZKU	O=S(=O)(OC)NC
C8H19N	Monoalkyla	QNIVIMYXG	NC(C)CCCC(C)C
C8H19N	Monoalkyla	IOQPZZOE	NCCCCCCCC
C4H9N3O2	Alpha amin	CVSVTCOR	O=C(O)CN(C(=N)N)C
C4H9N3O2	Guanidines	KMXXSJLYV	O=C(O)CCNC(=N)N
C4H9N3O2	Alpha amin	WXOFJMW	O=C([O-])C[N+](=C(N)N)C
C4H9N3O2	Azoxy com	HQAXXRW	O=C(N=[N+]([O-])C)N(C)C
C5H4N4O	Hypoxanthi	FDGQSTZJ	OC1=NC=NC=2N=CNC12
C5H4N4O	Pyrazolo[3,	OFCNXPDA	O=C1N=CN=C2NNC=C12
C5H4N4O	Pyrazolopy	OGCXIHW	OC1=NC=NC2=C1N=NC2
C5H4N4O	Purinones	YPDSIEMYV	O=C1NC=2N=CN=CC2N1
C5H4N4O	Carbonylim	AXEBUIBRU	N#CC=1N=CN(C=O)C1N
C5H4N4O	Pyrazolopy	JFZSDNLQ	O=C1N=CNC=2C=NNC12
C5H12S2	Sulfenyl co	DPLYGYOS	SSCCCCC
C5H12S2	Dialkyldisu	PUUCPZKS	S(SC(C)C)CC
C5H12S2	Dialkyldisu	SNGRPWP	S(SCCC)CC
C3H10NO4	Phosphoet	HZDCAHRL	O=P(O)(O)OCCNC
C3H10NO4	Phosphoet	YBOLZUJJG	O=P(O)(O)OC(C)CN
C8H19NO2			
C8H5O2P			
C9H11NO2	Phenylalan	COLNVLDH	O=C(O)C(N)CC=1C=CC=CC1
C9H11NO2	Tetrahydroi	MBFUSGLX	OC=1C=C2C(=CC1O)CCNC2
C9H11NO2	Benzoic ac	BLFLLBZGZ	O=C(OCC)C1=CC=C(N)C=C1
C9H11NO2	Phenylacet	FWTXWYXP	O=C(N)C(O)(C=1C=CC=CC1)C
C9H11NO2	Beta amino	UJOYFRCO	O=C([O-])CC(C=1C=CC=CC1)[NH3+]
C9H11NO2	Aryl alkyl ke	KTXUGZHJV	O=C(C=1C=NC=CC1)CCCO
C9H11NO2	Pyridines a	MFYZACBR	O=C(O)CCCC=1C=NC=CC1
C9H11NO2	Pyridines a	LHGVLZHIY	OC1OC(C=2C=NC=CC2)CC1
C9H11NO2	Benzamide	SBNKFTQS	O=C(N)C=1C=CC=CC1OCC
C9H11NO2	5-alkyl-2-c	RAQLHHNB	O=C(O)C1=NC=C(C=C1)CCC
C4H8N6O2			
C9H11NO3	Tyrosine an	OUYCCCA	O=C(O)C(N)CC1=CC=C(O)C=C1
C9H11NO3	Phenylalan	WRFPVMF	O=C(O)C(N)CC=1C=CC=CC1O

C9H11NO3	Beta amino	JYPHNHPX	O=C(O)CC(N)C1=CC=C(O)C=C1
C9H11NO3	Phenylalan	JZKXXXDKR	O=C(O)C(N)CC=1C=CC=C(O)C1
C9H11NO3	Phenylalan	VHVGNTVU	O=C(O)C(N)C(O)C=1C=CC=CC1
C9H11NO3	Tetrahydroi	RNIMUEXS	OC=1C=C2C(=CC1O)C(O)CNC2
C9H11NO3	Benzene an	OCSOHUR	O=C(OCC(O)C=1C=CC=CC1)N
C9H11NO3	Phenylalan	VTPJSQTVP	O=C(O)C(NO)CC=1C=CC=CC1
C9H11NO3	Alkyl-pheny	PZMVOUYY	O=C(C1=CC=C(O)C(O)=C1)CNC
C9H11NO3	Pyridines a	STZOZPPV	O=C(O)CCC(O)C=1C=NC=CC1
C6H15O4P	Dialkyl pho	WZPMZMC	O=P(O)(OC(C)C)OC(C)C
C6H15O4P	Trialkyl pho	DQWPFSLD	O=P(OCC)(OCC)OCC
C9H21NO3			
C8H18O5	Polyethylen	UWHCKJM	OCCOCCOCCOCCO
C8H18O5	Fatty alcoh	VVDQVGVV	OCC(O)C(O)C(O)C(O)C(C)C
C9H22S2			
C11H23NO	Medium-ch	GUOSQNA	O=C(O)CCCCCCCCCCN
C9H17NO4	Acyl carniti	RDHQFKQI	O=C(OC(CC(=O)[O-])C[N+](C)(C)C)C
C9H17NO4	C-glycosyl	OCLDGUC	O=C(OC)CC1OC(CO)C(N)CC1
C9H17NO4	Leucine an	BUMIGZVU	O=C(O)C(NC(=O)C(O)C)CC(C)C
C10H20O4	Fatty alcoh	GGDOZDC	OCC(O)C(=C)CCC(O)C(O)(C)C
C10H20O4	Menthane	QEFNQQR	OCC(O)(C)C1CCC(O)(C)C(O)C1
C10H20O4	Menthane	NKYSPMJO	OCC(O)(C)C1CC(O)C(C)C(O)C1
C10H20O4	Acyclic mo	WLZNCAX	OCC(=CCCC(O)(C)C(O)CO)C
C10H20O4	Fatty alcoh	QJGNMNVV	OCC(O)C(=CCC(O)C(O)(C)C)C
C10H20O4	Medium-ch	WXCLDXYQ	O=C(O)CC(O)CCCCCCCO
C10H20O4	Medium-ch	NTFDPMTZ	O=C(O)CC(O)CCCCCC(O)C
C10H20O4	Medium-ch	WZGDVNU	O=C(O)CCCCC(OC)(OC)CC
C11H16N4			
C11H11O2P			
C11H11O2P			
C3H9N6O3P			
C8H5NO6			
C9H9NOS2			
C9HN5O2			
C9H17NO5	Secondary	GHOKWGT	O=C(O)CCNC(=O)C(O)C(C)(C)CO
C10H21NS2			
C12H16N2	Hydropyrim	NVEPPWD	N1=C(C=CC=2SC=CC2C)N(C)CCC1
C12H16N2	m-Xylenes	BPICBUSO	N1=C(SCCC1)NC=2C(=CC=CC2C)C
C4H12N8O3			
C4H12N7O2P			
C10H23NO4			
C11H19N5			
C10H13N3OS			
C11H14NO2P			
C5H7N9O2			

C3H3N2O6PS
C4H4O7P2
C6H2N4S3
C14H31NO 1,2-aminoa WMUMHAZ OC(CCCCCCCCCCC)C(N)C
C14H31NO Long-chain SYELZBGX [O-][N+](C)(C)CCCCCCCCCCCC
C6H9NO5P2
C7H11NO2S3
C7H3N5O3S
C12H16NO2P
C10H22O6 Polyethylen JLFNLZLIN OCCOCCOCCOCCOCCO
C11H18N4 Dialkylaryla YFGYUFNIOO=C(OC=1N=C(N=C(C1C)C)N(C)C)N(C)C
C11H26OS2
C4H14N7O3P
C6H17N5OS2
C6H9N9O2
C14H31NO2
C12H16NO3P
C7H11N9S
C11H21N5O2
C11H29NOS2
C8H20NO6 Glycerophо SUHOQUV O=P([O-])(OCC[N+](C)(C)C)OCC(O)CO
C11H11N7O
C11H19N3S2
C9H16N5O2P
C15H31NO2
C16H9N3O
C7H19NO5P2
C8H13N5O3S
C8H21NO2S3
C7H11N5O6
C7H19NO5S2
C8H15N5OS2
C9H16N3O2PS
C12H17NS3
C12H9N5OS
C4H13N7O3S2
C7H13NO10
C16H35NO2
C16H35NO2
C15H20O5 Abscisic ac ZGHRCSAI O=C(O)C=C(C=CC1(O)C(=CC(=O)CC1(C)C)CO)C
C15H20O5 Abscisic ac AVFORCKF O=C(O)C=C(C=CC1(O)C(=CC(=O)CC1(C)CO)C)C
C15H20O5 Germacran JXXWNBNYO=C1OC2C(O)C3(C)CC(=CCC2C1=C)C(OO)CC3
C15H20O5 Abscisic ac IZGYIFFQB O=C(O)C=C(C=CC1(O)C2(OCC1(C)CC(=O)C2)C)C
C15H20O5 Terpene lac JESMSCGU O=C(O)C1=CC2C(C(=O)OC1)C3(OC3)CCC2C(C)C

C15H20O5	Sesquiterp	GLHDTCD	O=C1C(=CC2C(C1)C(C)C(O)C3(O)OCC(=C)C23O)C
C15H20O5	Naphthofur	DOHIWML	O=CC1=CCC2C(C)(C)CCC(O)C32C(=O)OC(O)C13
C15H20O5	Terpene lac	VGLFPPRJH	O=C1OCC2=C1CC(C)C3C(O)C(C(=O)O)(C)CC3C2
C15H20O5	Tetralins	FVAUGDAG	O=C1C2=C(O)C=C(OC)C=C2C(O)C(CC(O)C)C1C
C15H20O5	Sesquiterp	QVCAIXDR	O=C1C(=CC(=O)C(O)(C)C1)C2(C)C(O)CC(=O)C2(C)C
C18H35NO	Fatty amide	FATBGEAM	O=C(N)CCCCCCCC=CCCCCCCCC
C18H35NO	Morpholine	JMXKCYUT	O1C(C)CN(CC1C)C2CCCCCCCCCCC2
C13H18N2	Thiophene	OVEHKGRO	O=C(NC(=S)N1CCCCC1CC)C=2SC=CC2
C10H19O7P			
C11H23O2PS2			
C13H10N6O2			
C12H26O7	Polyethylen	IIRDTKBZIN	OCCOCCOCCOCCOCCOCCO
C13H22N4O3			
C13H30O2S2			
C7H23N8O2P			
C18H37NO	Carboximid	LYRFLYHA	O=C(N)CCCCCCCCCCCCCCCCC
C18H37NO	Alpha-amin	HNOAUFB	O=C(CCCCCCCCCCCCCCC)C(N)C
C18H37NO	Morpholine	ZFZYQMQY	O1CC(N(CCCCCCCCCCCC)CC1C)C
C18H37NO	Morpholine	SBUKOHLF	O1C(C)CN(CCCCCCCCCCCC)CC1C
C16H33NO	Alkaloids a	SEUBYHFH	OCC1NC(CCCCCCCC(O)CC)CCC1O
C16H33NO	N-acyl ami	AOMUHOF	O=C(N(CCO)CCO)CCCCCCCCCCC
C17H37NO	1,2-aminoa	KFQUQCFJ	OCC(N)C(O)CCCCCCCCCCCCCC
C13H23NO	Acyl carniti	BSVHAXJKB	O=C([O-])CC(OC(=O)CCCCC(=O)O)C[N+](C)(C)C
C13H23NO	Acyl carniti	HFCPFJNS	O=C([O-])CC(OC(=O)CC(C)CC(=O)O)C[N+](C)(C)C
C14H19N5	Pyrazolopy	OPQRBXUB	O=C(OCC)C1=CN=C2C(C=NN2CC)=C1NN=C(C)C
C14H27NOS2			
C6H23N7O4S			
C16H35NO	1,3-aminoa	OCHZTELG	OCC(N)C(O)C(O)CCCCCCCCCCC
C13H14N2	O-quinonim	XGXZRIYLD	O=C(N)C=1C(=N)C(=O)C2=C(C1O)C3OC(C)C2(O)C(O)C3
C11H19O7P			
C14H18N2OS2			
C6H14N8O4S			
C13H25N5O3			
C13H33NO2S2			
C7H26N9O2P			
C20H39NO	N-acylpyrro	ULDNZNZT	O=C(N1CCCC1)CCCCCCCCCCCCCCC
C20H41NO	Morpholine	RHIAIJKBSA	O1C(C)CN(CCCCCCCCCCCCCC)CC1C
C13H20N2	Thienamyc	ARJTYOGG	O=C(O)C1N2C(=O)C(C(O)C)C2CC1SCCNC(=O)C
C17H20N2	N-phenylth	RZHPANYK	S=C(NC=1C=CC=CC1)NCCSCC=2C=CC=CC2C
C14H21O6P			
C7H21N6O4PS			
C18H39NO	1,3-aminoa	AERBNCYC	OCC(N)C(O)C(O)CCCCCCCCCCCCC
C18H39NO	1,3-aminoa	AERBNCYC	OCC(N)C(O)C(O)CCCCCCCCCCCCC
C17H22N2O2S			

C9H18N8O5
C9H26N4O4S2
C9H34O3S4
C13H18N6 Purine nucl UQGKLARJ O=C(NCCC)C1OC(N2C=NC=3C(=NC=NC32)N)C(O)C1O
C11H23N4O5P
C13H26N2O3S2
C21H22OS
C17H29N3OS
C18H30NO2P
C9H25N9O4
C9H33N5O3S2
C11H20N2O7S
C13H17N4O4P
C15H20N2O2S2
C8H12N12OS
C15H14N6 Imidazo[1, CFSOJZTUT [N-]=[N+]=NC1=CC=C2C(=C1)C(=O)N(C)CC3=C(N=CN23)C(=O)OCC
C12H23O8P
C13H19N4O4P
C13H27O3PS2
C14H30O8 Polyethylen XPJRQAIZZ OCCOCCOCCOCCOCCOCCOCCO
C15H26N4O4
C9H27N8O3P
C9H35N4O2PS2
C10H24N9O2P
C15H27NO7
C16H23N5O3
C8H27N7O5S
C18H39NO4
C19H35N5
C19H37N5
C22H41NO N-acyl ami QQCGKIZH O=C(C=CC=CCCCCCCCCCCCCC)NCC(C)C
C10H27N9O4
C10H35N5O3S2
C18H31N3OS
C19H32NO2P
C22H43NO Fatty amide COUPDYRA O=C(N)CCCCC=CCCCCCCCCCCCCCCC
C11H33N9OS
C11H41N5S3
C16H38NO4P
C18H33N3O3
C14H18N2 N-acyl-alph OMRLTNCL O=C(NC1C(OC2=CC=C(C=C2)[N+](=O)[O-])OC(CO)C(O)C1O)C
C14H18N2 N-acyl-alph PXMQUEGJ O=C(NC1C(OC=2C=CC=CC2[N+](=O)[O-])OC(CO)C(O)C1O)C
C12H23O9P
C15H14N6O4

C15H22N2O3S2			
C15H29N5O4			
C15H37NO3S2			
C8H29N11O2S			
C9H30N9O3P			
C16H36N4O2S			
C24H32N2			
C9H28N14O			
C9H36N10S2			
C11H29N9O4			
C11H37N5O3S2			
C19H33N3OS			
C20H34NO2P			
C16H22N2	Phenoxy co	OBDCJJZZJ	O=C(OC1=CC=C(C=C1)C)NC(=O)CCSC(=S)N(CC)CC
C16H22N2	Alpha amin	WXIJHVRXT	O=C1N(C)C(SC)(C(=O)N(C)C1(SC)CO)CC=2C=CC=CC2
C15H18N2	Alpha amin	APUVOPBX	O=C(O)N(C=1C=CC=CC1N(C(=O)O)CCOC(=O)C)CC(=O)OC
C8H20N8O4P2			
C9H22N8OS3			
C20H20O6	8-prenylate	VBOYLFNG	O=C1C2=C(O)C=C(O)C(=C2OC(C3=CC=C(O)C=C3O)C1)CC=C(C)C
C20H20O6	8-prenylate	MERHMOC	O=C1C2=C(O)C=C(O)C(=C2OCC1C3=CC=C(O)C=C3O)CC=C(C)C
C20H20O6	Furanoid lig	VBIRCRCP	OC1=CC=C(C=C1OC)C2OCC3C(OCC23)C4=CC=C5OCOC5=C4
C20H20O6	3'-prenylat	SFQIGPZCF	O=C1C=2C(O)=CC(O)=CC2OC(C3=CC(O)=C(O)C(=C3)CC=C(C)C)C
C20H20O6	8-prenylate	VPCRIHAW	O=C1C2=CC=C(O)C(=C2OCC1C3=CC=C(O)C=C3O)CC=C(C)CO
C20H20O6	Flavanones	UIFXCAYU	O=C1C=2C(O)=CC(O)=CC2OC(C3=CC=C(OCC=C(C)C)C(O)=C3)C1
C20H20O6	Dibenzylbu	DIYWRNLY	OC1OCC(CC2=CC=C3OCOC3=C2)C1CC4=CC=C5OCOC5=C4
C20H20O6	Naphthale	VSXVGIMV	O=C1C=CC=2C=CC=C(OC)C2C1(OC)C3=CC(OC)=C(O)C(OC)=C3
C20H20O6	Dibenzylbu	OCTZTNYF	O=C1OCC(CC2=CC=C3OCOC3=C2)C1CC4=CC=C(O)C(OC)=C4
C20H20O6	3'-prenylat	WWUVLNP	O=C1C=2C(O)=CC(O)=C(O)C2OC(C3=CC=C(O)C(=C3)CC=C(C)C)C
C41H64O1	O-glycosyl	RPTQDUW	OC=1C=C2C=C(O)C1C(CCCC)CCCCC(C)C(OC3OCC(O)C(O)C3O)C
C21H65N16O5PS2			
C38H64N6O3S2			
C42H60N4O6			
C20H43NO	1,3-aminoa	ILMFJEORW	OCC(N)C(O)C(O)C(O)CCCCCCCCCCCCCC
C16H39N7O2			
C6H8N2O8P2S2			
C7H10N2O5S5			
C7H9O9P3S			
C8H11O6PS4			
C24H47NO			
C25H71N16O5PS			
C25H79N12O4PS3			
C30H74N8O10S			
C32H79N6O6PS2			
C21H39NO	Acyl carniti	NNCBVXBB	O=C([O-])CC(OC(=O)CCCC=CCCCCCCCC)C[N+](C)(C)C

C21H39NOFatty acid e VDPVQRIJJ O=C(OC(CCC(=O)[O-])[N+](C)(C)C)C=CCCCCCCCCCC
C21H39NOAcyl carniti BUBHHSH O=C(OC(CC(=O)[O-])C[N+](C)(C)C)C=CCCCCCCCCCC
C21H39NOAcyl carniti ABVVZYXTZ O=C([O-])CC(OC(=O)CCCCCCC=CCCCC)C[N+](C)(C)C
C21H39NON-acyl ami ASMQMRM O=C(OCC(NC(=O)CCCCCCCCCCCC)CC(=O)C)C
C10H35N13S
C14H39N7O2S
C15H40N5O3P
C16H34O9 Polyethylen GLZWNFN OCCOCCOCCOCCOCCOCCOCCOCCO
C17H30N4 Oligopeptid RUQBGIMJ O=C(O)C1N(C(=O)C(NC(=O)C(NC(=O)C(N)C)CC(C)C)C)CCC1
C11H31N8O4P
C11H39N4O3PS2
C21H41N5O
C21H49NS2
C20H28O7 Quassinoid SYRCAVSN O=C1OC2CC3C(=CC(O)C(O)C3(C)C4C5(O)OCC24C(C1)C(C)C5O)C
C20H28O7 Germacran PBRMNFXOO=C1OC2CC(C=CC(=O)C(O)(C)C(OC(=O)CC(C)C)C(O)C2C1=C)C
C20H28O7 Germacran QPYBAPKWO=C1OC2CC(C=CC(=O)C(O)(C)C(O)C(OC(=O)CC(C)C)C2C1=C)C
C20H28O7 Terpene lacMSDFIROC O=C(OC1C2=C(C(=O)OC2(O)C(O)C3CCC(O)C(C)C13C)C)C(=CC)C
C20H28O7 Colensane MLIJBZORKO=CC(=O)C1(OC1)C(O)CC2(C)C(C)C(O)C(=O)C3(C(=CCCC32)CO)C
C20H28O7 Sesquiterp BKRBOORGO=C1C=C(C(C)CO)C2(C(=O)C34OC2(O)CC1(C)C4(O)CCC(C)C3O)C
C20H28O7 Iridoids andXIQPIDYOU O=C(OCC1=COC(OC(=O)CC(C)C)C2C1=CC(O)C32OC3)CC(C)C
C20H28O7 Fatty acyl g HCXGDBG O=C(O)C1OC(OCC(=CC=2C=CC=CC2)CCCCC)C(O)C(O)C1O
C20H28O7 O-glycosyl UQYNGSP O=C1C2=C(C(=C(C=C2CC1C)C)CCOC3OC(CO)C(O)C(O)C3O)C
C14H37O5PS2
C18H18N6 Phenylmor IERNXHKD O=C(NC1=NOC=C1)CN2N=NC(=C2)CN3C(=O)COCC3C=4C=CC=CC
C18H26N2 Cumenes NUEOQLXP O=C(OC1=CC=C(C=C1)C(C)C)NC(=O)CCSC(=S)N(CC)CC
C11H18N12O2S
C17H22N2O8
C22H26O6 Terpene lacMOFNDUJX O=CC(=CCCC(=CCCC1=CC(OC1=O)(OC)C2=CC(O)=CC=C2O)C)C
C22H26O6 Cyclohexen PSMAZPOS O=C1OC(C)CC(O)CC(O)C2C(=O)C=CC3OC(C=CC=CC=CC)=C1C32
C22H26O6 Naphthofur PTADPNLC O=C1OCC2=C1CC(OC(=O)C3=CC=C(O)C=C3)C4C2(CO)CCCC4(C)
C22H26O6 Naphthofur KPUHAJPJY O=C1OCC2=C1CC(OC(=O)C3=CC=C(O)C=C3)C4C2(C)CCCC4(C)C
C22H26O6 Medium-ch FXWZJHIH O=C(O)C=CC=CC=CC=1OC2CCC(=O)C3CCCCC(OC(=O)C1C23)C
C22H26O6 Annonaceo YJGCSFJMD O=CC1=C2C=C(OC=C2C(O)C3(OC(=O)C(C(=O)CCCCCCC)=C13)C)
C22H26O6 Naphthofur HVPDKSONO=C1C(OC)=C(C(=O)C2=C3OC(C)C(C3=C(O)C=C12)(C)C(O)CC=C(
C22H26O6 Salicylic ac WNSZZRDX O=C(O)C=1C(O)=CC=CC1C=CC=CC(C(=O)C(=CC2OC2C)C)C(OC)C
C22H26O6 Naphthofur BOPUOIHQO=C1C(OC)=C(C(=O)C2=C3OC(C)C(C3=C(O)C=C12)(C)CC=CC(O)(
C22H26O6 2-arylbenz BZEQILYKR OC1=CC=C(C=C1OC)C2OC3=C(OC)C=C(C=CCOCC)C=C3C2CO
C12H31N6O4PS
C12H39N2O3PS3
C18H30N2O5S
C22H22N6O
C10H33N11O3S
C11H34N9O4P
C17H33N5O5

C17H41NO4S2			
C21H31NO	Medium-ch	SPJBXWLU	O=C(O)C=CC(=CCC1OC(C)C(NC(=O)C=CC(OC(=O)C)C)CC1C)C
C14H31N7O4S			
C14H39N3O3S3			
C22H27N5O2			
C24H70N26O3			
C35H82N10O6S			
C35H90N6O5S3			
C37H79N12O3P			
C37H87N8O2PS2			
C21H69N29O2S			
C26H82N17O4PS2			
C33H82N11O6PS			
C33H90N7O5PS3			
C41H86N5O3PS2			
C23H24O6	Rotenones	GBVCHRD	O=C1C2=CC=C(O)C(=C2OC3COC4=CC(OC)=C(OC)C=C4C13)CC=C
C23H24O6	2,2-dimeth	SYOGFYMI	O=C(C1=CC=C2OC(C=CC2=C1O)(C)C)C3C4=CC(OC)=C(OC)C=C4O
C23H24O6	Guaianolid	BBTINGNP	O=C1OC2C(C1=C)C(OC(=O)CC3=CC=C(O)C=C3)CC(=C)C4CC(O)C(
C23H24O6	Xanthones	XCRBRZW	O=C1C=2C(O)=CC(O)=CC2OC34C1=CC5C(=O)C4(OC(C)(C)C3C5)C
C23H24O6	1-benzopyr	PNGWMVL	O=C(C1=CC=C2OC(C(=C)C)CC2=C1O)C3C4=CC(OC)=C(OC)C=C4O
C23H24O6	2'-Hydroxy	JNBKPNKH	O=C(C=CC1=CC(OC)=C(OC)C=C1OC)C2=CC=C3OC(C=CC3=C2O)(
C23H24O6	Pyranoxant	QFURCBFE	O=C1C=2C(O)=C3C(OC(C)(C)CC3)=CC2OC4=CC(O)=C5OC(C)(C)C
C23H24O6	Xanthones	LYMUFMGS	O=C1C2=CC=C(O)C(O)=C2OC3=CC(O)=C(C(O)=C13)C4CC(C(=C)C
C23H24O6	2-arylbenz	MOIMOJLR	O=C(OCCCC1=CC(OC)=C2OC(=CC2=C1)C=3C=CC=4OCOC4C3)C(
C23H24O6	Pyranoflav	GEIXOHJD	O=C1C2=CC(OC)=C3OC(C=CC3=C2OC(C4=CC=C(OC)C(OC)=C4)C
C24H28O5	Coumarins	HIQLOIOG	O=C1OC=2C=C(OCC3C(=CC(=O)C4C(C)(C)C(O)CCC34C)C)C=CC2
C24H28O5	Terpene lac	PTNBQZMC	O=C1OC=2C=C(O)C=CC2C(O)=C1C(C(=CCC=C(C)CC(=O)C=C(C)C)
C24H28O5	Naphthofur	RSTDLWM	O=C(OC1CC2=C(C(=O)OC2)C3(C)C(O)CCC(C)(C)C13)C=CC=4C=C
C24H28O5	Terpene lac	VFFCJVAKV	O=C1OC=2C=C(O)C=CC2C=3OC(C)(CCC=C(C)CC(=O)C=C(C)C)C(C
C24H28O5	Terpene lac	NWTCYOA	O=C(C=C(C)C)C=C(C)CCCC1(OC=2C=3C=CC(O)=CC3OC(=O)C2C1
C24H28O5	Naphthofur	FLLYIKBNX	O=C(OC1CC2=C(C(=O)OC2)C3(C)CCCC(C)(C)C13)C=CC4=CC=C(O
C24H28O5	2'-Hydroxy-	VDTQLOAS	O=C(C1=CC=C(OCC=C(C)CC=C(C)C)C=C1O)C(O)CC2=CC=C(O)C=
C24H28O5	2'-prenylat	XCUBCLPU	O=C1C2=CC=C(O)C=C2OC(C3=CC=C(O)C(O)=C3CC=C(C)CCC(C)C
C24H28O5	8-prenylate	UUTNYYVTI	O=C1C2=C(O)C=C(O)C(=C2OC(C3=CC=C(O)C=C3)C1)CC=C(C)CC
C24H28O5	2,2-dimeth	RLFQQEPS	OC=1C=C(C=C(OC)C1OC2=CC(OC)=CC=3C=CC(OC23)(C)C)CC=C(
C20H34N2	N-acyl-alph	ZPLVYYNM	O=C(NC(C(=O)NC(C(=O)C1(OC1)CO)CC(=C)C)CO)CCCCC(C)C
C18H39O7	Trialkyl pho	WTLBZVNB	O=P(OCCOCCCC)(OCCOCCCC)OCCOCCCC
C13H34N8O4S			
C19H43O2PS2			
C21H30N6O2			
C22H29NO	Aryl ketone	KQURUXJO	O=C(C=CC=CC=CC(CO)CC)C=1C(=O)NC=C(C1O)C2(O)CCC(O)CC2
C22H29NO	Depsipepti	KFJCKGHU	O=C(C=CC(=CCO)C)NC(C(=O)OC(C)C(C(=O)O)C)C(C=1C=CC=CC1
C22H29NO	Sesquiterp	ASBKGJSD	O=C1NC(O)(CCO)C2OC12C(=O)C(=CC=CC(=CC(=CC(=CC)CO)C)C)
C22H29NO	Estrogens a	JEGKAYVSL	O=C(O)CNC(=O)COC1C2=CC(O)=CC=C2C3CCC4(C)C(O)CCC4C3C

C22H29NO	Benzoic ac	SORJFXHTZ	O=C(OC1CC2N(C)C(CC2)C1C(=O)OCCCCCC(=O)O)C=3C=CC=CC3
C22H29NO	Aminoglyco	WXCVDEUJ	O=C1OC(C(OC)=C1C)=C2OC34OC5CC(N6CCC3C65CC(O)CC)C4C
C23H25N5	Hippuric ac	DEFAKPKF	O=C(NC(C(=O)NC=1C=CC=2C=CC=CC2C1)CCCNC(=N)N)C=3C=CC
C23H25N5	Aromatic a	ZXVOFYQL	O=C(NC1=CC=C(C=C1)N(C)C)C=2N=C(C=CC2)C(=O)NC3=CC=C(C
C14H35N3O6P2			
C15H29N7O4S			
C29H80N17O5PS			
C29H88N13O4PS3			
C34H83N9O10S			
C36H88N7O6PS2			
C44H84N5O4PS			
C24H30O6	Spironolac	JUKPWJGB	O=C1OC2(CC1)CCC3C4C(C(=O)OC)CC5=CC(=O)CCC5(C)C64OC6
C24H30O6	Eremophila	UAMUYXW	O=C(OC1C=CC2=CC(=O)C(O)(C(=C)C(=O)O)CC2(C)C1C)C=CC=CC
C24H30O6	Melleolides	ISKWRTCZ	O=CC1=CC2CC(C)(C)CC2C3(C)CC(OC(=O)C=4C(O)=CC(OC)=CC4C
C24H30O6	Melleolides	GYCBSZGI	O=CC1=CC2CC(C)(C)CC2C3(C)CC(OC(=O)C=4C(O)=CC(O)=CC4C)
C24H30O6	Melleolides	WOXXSXDX	O=CC1=C2C(OC(=O)C3=C(O)C=C(OC)C=C3C)CC2(C)C4CC(C)(C)C
C24H30O6	Melleolides	BGKXQRPQ	O=CC1=CC2(O)CC(C)(C)CC2C3(C)CC(OC(=O)C=4C(O)=CC(OC)=C
C24H30O6	Dihydropyr	RVCGYSNU	O=C1OC(C)(C)C2(C=C1)C(=C3CC4(C(=C)C(C(=O)OC)(C(=O)C4O)C
C24H30O6	Sesquiterp	NZNMCSYR	O=C(C)C1CC(=O)C2(C)C34OC3(C(=O)CC12C)C5(C)CCC(=O)C(C)(C
C24H30O6	Steroid est	LMXGPAGG	O=C(OC1=CC=C2C(=C1)C(OC(=O)C)CC3C2CCC4(C)C(OC(=O)C)CC
C24H30O6	Dibenzofur	ZSIUEMMP	O=C(OC(C=1C=CC=2OC=3C(=CC(=CC3C(OC)OC)C)C2C1OC)CC(C
C21H77N21O5P2S2			
C27H92N9O4PS6			
C34H76N11O8PS			
C34H84N7O7PS3			
C48H71N5O7			
C10H17N5OS6			
C15H14NO5PS3			
C7H18N3O7PS4			
C8H22N3O2PS6			
C9H15N5O4P2S3			
C12O3S7			
C8O8S6			
C24H39NO	Aconitane-	BDCURAW	OC1C2CC3C1C(O)(CC2OC)C4CC5C6(COC)CN(CC)C4C35C(OC)CC
C11H40N11O2PS			
C17H47N3O2S3			
C18H40N5O4P			
C25H35N5O			
C13H42N7O2PS2			
C14H33N9O6			
C15H29N13O2			
C20H42NO4PS			
C22H37N3O3S			
C16H45N11S			

C21H50N3O3P
C23H45N5O2
C23H53NOS2
C30H97N11O3S6
C31H82N17O6PS
C31H90N13O5PS3
C36H85N9O11S
C39H86N11O3PS2
C24H33NO Aryl ketone SPQDIDVJA O=C(OC)NC=CCCC(C=1OC(=O)C(C(=O)C(=CC=C(C)CCCC)C)=C(O)
C24H33NO Isoindolon XWOBYGV O=C1NC(OC)C2=C3OC4(CC3=C(O)C=C12)C(C)CCC5C(C)(C)C(O)C
C10H33N13O2S2
C17H33N7O4S
C25H29N5O2
C9H29N13O7
C19H37N5 Aminocycli HEQBQGY O=CNCC(=O)N(C)C1C(O)C(OC2OC(CNCC)CCC2N)C(N)CC1OC
C13H38N9O5P
C15H33N11O4
C15H41N7O3S2
C23H37N5OS
C21H28N2 Acylamino RQVHZRRX O=CNC1=CC=CC(C(=O)NC2C(=O)OC(C)C(O)C(C(=O)OC2C)CCCC)=
C12H33N6O7PS
C12H41N2O6PS3
C14H30N8O4P2
C22H24N6O4
C22H23NO Phenanthre UYHQCZYR O=C1C=2C(O)=C3C(O)=C(OC)C(O)=CC3=C(OC)C2C4(O)C(=O)CC(O
C16H24N5 5'-acylphos CTEJAJOBM O=C(OP(=O)(O)OCC1OC(N2C=NC=3C(=NC=NC32)N)C(O)C1O)CCC
C19H23N7 Benzothiaz YRZLMZPV N=1C(=NC(=NC1N2CCOCC2)N3CCOCC3)NC=4C=CC=5N=C(SC5C4
C14H27N3O11S
C23H19N5O5
C24H30O6 Spironolac JUKPWJGB O=C1OC2(CC1)CCC3C4C(C(=O)OC)CC5=CC(=O)CCC5(C)C64OC6
C24H30O6 Melleolides ISKWRTCZ O=CC1=CC2CC(C)(C)CC2C3(C)CC(OC(=O)C=4C(O)=CC(OC)=CC4C
C24H30O6 Melleolides BGKXQRPQ O=CC1=CC2(O)CC(C)(C)CC2C3(C)CC(OC(=O)C=4C(O)=CC(OC)=C
C24H30O6 Melleolides GYCBSZGI O=CC1=CC2CC(C)(C)CC2C3(C)CC(OC(=O)C=4C(O)=CC(O)=CC4C)
C24H30O6 Melleolides WOXXSXDX O=CC1=C2C(OC(=O)C3=C(O)C=C(OC)C=C3C)CC2(C)C4CC(C)(C)C
C24H30O6 Dihydropyr RVCGYSNU O=C1OC(C)(C)C2(C=C1)C(=C3CC4(C(=C)C(C(=O)OC)(C(=O)C4O)C
C24H30O6 Eremophila UAMUYXW O=C(OC1C=CC2=CC(=O)C(O)(C(=C)C(=O)O)CC2(C)C1C)C=CC=CC
C24H30O6 Steroid est LMXGPAGG O=C(OC1=CC=C2C(=C1)C(OC(=O)C)CC3C2CCC4(C)C(OC(=O)C)CC
C24H30O6 Linear pyra AMSRLBLJ O=C1OC2=C(C=C1C(C=C)(C)C)C(O)=C3C(OC(C)(C)C(O)C3O)=C2C
C24H30O6 Sesquiterp NZNMCSYR O=C(C)C1CC(=O)C2(C)C34OC3(C(=O)CC12C)C5(C)CCC(=O)C(C)(C
C21H81N34O2PS
C26H84N26O7S
C33H94N16O6P2S
C34H88N20O4S2
C39H101N8O6PS3

C21H81N34O2PS
C26H84N26O7S
C33H94N16O6P2S
C34H104N12O2S6
C34H88N20O4S2
C26H84N26O7S
C33H94N16O6P2S
C34H104N12O2S6
C34H88N20O4S2
C39H101N8O6PS3
C29H46O4 Vitamin D a LFYYNWIV OC1C(=C)C(=CC=C2CCCC3(C)C2CCC3C4(COCC(O)(CC)CC)CC4)C
C29H46O4 Stigmastan BIDNVTVZC O=C1C2=C3C(O)CC(C(C=CC(CC)C(C)C)C)C3(C)CCC2(O)C4(C)CCC
C29H46O4 Vitamin D a RVRKNKJC OC1C(=C)C(=CC=C2CC(CC3(C)C2CCC3C(C)CCCC(O)(C)C)C4OC4)
C29H46O4 Vitamin D a PXVFRWYX O=C(OCC12CCCC(=CC=C3C(=C)C(O)CC(O)C3)C2CCC1C(C)CCCC(
C29H46O4 Dihydroxy b XCLVYKBM O=C1C=C2C3CCC(C(C)CCC(=C)C(C)COC)C3(C)CCC2C4(C)CCC(O
C29H46O4 Dihydroxy b AYCQAFST O=C(O)C(CCC(=C)C(C)C)C1CCC2C3=CCC4C(C)C(O)C(O)CC4(C)C3
C29H46O4 Triterpenoi WKRCCXB O=C(CC)C1OC2(CCC3(C4=C(CCC32C)C5(C)CCC(O)C(C)(CO)C5CC
C29H46O4 Gorgostane JYZSWLOO O=C1C=CCC2(O)C(O)CC3C4CCC(C(C)C5CC5C(C)C(C)C)C4(C)CC(
C29H46O4 Ergosterols MRERMGP O=C1C=C2C3CCC(C(C=C(C)C(C)C(C)C)C)C3(C)CCC2(O)C4(C)CCC
C29H46O4 Vitamin D a LFWSFOXG O=C(OCC12CCCC(=CC=C3C(=C)CCC(O)C3)C2CCC1C(C)CCCC(O)(
C26H37NO Sesquiterp ITWQKCOX O=C1NC(C(=O)C1=C(O)C2C(C=CC3CC(C)CC(C)C32)C(=CC)C)CC(O
C26H37NO Secondary CHSNOFZX O=C(OC1C=CC(=CCC(O)C=CC(=CC(NC(=O)C(O)C)C(C(=O)C(=CC1
C26H37NO Macrolides JMMMWKA O=C1OC(C(=CC(C(=O)CC(O)CC2CC(=O)NC(=O)C2)C)C)C(C)CCC=C
C26H37NO Dihydropyr CDYGVGC O=C1OC(C=CC(=CCC2OC(C)C(NC(=O)C=CC(OC(=O)C)C)CC2C)C)
C26H37NO Aryl alkyl ke KWYUDINJ O=C1NC=C(C(=C1C(=O)C2C(C=CC3CC(C)CCC32)C)C)C4(OC)CCC
C26H37NO Macrolides SBOCUPFP O=C1OC(C(=CC(C(=O)CCCC2CC(=O)NC(=O)C2)C)C)C(C)C(O)CC=C
C26H37NO Heterocycl VLOSGEBN O=C(O)C(O)(CC1C(=O)C(C(=O)N1C)=C(O)C=CC2C(C=CC3CCCCC3
C26H37NO Aromatic m TURMFSND O=C(C=CC=CC(=CC(C)CC(C)CC)C)C=1C(=O)N(O)C=C(C1O)C2(O)C
C26H37NO Long-chain BZNPBFDQ O=C(O)CC(O)C=CC=CC=CC(C)C(O)C(C)CC(C)C(O)CC(=O)C1=CC=
C27H33N5 Indoles and BHJFKUFPY O=C(NCCCN(C1=CC=CC(=C1)C)CC)CC(C(=O)N2C=3C=CC=CC3CC
C15H39N15O2
C15H47N11OS2
C20H52N3O4PS
C22H47N5O3S
C30H43N3O
C30H105N16O2PS6
C36H104N12O3S6
C36H112N8O2S8
C37H113N6O3PS7
C43H112N2O4S7
C27H39NO Gluco/min ILZSJEITWD O=C1C=CC2(C(=C1)CCC3C4CCC(O)(C(=O)COC(=O)CN(CC)CC)C4(
C27H39NO Long-chain WUYVILXS O=C(O)CC(O)C=CC=CC=CC(C)C(O)C(C)CC(C)C(O)CC(=O)C1=CC=
C12H43N9O6S2
C14H40N11O3PS

C20H39N7O4S			
C28H35N5O2			
C26H42N4	Dialkylaryla	NUQLUNTV	O=C(NC1CCCCC1)N(C)CC2OC3=CC=C(C=C3CC(=O)N(CC2C)C(C)
C26H42N4	N-arylamid	WWPXFQL	O=C1C2=CC(=CC=C2OC(CN(C)CC3CC3)C(C)CN1C(C)CO)NC(=O)C
C18H46N6O6S			
C20H43N8O3P			
C20H51N4O2PS2			
C25H46O8			
C21H41N5	Aminocycli	ZBGPYVZLY	OC1COC(OC2C(O)C(OC3OC(=CCC3N)CN)C(N)CC2NCC)C(O)(C)C1
C10H37N17O3S			
C11H38N15O4P			
C17H37N11O5			
C17H45N7O4S2			
C26H52O7	Fatty acyl g	NNSQMUP	OCC1OC(OCC(O)CCCCCCCCCCCCCCCCCC)C(O)C(O)C1O
C20H48N10OS			
C21H49N8O2P			
C27H48N4O3			
C27H56O2S2			
C23H44NO	1-acyl-sn-g	DBHKHNG	O=C(OCC(O)COP(=O)(O)OCCN)CCCCCCCC=CCC=CCCCCC
C23H44NO	2-acyl-sn-g	SVRBKLJID	O=C(OC(CO)COP(=O)(O)OCCN)CCCCCCCC=CCC=CCCCCC
C25H39N3	Peptides	USCBZDVD	O=C(OC)C(NC(=O)C(N(C(=O)C(NC(=O)C)C(C)CC)C)CC1=CC=C(O)C
C26H35N7	N-benzylpi	CICDSYWW	N#CN=C(NCCNC(=O)C1=CC=C(N)C=C1)NCCCOC2=CC=CC(=C2)C
C18H39N9O4S			
C26H43N3OS2			
C23H48NO	1-acyl-sn-g	BBYWOYAF	O=C(OCC(O)COP(=O)(O)OCCN)CCCCCCCCCCCCCCCCC
C23H48NO	1-acyl-sn-g	RJZVWDTY	O=C(OCC(O)COP(=O)([O-])OCC[N+](C)(C)C)CCCCCCCCCCCCCC
C23H48NO	2-acyl-sn-g	KIHAGWUU	O=C(OC(CO)COP(=O)(O)OCCN)CCCCCCCCCCCCCCCCC
C23H48NO	1-alkyl,2-a	LEBRETLBL	O=C(OC(COCCCCCCCCCCCCCCCC)COP(=O)([O-])OCC[NH3+])C
C11H43N15O2S2			
C16H48N7O5PS			
C25H43N3O6			
C30H43NO4			
C36H108N12O4S6			
C37H101N14O6PS3			
C37H109N10O5PS5			
C44H109N4O7PS4			
C45H113N4O2PS6			
C29H40O6	Oxasteroid	JQSXTZPTX	O=C(OCC1(O)CCC23CC1CC3CCC4C5(C)COC(OC5CCC42C)C6=CC
C29H40O6	Pyrans	OSMGLBXN	O=C(OC)C(C)C1OC(=O)C(C)C1(C=CC=CC=CC=CC=C(C)C2OC(C)C
C29H40O6	Triterpenoi	AXIFNPRFD	O=C(OCC)CCC(C)C1CC(=O)C2(C=3C(=O)CC4C(C(=O)CCC4(C3C(=
C29H40O6	Chromone	BXPDHYNA	O=C1C=2C(OC(=C1C)CCC(C)C(OC)C(C)C(OC)C=CC=CC(=CC)C)=C
C30H36N4	Biphenyls a	BRTODLBR	O=C(C1=CC=C(C=C1)C=2C=CC(=CC2)C3C(N4CCCCN(CC=5C=NC=
C30H36N4	Biphenyls a	KXRXTXUV	O=C(C1=CC=CC(=C1)C2=CC=C(C=C2)C3C(N4CCCCN(CC=5C=NC=
C30H36N4	Biphenyls a	TZPADAHR	O=C(C1=CC=C(C=C1)C=2C=CC(=CC2)C3C(N4CCCCN(CC=5C=CN=

C30H36N4	Alpha carb	RMGREFLD	O=C(N1CCC23C=4C=CC=CC4NC5N(C6=CC=CC7=C6C53CCN(C7C
C14H46N8O4P2S			
C15H40N12O2S2			
C12H25N4O7PS4			
C13H21N8O3PS4			
C13H29N4O2PS6			
C14H22N6O4P2S3			
C18H16N4O9S2			
C32H39NO	Diphenylm	RWTNPBW	O=C(O)C(C1=CC=C(C=C1)C(O)CCCN2CCC(CC2)C(O)(C=3C=CC=C
C25H44NO	1-acyl-sn-g	ROPRRXYV	O=C(OCC(O)COP(=O)(O)OCCN)CCCC=CCC=CCC=CCC=CCCCCC
C25H44NO	2-acyl-sn-g	YWOCITMX	O=C(OC(CO)COP(=O)(O)OCCN)CCCC=CCC=CCC=CCC=CCCCCC
C32H39NO	Naphthopy	YVDJBQQJI	O=C1C=C2C3(OC1C(O3)(C)C)CCC4(C)C2(O)CCC5CC=6C=7C(=CC
C25H44NO	1-acyl-sn-g	JPNPIRVRG	O=C(OCC(O)COP(=O)(O)OCCN)CCCCCCC=CCC=CCC=CCC=CCC
C25H44NO	2-acyl-sn-g	BAKUYTLD	O=C(OC(CO)COP(=O)(O)OCCN)CCCCCCC=CCC=CCC=CCC=CCC
C32H39NO	Naphthopy	FNTKJPWO	O=C1C=C2C(OC1C(O)(C)C)=CCC3(C)C2(O)CCC4CC=5C=6C=C(C=
C32H39NO	Alkyl aryl et	SELHCSAK	O=C1NC2(O)CC3=CC=C(OC4C5C6C(=O)C1(C=CC6(C=C(C)C5C7(C
C32H39NO	Naphthopy	WCISYVSW	O=C1C=C2C3(OC1C(O3)(C)C)CCC4(C)C2(O)CCC5CC=6C=7C=C(C
C24H43N3O6S			
C29H43NO	Pyrrolidine-	AEZPXPDC	O=C1NC(O)C(=O)C1=C(O)C2(C)C(C=CC3CC(C)CC(C)C32)CC(O)C=
C14H47N9O6S2			
C16H44N11O3PS			
C21H47N3O8S			
C30H39N5O2			
C19H47N7O6S			
C19H55N3O5S3			
C20H48N5O7P			
C21H44N9O3P			
C21H52N5O2PS2			
C33H115N19OS7			
C40H115N13O3S6			
C40H123N9O2S8			
C45H120N5O6PS5			
C46H116N9O2PS5			
C40H107N17O4S4			
C40H115N13O3S6			
C40H123N9O2S8			
C45H112N9O7PS3			
C45H120N5O6PS5			
C33H107N23O2S5			
C38H112N15O5PS4			
C40H115N13O3S6			
C45H120N5O6PS5			
C46H116N9O2PS5			
C17H50N10O4S			

C23H51N6O3P
C23H59N2O2PS2
C24H50N4O6
C25H46N8O2
C28H47NO Macrolides HSRFFKHG O=C(O)C1CCCC1C2OC(=O)CC(O)C(C)CC(C)CC(C)CC(C)C(O)C(=C
C14H39N17O3
C14H47N13O2S2
C14H55N9OS4
C29H43N5O2
C33H109N23O3S5
C40H117N13O4S6
C45H114N9O8PS3
C46H126N5O2PS7
C47H119N7O4P2S4
C12H41N17O4S
C17H46N9O7P
C19H41N11O6
C19H49N7O5S2
C20H37N15O2
C42H123N9O3S8
C42H131N5O2S10
C43H124N7O4PS7
C49H123N3O5S7
C67H99N3O7
C33H38O6 Triacylglyce ZSDBFLMJV O=C(OCC(OC(=O)CCCC=1C=CC=CC1)COC(=O)CCCC=2C=CC=CC2
C10H38N14O9S
C17H48N4O8P2S
C18H42N8O6S2
C20H39N10O3PS
C16H51N11O2S2
C17H52N9O3PS
C23H51N5O4S
C23H59NO3S3
C31H47N3O2
C52H99N1 Oligopeptid AYZHFLVN O=C(NC(C(=O)NC1C(=O)NC(C(=O)NC(C(=O)NC(C(=O)NC(C(=O)NC
C40H116N15O7PS4
C46H123N7O7S6
C48H120N9O4PS5
C48H128N5O3PS7
C12H12O12S6
C6H15N4O9P3S5
C8H18N2O7P2S7
C9H12N6O5S8
C17H46N11O3PS2

C17H54N7O2PS4
C24H46N5O5PS
C33H41NO6
C14H45N17O5S
C14H53N13O4S3
C21H55N7O4P2S
C22H41N15O3
C40H127N18O3PS6
C46H134N10O3S8
C47H127N12O5PS5
C52H132N4O8P2S4
C30H55N5 Cyclic pept BXWBHHN O=C1NC(C(=O)NC(C(=O)N(C)C(C(=O)NC(C(=O)NC1CC(C)C)CC(C)C
C30H55N5 OligopeptidMEJHOHN O=C1NC(C(=O)NC(C(=O)NC(C(=O)NC(C(=O)NC1CC(C)C)CC(C)C)C
C30H55N5 Cyclic pept HRBCXMVT O=C1NC(C(=O)NC(C(=O)NC(C(=O)NC(C(=O)NC1CC(C)C)CC(C)C)C
C16H47N21O2
C21H60N9O4PS
C28H60N3O6P
C30H55N5 Cyclic pept BXWBHHN O=C1NC(C(=O)NC(C(=O)N(C)C(C(=O)NC(C(=O)NC1CC(C)C)CC(C)C
C30H55N5 Cyclic pept HRBCXMVT O=C1NC(C(=O)NC(C(=O)NC(C(=O)NC(C(=O)NC1CC(C)C)CC(C)C)C
C30H55N5 OligopeptidMEJHOHN O=C1NC(C(=O)NC(C(=O)NC(C(=O)NC(C(=O)NC1CC(C)C)CC(C)C)C
C16H47N21O2
C21H60N9O4PS
C23H55N11O3S
C44H125N16O7PS4
C51H127N10O7P3S2
C51H135N6O6P3S4
C52H129N10O4PS5
C38H77NON-acyl ami DAGLNHFU O=C(NC(C)C(O)CCCCCCCCCCCCCCC)CCCCCCCCCCCCCCCCC
C23H58N12O3S
C23H66N8O2S3
C28H63N4O6P
C34H62O7
C42H135N14O3PS8
C48H142N6O3S10
C49H135N8O5PS7
C49H143N4O4PS9
C30H55N5 Cyclic pept BXWBHHN O=C1NC(C(=O)NC(C(=O)N(C)C(C(=O)NC(C(=O)NC1CC(C)C)CC(C)C
C30H55N5 OligopeptidMEJHOHN O=C1NC(C(=O)NC(C(=O)NC(C(=O)NC(C(=O)NC1CC(C)C)CC(C)C)C
C30H55N5 Cyclic pept HRBCXMVT O=C1NC(C(=O)NC(C(=O)NC(C(=O)NC(C(=O)NC1CC(C)C)CC(C)C)C
C21H60N9O4PS
C23H55N11O3S
C28H60N3O6P
C30H27NO Xanthones CIRUKWHI O=C(O)C(NC(=O)C=1C(O)=C2C=3C(O)=C4C(=O)C5=C(O)C(OC)=CC
C10H28N17O7PS2

C15H41N5O9P2S3
C17H38N7O6P3S2
C43H124N20O7P2S3
C49H139N8O6PS7
C51H128N14O4P2S4
C56H123N10O10PS2
C45H126N16O5P2S5
C50H129N8O10PS5
C51H124N10O10P4S
C52H118N14O8P2S2
C14H35N7O9P2S3
C15H37N7O6S6
C15H39N7O4P2S5
C16H32N9O6P3S2
C23H62N11O3PS
C23H70N7O2PS3
C28H65N3O8S
C30H62N5O5P
C16H51N17O4P2
C20H61N7O7S3
C22H50N13O5P
C22H58N9O4PS2
C38H51NO19-oxoster PEJCOIYIV O=CC12C(O)CC(OC2CCC3(C)C1CCC4CC=5C=6C(=CC=C7C(=O)C8
C38H51NONaphthofur BVXANUMP O=C(O)C(C)C(O)C1OC2(O)CCC3(C)C(CCC4CC=5C6=CC7=C(C=C6
C15H53N15O7P2
C18H44N21O2P
C22H61N5O8P2S
C34H44N4 Biphenyls aNZSCNZRA O=C(NCCCN1CCOCC1)C=2N=C(C=3C=CC=C(C3)C=4C=CC=C(OC)
C13H41N20O5PS
C30H52O9S2
C37H40N4O5
C43H134N23O5PS5
C55H139N9O10P2S3
C57H144N7O6P3S4
C58H146N7O3PS7
C25H59N11O5S
C25H67N7O4S3
C27H56N13O2P
C32H59N5O7
C50H136N17O8PS4
C57H146N7O7P3S4
C58H140N11O5PS5
C58H148N7O4PS7
C18H54N8O8P2S2

C27H52N6O3S4
C30H44N4O11
C31H48N4O6S2
C18H51N7O11S3
C21H60N5O2PS6
C28H44N7O6PS
C28H52N3O5PS3
C16H52N11O11PS
C18H57N9O7P2S2
C19H43N17O6S
C26H53N7O5P2S
C13H47N7O7P2S4
C14H41N11O5S5
C14H49N7O4S7
C21H51NO4P2S5
C13H46N15O11PS
C16H37N21O6S
C23H47N11O5P2S
C38H41N3O7
C18H55N17O5P2
C19H49N21O3S
C24H62N9O5PS2
C41H53N3O4
C17H64N7O6PS5
C19H53N13O4P2S2
C27H47N11O4S2
C42H43N3O4
C39H74O6 TriacylglyceVMPHSYLJ O=C(OCC(OC(=O)CCCCCCCCCCC)COC(=O)CCCCCCCCCCC)CCC
C39H74O6 TriacylglyceZXYLKHUM O=C(OCC(OC(=O)CCCCCCCCC(C)CC)COC(=O)CCCCCCCCCC(C)C
C39H74O6 TriacylglyceJRPDSJAPMO=C(OCC(OC(=O)CCCCCCCCCC(C)C)COC(=O)CCCCCCCCC(C)CC
C39H74O6 TriacylglyceISMNGDXY O=C(OCC(OC(=O)CCCCCCCCC)COC(=O)CCCCCCCCC(C)CC)CCC
C39H74O6 TriacylglyceVHDDDZW O=C(OCC(OC(=O)CCCCCCCCCC(C)C)COC(=O)CCCCCCCCCC(C)C
C39H74O6 TriacylglyceLASNFXGL O=C(OCC(OC(=O)CCCCCCCCC(C)CC)COC(=O)CCCCCCCCC(C)CC
C39H74O6 TriacylglyceJNHKUQJE O=C(OCC(OC(=O)CCCCCCCCC(C)C)COC(=O)CCCCCCCCC(C)C)C
C39H74O6 TriacylglyceKDENWKP O=C(OCC(OC(=O)CCCCCCCCCCC)COC(=O)CCCCCCCCC(C)C)CC
C39H74O6 TriacylglyceYHFFSYAK O=C(OCC(OC(=O)CCCCCCC)COC(=O)CCCCCCCCC(C)C)CCCCCC
C39H74O6 TriacylglyceQJVZVEYEMO=C(OCC(OC(=O)CCCCCCCCC(C)C)COC(=O)CCCCCCCCC(C)C)C
C28H68N6O5P2S
C29H62N10O3S2
C42H63O4P
C44H58N2O3
C12H7NO6P2S11
C37H86N8S
C40H82N8

C44H86N2O2
C24H64N16O5
C31H72N6O6S
C31H74N6O4P2
C32H68N10O2S
C24H64N16O5
C31H72N6O6S
C31H74N6O4P2
C32H68N10O2S
C20H62N19O3PS
C25H65N11O8S
C26H69N11O3S3
C33H69N5O5S2
C17H61N25O5
C24H71N15O4P2
C25H65N19O2S
C30H78N7O4PS2
C23H70N17O3PS
C29H69N13O4S
C29H77N9O3S3
C36H77N3O5S2
C17H60N24O6
C24H70N14O5P2
C25H64N18O3S
C30H77N6O5PS2
C40H70O8 Diterpene gYHLSUHMNO=C(O)C(C)CC(C)CC(C)CC(C(=O)C=C(O)C(C)CC(C)CC=CC(C)C(O)
C23H75N12O2PS3
C29H82N4O2S5
C36H74N2O5S2
C39H74NO Phosphatid HBZNVZIRJO=C(OCC(OC(=O)CCCCCCCC=CCC=CCCCCC)COP(=O)(O)OCCN)C
C39H74NO Phosphatid UIELP0KGT O=C(OCC(OC(=O)CCCCCCCC=CCCCCCC)COP(=O)(O)OCCN)CCCC
C39H74NO Phosphatid XEPGAKWBO=C(OCC(OC(=O)CCCCCCCCCCCCCCC)COP(=O)(O)OCCN)CCCC
C39H74NO Phosphatid RXKSKZZA O=C(OCC(OC(=O)CCCCCCCCCC=CCC=CCCCCC)COP(=O)(O)OCC
C39H74NO Phosphatid QVAHLHSHO=C(OCC(OC(=O)CCCCCCCCCCCCC)COP(=O)(O)OCCN)CCCCCC
C39H74NO Phosphatid DWHRTOHO=C(OCC(OC(=O)CCCCCCCC=CCCCC)COP(=O)(O)OCCN)CCCCCC
C39H74NO Phosphatid UALNVYZBI O=C(OCC(OC(=O)CCCCCCCCCC=CCCCCCCCC)COP(=O)(O)OCCN
C39H74NO Phosphatid RAMNOXD O=C(OCC(OC(=O)CCCCCCCC=CCCCCCCCC)COP(=O)(O)OCCN)CC
C39H74NO Phosphatid VYIZOJIGQO=C(OCC(OC(=O)CCCCCCCC=CCC=CCCCCC)COP(=O)([O-])OCC[
C39H74NO Phosphatid RFJWWQWO=C(OCC(OC(=O)CCCCCCCCCCC)COP(=O)([O-])OCC[N+](C)(C)C
C41H74NO 1-(1Z-alken UUYSKERS O=C(OC(COC=CCCCCCCCCCCCCCC)COP(=O)(O)OCCN)CCCC=CC
C41H74NO 1-(1Z-alken DPYGCLW O=C(OC(COC=CCCCCCCCCCCCCCCCC)COP(=O)(O)OCCN)CCCC
C41H74NO 1-alkyl,2-a NTBDNAW O=C(OC(COCCCCCCCCCCCCCCCC)COP(=O)(O)OCCN)CCCC=CC
C41H74NO Glycerophо BVLZMCLC O=C(OCC(OC=CCCCCCCCCCCCCCC)COP(=O)(O)OCCN)CCCC=CC
C41H74NO Glycerophо JJISWJXUIHO=C(OCC(OC=CCCCCCCCCCCCCCCCC)COP(=O)(O)OCCN)CCCC

C41H74NO 1-(1Z-alken YITZQGLIIC O=C(OC(COC=CCCCCCCCCCCCCCC)COP(=O)(O)OCCN)CCCCCC
C41H74NO 1-(1Z-alken SPQOYUSS O=C(OC(COC=CCCCCCCC=CCCCCCCCC)COP(=O)(O)OCCN)CCCC
C41H74NO 1-(1Z-alken ADWDFBQ O=C(OC(COC=CCCCCCCCC=CCCCCCC)COP(=O)(O)OCCN)CCCC
C41H74NO Glycerophо SRUAKYVB O=C(OCC(OC=CCCCCCCCCCCCCCC)COP(=O)(O)OCCN)CCCCCC
C41H74NO Glycerophо XLBRCASD O=C(OCC(OC=CCCCCCCC=CCCCCCCCC)COP(=O)(O)OCCN)CCCC
C54H71N8O8PS15
C62H172N11O7PS8
C63H149N21O11P2S
C64H169N13O4P2S7
C18H47N27O6
C23H68N11O7PS3
C30H60N9O10P
C31H80NO3PS6
C29H72N12O6S
C29H80N8O5S3
C31H69N14O3P
C37H76N6O3S2
C41H74NO Phosphatid DRIVXEVM O=C(OCC(OC(=O)CCCC=CCC=CCC=CCC=CCCCCC)COP(=O)(O)OC
C41H74NO Phosphatid DPPYJZPU O=C(OCC(OC(=O)CCCCC=CCC=CCC=CCC=CCC)COP(=O)([O-])OC
C41H74NO Phosphatid GEVCEBBG O=C(OCC(OC(=O)CCCCCCCCCCCCCC)COP(=O)([O-])OCC[N+](C)(
C41H74NO Phosphatid DUQDVNA O=C(OCC(OC(=O)CCCCCCCCCCCCCCC)COP(=O)(O)OCCN)CCCC
C41H74NO Phosphatid SSCDRSKJ O=C(OCC(OC(=O)CCCCCCCC=CCC=CCCCCC)COP(=O)(O)OCCN)C
C41H74NO Phosphatid KZLUVTCX O=C(OCC(OC(=O)CCCCC=CCC=CCC=CCC=CCC)COP(=O)(O)OCCN
C41H74NO Phosphatid PAACCHRP O=C(OCC(OC(=O)CCCCCCCCCCCCCCCCC)COP(=O)(O)OCCN)CC
C41H74NO Phosphatid NUDNBLVZ O=C(OCC(OC(=O)CCCCC=CCC=CCC=CCCCCC)COP(=O)(O)OCCN)
C41H74NO Phosphatid NKVWDIUB O=C(OCC(OC(=O)CCCCCCCC=CCCCCCCCC)COP(=O)(O)OCCN)CC
C41H74NO Phosphatid KUPNWZA O=C(OCC(OC(=O)CCCCCC=CCC=CCC=CCC=CCCCCC)COP(=O)(O
C41H76NO Phosphatid LMWFNZU O=C(OCC(OC(=O)CCCCCCC=CCC=CCC=CCCCCC)COP(=O)(O)OC
C41H76NO Phosphatid GKAFCSRK O=C(OCC(OC(=O)CCCCCCCC=CCC=CCCCCC)COP(=O)(O)OCCN)C
C41H76NO Phosphatid XECLFWW O=C(OCC(OC(=O)CCCCCCCCCCCCCC)COP(=O)([O-])OCC[N+](C)(
C41H76NO Phosphatid VZPPRGZN O=C(OCC(OC(=O)CCCCC=CCC=CCC=CCCCCC)COP(=O)([O-])OCC
C41H76NO Phosphatid HVDYTORI O=C(OCC(OC(=O)CCCCCCCCCCCCCCCCC)COP(=O)(O)OCCN)CC
C41H76NO Phosphatid QTRPWCP O=C(OCC(OC(=O)CCCCC=CCC=CCC=CCCCCC)COP(=O)(O)OCCN)
C41H76NO Phosphatid DVZYZHDJ O=C(OCC(OC(=O)CCCCCCCCCCCCCC)COP(=O)([O-])OCC[N+](C)(
C41H76NO Phosphatid FHTHYAKS O=C(OCC(OC(=O)CCCCCCCC=CCC=CCC=CCC)COP(=O)([O-])OCC
C41H76NO Phosphatid DCPDPBC O=C(OCC(OC(=O)CCCCCCCC=CCC=CCC=CCC)COP(=O)(O)OCCN)
C41H76NO Phosphatid NTINXHSIN O=C(OCC(OC(=O)CCCCCCCCCCCCCCCCC)COP(=O)(O)OCCN)CC
C43H74NO 1-(1Z-alken WVGALBKS O=C(OC(COC=CCCCCCCCCCCCCCC)COP(=O)(O)OCCN)CCC=CCC
C43H74NO 1-(1Z-alken RDLRIZLZW O=C(OC(COC=CCCCCCCCC=CCCCCCC)COP(=O)(O)OCCN)CCCC
C43H74NO 1-(1Z-alken VGXSEFHX O=C(OC(COC=CCCCCCCC=CCCCCCCCC)COP(=O)(O)OCCN)CCCC
C43H74NO Glycerophо SBDGZMPE O=C(OCC(OC=CCCCCCCCC=CCCCCCC)COP(=O)(O)OCCN)CCCC
C43H74NO Glycerophо UKJIXBCSU O=C(OCC(OC=CCCCCCCC=CCCCCCCCC)COP(=O)(O)OCCN)CCCC
C43H74NO Glycerophо CGFAXGUA O=C(OCC(OC=CCCCCCCCCCCCCCC)COP(=O)(O)OCCN)CCC=CCC
C23H65N21O5S

C30H75N11O4P2S
C45H69N3O6
C19H71N23O4S2
C27H75N17OS3
C34H67N15O4
C42H71N9OS
C43H74NO Phosphatid MPWUZHV O=C(OCC(OC(=O)CCC=CCC=CCC=CCC=CCC=CCC=CCC)COP(=O)
C43H74NO Phosphatid CNFOLWF O=C(OCC(OC(=O)CCCCCCCCCCCCCCC)COP(=O)(O)OCCN)CCC=C
C43H74NO Phosphatid IFUXMIFAC O=C(OCC(OC(=O)CCCCCCCC=CCCCCCCCC)COP(=O)(O)OCCN)CC
C43H74NO Phosphatid AMKOUQL O=C(OCC(OC(=O)CCCC=CCC=CCC=CCC=CCC=CCC)COP(=O)(O)O
C43H74NO Phosphatid VRBJSZDBP O=C(OCC(OC(=O)CCCCCCCC=CCC=CCCCCC)COP(=O)(O)OCCN)C
C43H74NO Phosphatid LFGBKOUQ O=C(OCC(OC(=O)CCCC=CCC=CCC=CCC=CCCCCC)COP(=O)(O)OC
C43H74NO Phosphatid LUYFBLCII O=C(OCC(OC(=O)CCCCCCCCCC=CCC=CCCCCC)COP(=O)(O)OCC
C43H74NO Phosphatid BXGABXGD O=C(OCC(OC(=O)CCCCC=CCC=CCC=CCC=CCC)COP(=O)(O)OCCN
C43H74NO Phosphatid CZYPIOIKF O=C(OCC(OC(=O)CCCCCCCCCCCC)COP(=O)([O-])OCC[N+](C)(C)C
C43H74NO Phosphatid IGTWLVVZX O=C(OCC(OC(=O)CCC=CCC=CCC=CCC=CCC=CCC=CCC)COP(=O)
C23H67N25O3
C23H75N21O2S2
C35H80N7O7P
C38H71N13O2
C43H76NO Phosphatid PECSWFQ O=C(OCC(OC(=O)CCCC=CCC=CCC=CCC=CCCCCC)COP(=O)(O)OC
C43H76NO Phosphatid LSYAJRTVZ O=C(OCC(OC(=O)CCCCCCCCCCCCCC)COP(=O)([O-])OCC[N+](C)(
C43H76NO Phosphatid UJMUDAMK O=C(OCC(OC(=O)CCCC=CCC=CCC=CCC=CCC=CCC)COP(=O)([O-]
C43H76NO Phosphatid VFUVYNGT O=C(OCC(OC(=O)CCCCCCCC=CCCCCCCCC)COP(=O)(O)OCCN)CC
C43H76NO Phosphatid MOJMRZJN O=C(OCC(OC(=O)CCCC=CCC=CCC=CCC=CCC=CCC)COP(=O)(O)O
C43H76NO Phosphatid ZAVFAQIEE O=C(OCC(OC(=O)CCCCCCCCCCCCCCCCC)COP(=O)(O)OCCN)CC
C43H76NO Phosphatid VWMUCYID O=C(OCC(OC(=O)CCCCC=CCC=CCC=CCC=CCC)COP(=O)(O)OCCN
C43H76NO Phosphatid LQTOTSOX O=C(OCC(OC(=O)CCCCCCCCCC=CCCCCCCCC)COP(=O)(O)OCCN
C43H76NO Phosphatid KCJHLHNK O=C(OCC(OC(=O)CCCCC=CCC=CCC=CCCCCC)COP(=O)(O)OCCN)
C43H76NO Phosphatid DKGGFNCT O=C(OCC(OC(=O)CCCCCCCCCC=CCC=CCCCCC)COP(=O)(O)OCC
C43H78NO Phosphatid ANRKEHN O=C(OCC(OC(=O)CCCC=CCC=CCC=CCC=CCCCCC)COP(=O)(O)OC
C43H78NO Phosphatid ZSWHHKKY O=C(OCC(OC(=O)CCCC=CCC=CCC=CCC=CCCCCC)COP(=O)([O-])
C43H78NO Phosphatid SQGZFCFL O=C(OCC(OC(=O)CCCCCC=CCC=CCC=CCC=CCCCCC)COP(=O)(O
C43H78NO Phosphatid DCYLYWXG O=C(OCC(OC(=O)CCCCCCCCCCCCCC)COP(=O)([O-])OCC[N+](C)(
C43H78NO Phosphatid FKCXXHSN O=C(OCC(OC(=O)CCCCCCCCCCCCCCCCC)COP(=O)(O)OCCN)CC
C43H78NO Phosphatid NYRDUZVM O=C(OCC(OC(=O)CCCCC=CCC=CCC=CCC=CCC)COP(=O)(O)OCCN
C43H78NO Phosphatid HKJCCNCC O=C(OCC(OC(=O)CCCCCCCCCCCCCCCCCCC)COP(=O)(O)OCCN)
C43H78NO Phosphatid MQXKQCU O=C(OCC(OC(=O)CCCC=CCC=CCC=CCCCCCCCC)COP(=O)(O)OC
C43H78NO Phosphatid JDMYIRPBI O=C(OCC(OC(=O)CCCCCCCCCC=CCCCCCCCC)COP(=O)(O)OCCN
C43H78NO Phosphatid BAORKIQZJ O=C(OCC(OC(=O)CCCCC=CCC=CCC=CCCCCC)COP(=O)(O)OCCN)
C29H84N13O2PS2
C34H79N9O8
C42H83N3O5S
C44H80N5O2P

C60H174N24O6S8			
C60H77N15O4S16			
C67H184N14O5P2S8			
C68H178N18O3S9			
C21H69N29O2S			
C26H82N17O4PS2			
C33H82N11O6PS			
C41H86N5O3PS2			
C31H82N18O2S			
C32H83N16O3P			
C38H82N12O4			
C38H90N8O3S2			
C28H72N24O4			
C28H80N20O3S2			
C33H85N12O6PS			
C41H89N6O3PS2			
C48H80NO	1-acyl,2-al	PLOWNUX	O=C(OCC(OCCCCCCCCC1CCC2C(C1)C3C4CCC4C23)COP(=O)([O-
C19H76N25O4PS2			
C31H81N11O9P2			
C32H91N7O5S5			
C51H94O6	Triacylglyce	RUOVJPPU	O=C(OCC(OC(=O)CCCCCCCC=CCCCCCC)COC(=O)CCCCCCCCCC
C51H94O6	Triacylglyce	UKADBOO	O=C(OCC(OC(=O)CCCCCCCCCCCCC)COC(=O)CCCCCCCCCCCCC
C51H94O6	Annonaceo	GBSUIQLR	O=C1OC(C=C1CCCCCCCCCCCCC(OC(=O)CCCCCCCCCCCCCCC)
C51H94O6	Triacylglyce	YGWWTTC	O=C(OCC(OC(=O)CCCCCCCCCCCCC)COC(=O)CCCCCCCCCC=CC
C51H94O6	Triacylglyce	KIFHLTOCI	O=C(OCC(OC(=O)CCCCCCCCCCCCC)COC(=O)CCCCCCCC=CCCC
C51H94O6	Triacylglyce	YUIIXXZIUI	O=C(OCC(OC(=O)CCCCCCCCCCCCCCC)COC(=O)CCCCCCCCCCC
C51H94O6	Triacylglyce	QMWRBKD	O=C(OCC(OC(=O)CCCCCCCCCCCCCC)COC(=O)CCCCCCCCCCCC
C51H94O6	Triacylglyce	ZBGYWUIS	O=C(OCC(OC(=O)CCCCCCCCCCCCC)COC(=O)CCCCCCCCCCCCC
C51H94O6	Triacylglyce	QABWZGM	O=C(OCC(OC(=O)CCCCCCCC=CCCCCCC)COC(=O)CCCCCCCCCC
C51H94O6	Triacylglyce	IDORTGNL	O=C(OCC(OC(=O)CCCCCCCCCCCCCCC)COC(=O)CCCCCCCC=CC
C51H96O6	Triacylglyce	FEKLSEFRU	O=C(OCC(OC(=O)CCCCCCCCCCCCCCC)COC(=O)CCCCCCCCCCC
C51H96O6	Triacylglyce	GPXZOHHI	O=C(OCC(OC(=O)CCCCCCCCCCCCCCC)COC(=O)CCCCCCCCCCC
C51H96O6	Triacylglyce	OXKCYPBF	O=C(OCC(OC(=O)CCCCCCCCCCCCC)COC(=O)CCCCCCCCCCCCC
C51H96O6	Triacylglyce	GDRDGFO	O=C(OCC(OC(=O)CCCCCCCCCCCCC)COC(=O)CCCCCCCCCCCCC
C51H96O6	Triacylglyce	AXBFGYMT	O=C(OCC(OC(=O)CCCCCCCC=CCCCCCC)COC(=O)CCCCCCCCCC
C51H96O6	Triacylglyce	PSEFNWW	O=C(OCC(OC(=O)CCCCCCCCCCCCCC)COC(=O)CCCCCCCCCCCC
C51H96O6	Triacylglyce	YUNYDLOK	O=C(OCC(OC(=O)CCCCCCCC=CCCCCCCCC)COC(=O)CCCCCCCC
C51H96O6	Triacylglyce	CUFCTEMV	O=C(OCC(OC(=O)CCCCCCCCCCCCCCC)COC(=O)CCCCCCCCCCC
C51H96O6	Triacylglyce	WIPOYBVE	O=C(OCC(OC(=O)CCCCCCCC=CCCCC)COC(=O)CCCCCCCCCCCC
C51H96O6	Triacylglyce	JEVVUGAO	O=C(OCC(OC(=O)CCCCCCCCCCCCC)COC(=O)CCCCCCCCCCCCC
C51H98O6	Triacylglyce	PVNIQBQS	O=C(OCC(OC(=O)CCCCCCCCCCCCCCC)COC(=O)CCCCCCCCCCC
C51H98O6	Triacylglyce	JTDPHURH	O=C(OCC(OC(=O)CCCCCCCCCCCCCCC)COC(=O)CCCCCCCCCCC
C51H98O6	Triacylglyce	SHXPYMW	O=C(OCC(OC(=O)CCCCCCCCCCCCCCCC)COC(=O)CCCCCCCCCC
C51H98O6	Triacylglyce	KHVONBKU	O=C(OCC(OC(=O)CCCCCCCCCCCCCCCC)COC(=O)CCCCCCCCCC

C51H98O6 TriacylglyceRFUHUUVPO=C(OCC(OC(=O)CCCCCCCCCCCCCC)COC(=O)CCCCCCCCCCCC
C51H98O6 TriacylglyceSRTVEGQF O=C(OCC(OC(=O)CCCCCCCCCCCCCCC)COC(=O)CCCCCCCCCCC
C51H98O6 TriacylglyceBRUJJDYLT O=C(OCC(OC(=O)CCCCCCCCCCCCCC)COC(=O)CCCCCCCCCCCC
C51H98O6 TriacylglyceSSSSKMPY O=C(OCC(OC(=O)CCCCCCCCCCCCCCCC)COC(=O)CCCCCCCCCC
C51H98O6 TriacylglyceUVJXULCL O=C(OCC(OC(=O)CCCCCCCCCCCCC)COC(=O)CCCCCCCCCCCCC
C51H98O6 TriacylglyceXHQYBFXV O=C(OCC(OC(=O)CCCCCCCCCCCCCCC)COC(=O)CCCCCCCCCCC
C53H98O6 TriacylglyceMTWYSKGJ O=C(OCC(OC(=O)CCCCCCCCCCCCCCC)COC(=O)CCCCCCCCCCC
C53H98O6 TriacylglyceTWJCMQZMO=C(OCC(OC(=O)CCCCCCCC=CCCCCCC)COC(=O)CCCCCCCCCC
C53H98O6 TriacylglyceQEZWFCZNO=C(OCC(OC(=O)CCCCCCCC=CCCCCCC)COC(=O)CCCCCCCCCC
C53H98O6 TriacylglyceODCBLKW O=C(OCC(OC(=O)CCCCCCCCCCCCC)COC(=O)CCCCCCCCCCCCC
C53H98O6 TriacylglyceTVENQJOA O=C(OCC(OC(=O)CCCCCCCCCCCCCCC)COC(=O)CCCCCCCCCC=
C53H98O6 TriacylglyceAFLXUGKIZ O=C(OCC(OC(=O)CCCCCCCC=CCCCCCCCC)COC(=O)CCCCCCCC
C53H98O6 TriacylglyceFYLBINGM O=C(OCC(OC(=O)CCCCCCCCCCCCC)COC(=O)CCCCCCCCCC=CC
C53H98O6 TriacylglyceDDNSMJIE O=C(OCC(OC(=O)CCCCCCCCCCCCC)COC(=O)CCCCCCCC=CCCC
C53H98O6 TriacylglyceKSDAXSHC O=C(OCC(OC(=O)CCCCCCCCCCCCC)COC(=O)CCCCCCCCCC=CC
C53H98O6 TriacylglycePQNLYGDUO=C(OCC(OC(=O)CCCCCCCCCCCCCCCCC)COC(=O)CCCCCCCCC
C53H100O TriacylglyceYHMDGPZ O=C(OCC(OC(=O)CCCCCCCCCCCCCCC)COC(=O)CCCCCCCCCCC
C53H100O TriacylglyceYSNGWXHI O=C(OCC(OC(=O)CCCCCCCC=CCCCCCC)COC(=O)CCCCCCCCCC
C53H100O TriacylglyceZKEWGTVA O=C(OCC(OC(=O)CCCCCCCCCCCCCCC)COC(=O)CCCCCCCCCCC
C53H100O TriacylglyceIFVQPZDH O=C(OCC(OC(=O)CCCCCCCCCCCCC)COC(=O)CCCCCCCCCCCCC
C53H100O TriacylglyceMITUBFKADO=C(OCC(OC(=O)CCCCCCCCCCCCC)COC(=O)CCCCCCCCCCCCC
C53H100O TriacylglyceFDCOHGH O=C(OCC(OC(=O)CCCCCCCC=CCCCCCCCC)COC(=O)CCCCCCCC
C53H100O TriacylglyceALZSQWYL O=C(OCC(OC(=O)CCCCCCCC=CCCCCCC)COC(=O)CCCCCCCCCC
C53H100O TriacylglyceSTJWLLYLX O=C(OCC(OC(=O)CCCCCCCCCCCCCCCCC)COC(=O)CCCCCCCCC
C53H100O TriacylglyceAJWXNNCJ O=C(OCC(OC(=O)CCCCCCCCCCCCCCC)COC(=O)CCCCCCCCCCC
C53H100O TriacylglyceBUBDIXSZFO=C(OCC(OC(=O)CCCCCCCCCCCCCCCCC)COC(=O)CCCCCCCCC
C24H66N16O7S5
C25H61N18O7P3S
C51H54N4O8
C57H54O7
C55H102O TriacylglyceMDCUASFVO=C(OCC(OC(=O)CCCCCCCCCCCCCCCCC)COC(=O)CCCCCCCCC
C55H102O TriacylglyceSZXNLYZN O=C(OCC(OC(=O)CCCCCCCCCCCCCCCCC)COC(=O)CCCCCCCC=
C55H102O TriacylglyceJFISYPWOVO=C(OCC(OC(=O)CCCCCCCC=CCCCCCCCC)COC(=O)CCCCCCCC
C55H102O TriacylglyceIHIYITLVGJ O=C(OCC(OC(=O)CCCCCCCC=CCCCCCC)COC(=O)CCCCCCCCCC
C55H102O TriacylglyceKKJVTOLW O=C(OCC(OC(=O)CCCCCCCC=CCCCCCC)COC(=O)CCCCCCCCCC
C55H102O TriacylglyceQOKAQJJB O=C(OCC(OC(=O)CCCCCCCCCCCCCCCCC)COC(=O)CCCCCCCCC
C55H102O TriacylglyceHYQDVSFOO=C(OCC(OC(=O)CCCCCCCCCCCCCCC)COC(=O)CCCCCCCCCC=
C55H102O TriacylglyceSFJDWDOXO=C(OCC(OC(=O)CCCCCCCCCCCCCCC)COC(=O)CCCCCCCCCC=
C55H102O TriacylglyceLYMKCBJXTO=C(OCC(OC(=O)CCCCCCCCCCCCCCC)COC(=O)CCCCCCCCCCC
C55H102O TriacylglyceQXBLJSPU O=C(OCC(OC(=O)CCCCCCCCCCCCCCCCC)COC(=O)CCCCCCCCC
C55H104O TriacylglyceNPCZZYKIT O=C(OCC(OC(=O)CCCCCCCCCCCCCCCCC)COC(=O)CCCCCCCCC
C55H104O TriacylglyceCDKITPQU O=C(OCC(OC(=O)CCCCCCCCCCCCCCCCC)COC(=O)CCCCCCCCC
C55H104O TriacylglyceXFUMCVM O=C(OCC(OC(=O)CCCCCCCC=CCCCCCC)COC(=O)CCCCCCCCCC
C55H104O TriacylglyceXRTUKMLV O=C(OCC(OC(=O)CCCCCCCCCCCCCCC)COC(=O)CCCCCCCCCCC

C55H104O TriacylglyceXWOGDCX O=C(OCC(OC(=O)CCCCCCCCCCCCCCCC)COC(=O)CCCCCCCCCCC
C55H104O TriacylglyceQXPXMOH O=C(OCC(OC(=O)CCCCCCCC=CCCCCCCCC)COC(=O)CCCCCCCC
C55H104O TriacylglyceHXIVIMYSQO=C(OCC(OC(=O)CCCCCCCC=CCCCCCCCC)COC(=O)CCCCCCCC
C55H104O TriacylglyceOJGNIFPC O=C(OCC(OC(=O)CCCCCCCCCCCCCCCCCC)COC(=O)CCCCCCCCC
C55H104O TriacylglyceXAHIOAND O=C(OCC(OC(=O)CCCCCCCCCCCCCCCCCC)COC(=O)CCCCCCCCC
C55H104O TriacylglyceXKNYUNQVO=C(OCC(OC(=O)CCCCCCCC=CCCCCCC)COC(=O)CCCCCCCCCC
C57H104O TriacylglyceYZEDZPDP O=C(OCC(OC(=O)CCCCCCCC=CCC=CCCCCC)COC(=O)CCCCCCC
C57H104O TriacylglyceMSQBYCW O=C(OCC(OC(=O)CCCCCCCC=CCC=CCCCCC)COC(=O)CCCCCCC
C57H104O TriacylglyceCNGSOZKJ O=C(OCC(OC(=O)CCCCCCCC=CCCCCCCCC)COC(=O)CCCCCCCC
C57H104O TriacylglyceOVEMPQU O=C(OCC(OC(=O)CCCCCCCC=CCCCCCCCC)COC(=O)CCCCCCCC
C57H104O TriacylglyceTYPSCZYH O=C(OCC(OC(=O)CCCCCCCCCCCCCCCC)COC(=O)CCCCCCCCCC=
C57H104O TriacylglyceIBHXNCOU O=C(OCC(OC(=O)CCCCCCCCCCCCCCCCCC)COC(=O)CCCCCCCCC
C57H104O TriacylglyceSAKURFW O=C(OCC(OC(=O)CCCCC=CCC=CCC=CCCCCC)COC(=O)CCCCCC
C57H104O TriacylglyceKQKXUUFGO=C(OCC(OC(=O)CCCCCCCCCCCCCCCCCC)COC(=O)CCCCCCCCC
C57H104O TriacylglyceFAWROKQ O=C(OCC(OC(=O)CCCCCCCCCCCCCCCCCC)COC(=O)CCCCCCCCC
C57H104O TriacylglyceQMOIIDCA O=C(OCC(OC(=O)CCCCCCCC=CCC=CCC=CCC)COC(=O)CCCCCC
C33H94N16O6P2S
C34H88N20O4S2
C34H96N16O3S4
C41H88N14O6S
C26H84N26O7S
C33H94N16O6P2S
C34H88N20O4S2
C39H101N8O6PS3
C14H16N5O11P5S10
C15H18N5O8P3S13
C16H20N5O5PS16
C39H101N8O6PS3

CCC1)CCCCN)CCCCN)CCCCN)CCCCN)CCCNC(=N)N)CNC(=O)CC(O)CCCC=CCCCCCC
